# *NEB* mutations disrupt the super-relaxed state of myosin and remodel the muscle metabolic proteome in nemaline myopathy

**DOI:** 10.1186/s40478-022-01491-9

**Published:** 2022-12-17

**Authors:** Natasha Ranu, Jenni Laitila, Hannah F. Dugdale, Jennifer Mariano, Justin S. Kolb, Carina Wallgren-Pettersson, Nanna Witting, John Vissing, Juan Jesus Vilchez, Chiara Fiorillo, Edmar Zanoteli, Mari Auranen, Manu Jokela, Giorgio Tasca, Kristl G. Claeys, Nicol C. Voermans, Johanna Palmio, Sanna Huovinen, Maurizio Moggio, Thomas Nyegaard Beck, Aikaterini Kontrogianni-Konstantopoulos, Henk Granzier, Julien Ochala

**Affiliations:** 1 Centre of Human and Applied Physiological Sciences, School of Basic and Medical Biosciences, Faculty of Life Sciences & Medicine, King’s College London, London, UK; 2 Department of Biomedical Sciences, University of Copenhagen, Copenhagen, Denmark; 3 The Folkhälsan Institute of Genetics and Department of Medical and Clinical Genetics, Medicum, Biomedicum Helsinki, University of Helsinki, Helsinki, Finland; 4 School of Sport, Exercise and Health Sciences, Loughborough University, Loughborough, UK; 5 Department of Biochemistry and Molecular Biology, University of Maryland School of Medicine, Baltimore, USA; 6 Department of Cellular and Molecular Medicine, University of Arizona, Tucson, USA; 7 Copenhagen Neuromuscular Center, Rigshospitalet, University of Copenhagen, Copenhagen, Denmark; 8 Neuromuscular and Ataxias Research Group, Instituto de Investigación Sanitaria La Fe, Valencia, Spain; 9 Centro de Investigación Biomédica en Red de Enfermedades Raras (CIBERER) Spain, Valencia, Spain; 10 Neuromuscular Disorders Unit, IRCCS Istituto Giannina Gaslini, DINOGMI, University of Genoa, Genoa, Italy; 11 Department of Neurology, Faculdade de Medicina (FMUSP), Universidade de São Paulo, São Paulo, Brazil; 12 Clinical Neurosciences, University of Helsinki and Helsinki University Hospital, NeurologyHelsinki, Finland; 13 Neurology, Clinical Medicine, University of Turku, Turku, Finland; 14 Neurocenter, Turku University Hospital, Turku, Finland; 15 Unità Operativa Complessa di Neurologia, Fondazione Policlinico Universitario “A. Gemelli”, IRCCS, Rome, Italy; 16 John Walton Muscular Dystrophy Research Centre, Newcastle University and Newcastle Hospitals NHS Foundation Trusts, Newcastle Upon Tyne, UK; 17 Department of Neurology, University Hospitals Leuven, Leuven, Belgium; 18 Laboratory for Muscle Diseases and Neuropathies, Department of Neurosciences, KU Leuven, Leuven, Belgium; 19 Department of Neurology, Donders Institute for Brain, Cognition and Behaviour, Radboud University Medical Center, Nijmegen, The Netherlands; 20 Neuromuscular Research Center, Department of Neurology, Tampere University and University Hospital, Tampere, Finland; 21 Department of Pathology, Fimlab Laboratories, Tampere University Hospital, Tampere, Finland; 22 Neuromuscular and Rare Diseases Unit, Department of Neuroscience, Fondazione IRCCS Ca’ Granda Ospedale Maggiore Policlinico, Milan, Italy

**Keywords:** Skeletal muscle, Nemaline myopathy, Nebulin, Myosin, Metabolism

## Abstract

Nemaline myopathy (NM) is one of the most common non-dystrophic genetic muscle disorders. NM is often associated with mutations in the *NEB* gene. Even though the exact *NEB*-NM pathophysiological mechanisms remain unclear, histological analyses of patients’ muscle biopsies often reveal unexplained accumulation of glycogen and abnormally shaped mitochondria. Hence, the aim of the present study was to define the exact molecular and cellular cascade of events that would lead to potential changes in muscle energetics in *NEB*-NM. For that, we applied a wide range of biophysical and cell biology assays on skeletal muscle fibres from NM patients as well as untargeted proteomics analyses on isolated myofibres from a muscle-specific nebulin‐deficient mouse model. Unexpectedly, we found that the myosin stabilizing conformational state, known as super-relaxed state, was significantly impaired, inducing an increase in the energy (ATP) consumption of resting muscle fibres from *NEB*-NM patients when compared with controls or with other forms of genetic/rare, acquired NM. This destabilization of the myosin super-relaxed state had dynamic consequences as we observed a remodeling of the metabolic proteome in muscle fibres from nebulin‐deficient mice. Altogether, our findings explain some of the hitherto obscure hallmarks of NM, including the appearance of abnormal energy proteins and suggest potential beneficial effects of drugs targeting myosin activity/conformations for *NEB*-NM.

**Supplementary Information:**

The online version contains supplementary material available at 10.1186/s40478-022-01491-9.

## Introduction

Nemaline myopathy (NM) is among the most common non-dystrophic genetic muscle disorders, with an estimated incidence of 1 in 20,000 live births [10, 15]. Clinical symptoms of NM include hypotonia, muscle weakness and fatigue [10, 15]. In the severe form, neonatal death may ensue whilst milder forms range from delayed motor developmental milestones to requiring a wheelchair, or even late-onset mild muscle dysfunction in adulthood [10, 15]. In all forms of NM, respiratory compromise is a risk throughout life [10, 15]. *NEB* mutations account for more than 50% of all NM cases [10]. These mutations result in shorter forms or haplo-insufficiency of the giant protein, nebulin, known to be an integral component of the thin filaments in skeletal muscle [10, 15]. Following this, we and others have observed that with shorter forms or reduced levels of nebulin, actin filament activation is incomplete. Subsequently, myosin motors cannot bind properly to actin monomers, which depresses the force-generating capacity of muscle fibres, thus causing muscle weakness in *NEB*-NM [17, 25, 26, 30]. Hence, we have previously targeted the force production of myosin proteins in NM utilizing a recombinant adeno-associated viral vector-related gene therapy and showed its promise in an animal model with a mutation in the NM-causing gene, ACTA1 [17]. However, this approach failed to restore muscle function in *NEB*-NM mouse models (unpublished data). This suggests that, despite major advancements in our understanding of the disease, NM pathophysiology is complex and far from being fully understood and consequently the design and implementation of accurate therapeutic interventions remains challenging [10].

Remarkably, ultrastructural and histological observations from *NEB*-NM patients and from relevant murine models not only shed light on the presence of nemaline rods (an important diagnostic tool for NM) but also on glycogen deposits and misshapen mitochondria with noticeable pleomorphism, concentric christae and increased subsarcolemmal crescents [32, 38]. In line with these observations, muscle glycolytic pathways have been found altered [34]. These findings indicate an under-appreciated potential change in muscle energetics and metabolism in *NEB*-NM as well as in other forms of NM caused by other gene mutations. Further support comes from clinical observations reporting that children and adolescents with NM are often lean despite their inability to engage in fast motor activities. Inefficient binding of the force-producing myosin molecules to actin filaments may contribute to altered energetics and metabolism in NM muscles by subtly increasing the energy (ATP) cost of contraction [25, 26]. Nevertheless, other more prominent pathological ATP-consuming mechanisms are likely to occur in *NEB*-NM. In the present study, we initially set forth to explore this hypothesis and study the involvement of resting myosin energetics, as an underlying NM mechanism.

Myosin has multiple chemo-mechanical states [6]. In addition to several active states, two distinctive relaxed states exist; myosin heads that are detached from actin filaments, and do not produce force, can be in either ‘super-relaxed’ or ‘disordered-relaxed’ states [9, 19]. In the super-relaxed state, myosin heads interact with the thick filament backbone restricting their interaction with actin. In the disordered-relaxed state, myosin molecules are not immobilized and can weakly bind actin allowing a fast transition to the active state when actin filaments are switched on. The fraction of myosin heads in disordered-relaxed and super-relaxed conformations correlates with the rate of ATP usage, with the ATPase activity of myosin heads in the disordered-relaxed configuration being ten times higher than this in the super-relaxed state [9, 19]. Thus, in the present study, we further hypothesized that in *NEB*-NM, the proportion of myosin molecules in the super-relaxed state is disrupted, impacting the basal ATP consumption of skeletal muscle, ultimately modifying the level of proteins involved in energy-producing pathways and contributing to the disease phenotype. To test this hypothesis, we used skeletal myofibres extracted from a wide spectrum of NM patients as well as from a muscle-specific nebulin conditional knockout mouse model (cNEB KO). We then performed a combination of biophysical assays, cell biology techniques and proteomics analyses. Interestingly, in line with our hypotheses, we found that in relaxed muscle fibres from NM patients, the myosin-stabilizing structural state is altered, with a potential causal involvement of myosin binding proteins such as regulatory light chains and myosin-binding protein C that are known to be involved in sequestering the super-relaxed state. We also observed that the increase in basal myosin ATP consumption may remodel muscle energy proteins, altogether paving the way to therapies related to myosin for NM.

## Materials and methods

### Human subjects

Muscle biopsy specimens were obtained from 26 NM patients with known mutations in either *NEB, ACTA1*, *TPM2* or *TPM3*; and 12 age-matched controls with no history of neuromuscular disease. Eleven additional NM patients had an extremely rare, late-onset acquired myopathy termed sporadic late-onset NM (SLONM) that is known to have similar histopathological abnormalities as genetic NM and that progresses subacutely [36]. All tissue was consented, stored, and used in accordance with the Human Tissue Act, UK, under local ethical approval (REC 13/NE/0373). Details of all the 49 individuals are given in Additional file 1: Table S1. All samples were flash-frozen and stored at − 80 °C until analyzed.

### Nebulin knockout mouse model

The conditional muscle-specific nebulin knockout mouse model used in the present study has previously been published in detail [16]. Briefly, mice were on a C57BL/6 J background. Floxed mice were bred to a MCK-Cre strain that expresses Cre recombinase under the control of the Muscle Creatine Kinase (MCK) promoter. Mice that were positive for MCK-Cre and homozygous for the floxed nebulin allele were nebulin deficient (cNeb KO). Mice with one nebulin wild-type allele and being either MCK-Cre positive or negative served as controls. All the experiments were approved by the University of Arizona Institutional Animal Care and Use Committee (09-056) and were in accordance with the United States Public Health Service’s Policy on Humane Care and Use of Laboratory Animals. At 6 months of age, six cNeb KO and six control female mice were weighed, anesthetized with isoflurane, and sacrificed by cervical dislocation. Tibialis cranialis skeletal muscles were then dissected and flash-frozen in liquid nitrogen before being stored at − 80 °C for later analysis.

### Solutions

As previously published [30], the relaxing solution contained 4 mM Mg-ATP, 1 mM free Mg^2+^, 10^–6.00^ mM free Ca^2+^, 20 mM imidazole, 7 mM EGTA, 14.5 mM creatine phosphate and KCl to adjust the ionic strength to 180 mM and pH to 7.0. Additionally, the rigor buffer for Mant-ATP chase experiments contained 120 mM K acetate, 5 mM Mg acetate, 2.5 mM K_2_HPO_4_, 50 mM MOPS, 2 mM DTT with a pH of 6.8. The lambda phosphatase solution (New England Biolabs) was prepared by a 100-fold dilution into the relaxing solution to yield 4 U lambda phosphatase/µl [29]. The solution for extracting myosin regulatory light chains (RLC) contained 20 mM EDTA, 50 mM KPr, 10 mM potassium phosphate buffer with a pH of 7.1 [5]. Finally, the solution for extracting myosin-binding protein C (MyBP-C) contained 10 mM EDTA, 31 mM Na_2_HPO_2_, 124 mM NaH_2_PO_4_, with a pH of 5.9 [7, 8].

### Muscle preparation and fibre permeabilisation

Cryopreserved human and mouse muscle samples were immersed in a membrane-permeabilising solution (relaxing solution containing glycerol; 50:50 v/v) for 24 h at − 20 °C, after which they were transferred to 4 °C and bundles of approximately 50–100 muscle fibres were dissected free. These bundles were kept in the membrane-permeabilising solution at 4 °C for an additional 24 h (to allow for a proper skinning/membrane permeabilisation process). After these steps, bundles were stored in the same buffer at − 20 °C for use up to 1 week [30, 31].

### Mant-ATP chase experiments

On the day of the experiments, bundles were transferred to relaxing solution and single myofibres were manually isolated. Their ends were individually clamped to half-split copper meshes designed for electron microscopy (SPI G100 2010C-XA, width, 3 mm), which had been glued to glass slides (Academy, 26 × 76 mm, thickness 1.00–1.20 mm). Cover slips were then attached to the top (using double-sided tape) to create flow chambers (Menzel-Glaser, 22 × 22 mm, thickness 0.13–0.16 mm) [22]. Muscle fibres were mounted at a relaxed length (with their sarcomere length checked using the brightfield mode of a Zeiss Axio Scope A1 microscope, approximately at 2.20 µm). Similar to previous studies [22], all experiments were performed at 25 °C, and each fibre was first incubated for 5 min with a rigor buffer. A solution containing the rigor buffer with 250 μM Mant-ATP was then flushed and kept in the chamber for 5 min. At the end of this step, another solution made of the rigor buffer with 4 mM unlabelled ATP was added with simultaneous acquisition of the Mant-ATP chase.

For fluorescence acquisition, a Zeiss Axio Scope A1 microscope was used with a Plan-Apochromat 20x/0.8 objective and a Zeiss AxioCam ICm 1 camera. Frames were acquired every 5 s for the first 90 s and every 10 s for the remaining time with a 20 ms acquisition/exposure time using a DAPI filter set, and images were collected for 5 min. Three regions of each individual myofibre were sampled for fluorescence decay using the ROI manager in ImageJ as previously published [22]. The mean background fluorescence intensity was subtracted from the average of the fibre fluorescence intensity (for each image taken). Each time point was then normalized by the fluorescence intensity of the final Mant-ATP image before washout (T = 0). These data were then fit to an unconstrained double exponential decay using Graphpad Prism 9.0:

$${\text{Normalised}}\;{\text{Fluorescence }} = \, 1 \, - {\text{ P}}1 \, \left( {1 - \exp^{{( - {\text{t}}/{\text{T}}1)}} } \right) \, - {\text{ P}}2 \, \left( {1 - \exp^{{( - {\text{t}}/{\text{T}}2)}} } \right),$$

where P1 is the amplitude of the initial rapid decay approximating the disordered-relaxed state with T1 as the time constant for this decay. P2 is the slower second decay approximating the proportion of myosin heads in the super-relaxed state with its associated time constant T2 [22].

### Immunofluorescence staining and imaging

To avoid any potential misinterpretation due to the type of myosin heavy chain, for the human Mant-ATP chase experiments, we assessed the sub-type using immunofluorescence staining as previously described [22]. Briefly, flow-chamber mounted myofibres were stained with an anti-β-cardiac/skeletal slow myosin heavy antibody (IgG1, A4.951, sc-53090 from Santa Cruz Biotechnology, dilution: 1:50) and an anti-slow myosin binding protein C antibody (IgG, SAB3501005 from Sigma, dilution: 1:200). Myofibres were then washed in PBS/0.025% Tween-20 and incubated with secondary antibodies: goat anti-mouse IgG1 Alexa 555 and goat anti-rabbit IgG Alexa 488 (from ThermoScientific, dilution 1:1000), respectively, in a blocking buffer. After washing, muscle fibres were mounted in Fluoromount. To identify the type of fibres, images were acquired using a confocal microscope (Zeiss Axiovert 200, 63 × oil objective) equipped with a CARV II confocal imager (BD Biosciences) [30, 31]. To obtain myosin filament length and myosin-binding protein C (MyBP-C) localisation measurements, mounted muscle fibres were acquired with a 100 × oil objective and an instant Structured Illumination Microscope (iSIM) system. To improve contrast and resolution (by two-fold compared to confocal microscopy), distributed deconvolution (DDecon) was then applied from the acquired images with a specific plugin for ImageJ (National Institutes of Health, Bethesda, MD) [24]. Note that DDecon is a super-resolution light microscopy technique that addresses light scattering, differences in refractive index, glare, and background noise. It also allows the computation of filament lengths with a precision of 10–20 nm [30, 31]. All line scans were background corrected. Distances and lengths were finally calculated by converting pixel sizes into µm using the scale for each image [30, 31].

### Western blotting

Lysates of the flash-frozen human muscle biopsy specimens from three control subjects and three *NEB*-NM patients were prepared via hand-homogenization in a modified NP-40 lysis buffer (10 mM NaH_2_PO_4_, pH 7.2, 2 mM EDTA, 10 mM NaN_3_, 120 mM NaCl, 0.5% deoxycholate, 1% NP-40) supplemented with complete protease inhibitor (Roche, Indianapolis, IN) and Halt phosphatase inhibitor (Thermo Scientific, Waltham, MA) cocktails. NuPage LDS sample buffer and reducing agent (Invitrogen, Waltham, MA) were added to 30 μg of protein lysate, boiled at 95 °C for 5 min, and fractionated by 4–12% SDS-PAGE. Protein was transferred to nitrocellulose membrane, blocked with 5% milk (RPI, Mt Prospect, IL) in TBST, and probed with the appropriate primary antibody: anti-slow myosin binding protein C (sMyBP-C, SAB3501005, Sigma-Aldrich, St. Louis, MO), anti-GAPDH (G7895, Sigma-Aldrich, St. Louis, MO), and custom phospho-sMyBP-C specific antibodies against mSer-59/hSer59 and mThr-84/hSer-82 as described previously [1]. Blots were then incubated with the appropriate horseradish peroxidase-conjugated secondary antibody (Cell Signaling Technology, Danvers, MA) and ECL substrate (Thermo Scientific, Waltham, MA). Densitometry was performed with ImageJ software. Total sMyBP-C blots produced a non-specific band of higher protein mass. Only the bottom specific band of the correct size was used for quantification. Relative sMyBP-C phosphorylation was calculated based on the sample’s total level of sMyBP-C following normalization to GAPDH loading control (Additional file 1: Fig. S1A–C).

### Enzymatic isolation and culture of intact mouse single muscle fibres

All animal procedures associated with enzymatic isolation of single muscle fibres were carried out at King’s College London in accordance with the UK Home Office regulations and in compliance with the European Community Directive published in 1989 (86/609/EEC). Two adult mature C57BL/6J mice were euthanized using cervical dislocation at 8 weeks of age. As previously published [30], Extensor digitorum longus (EDL) skeletal muscles were dissected, leaving tendons intact at both the proximal and distal ends. Subsequently, muscles were digested in 2 mg/mL collagenase I (Sigma Aldrich) in Dulbecco’s modified Eagle’s medium (DMEM; Invitrogen) for 105 min. Single fibres were released via trituration with a wide-bore glass pipette and hypo-contracted fibres and debris were removed following serial washes. Freshly isolated fibres were moved into six well plates; after 1 h they were dosed with either 100 µM of piperine or dimethyl sulfoxide (DMSO), which served as control, which was left for 3 days at 37 °C and 5% CO_2_. These fibres were then subjected to LC–MS/MS.

### LC–MS/MS identification and quantitative analysis of protein abundance

As previously published [28, 31], five samples for each experimental group were prepared. Each sample consisted of five single muscle fibres into a single centrifuge tube containing 30 μL Tris-Triton lysis buffer (10 mM Tris, pH 7.4, 100 mM NaCl, 1 mM EDTA, 1 mM EGTA, 1% Triton X-100, 10% glycerol, 0.1% SDS, 0.5% deoxycholate, protease inhibitor cocktail III (1:100), phosphatase inhibitor cocktail mix (1:100) at an unknown protein concentration). Sample volume was reduced by half in a SpeedVac (ThermoFisher Scientific) and subsequently mixed in a 1:1 ratio with Laemmli buffer (2 × conc.), vortexed and boiled at 96 °C for 10 min. To stack the protein complement and remove chemical interference from the lysis buffer, samples were centrifuged at 14,000 rpm for 3 min prior to loading in 10% BisTris gels (Gel 1—ThermoFisher Scientific #19072670-1957; Gel 2—#19072670-1965; Gel 3—#19072670-1966; Gel 4—#19072670-1977). Gels were then stained overnight with Imperial protein stain (ThermoFisher Scientific #24615). In-gel reduction, alkylation and digestion with trypsin was performed prior to subsequent isobaric mass tag labelling. Each sample was treated individually with labels (TMT10plex) added at a 1:1 ratio [4].

For analysis by LC–MS/MS, TMT labelled peptide samples were resuspended in 60 μL of resuspension buffer (2% ACN in 0.05% FA) with 10 μL sample injected in triplicate (30 μL total volume). Chromatographic separation was performed using an Ultimate 3000 NanoLC system (ThermoFisher Scientific). Peptides were resolved by reversed phase chromatography on a 75 μm × 50 cm C18 column using a three-step gradient of water in 0.1% formic acid and 80% acetonitrile in 0.1% formic acid. The gradient was delivered to elute the peptides at a flow rate of 250 nl/min over 250 min. The eluate was ionised by electrospray ionisation using an Orbitrap Fusion Lumos (ThermoFisher Scientific) operating under Xcalibur v4.1.

#### Database searching

Raw mass spectrometry data from the triplicate injection were processed into peak list files using Proteome Discoverer (ThermoScientific; v2.2) (PD 2.2). The data was processed and searched using the Mascot search algorithm (v2.6.0) and the Sequest search algorithm [4] against the Uniprot Mouse Taxonomy database (36,483 entries). Within the consensus processing module, the reporter ion intensity values (absolute area under the peak) for each peptide spectral match are grouped with peptides and calculated at the protein level identification as a grouped abundance. All grouped abundances at protein level are normalised using total peptide amount which has previously been corrected based on the highest peptide abundance present in one channel, thus all channels have the same total abundance.

#### Bioinformatics and data visualisations

Following processing with Proteome Discoverer, the resultant file was exported into Perseus (v1.6.3) for qualitative and quantitative data analysis. Metascape [46] was utilised for gene ontological (GO) analysis, which was subsequently visualised using cytoscape [37]. DAVID bioinformatic database was used for ligand binding analysis [39]. Further data visualisation utilised Biovinci v3.0.9 and Graphpad Prism v9.

#### Statistical analysis

Multiple myofibres were studied for each subject. Hence, as previously published [23], we used mixed linear models to statistically analyze the data. These models assumed that each subject had its own mean measurement (with a normal distribution between subjects) and that each measurement within a subject was also normally distributed around this mean. The *p* values tested the hypotheses that there were differences in these mean measurements between groups. Data are then presented as means ± standard deviations. Graphs were prepared and analyzed in Graphpad Prism v9. Statistical significance was set to *p* < 0.05. T-tests or One-way ANOVA with Tukey post-hoc were run to compare groups [30].

## Results

### Lower fraction of myosin molecules in the super-relaxed state with aberrant ATP consumption in human *NEB*-NM

**Fig. 1:**
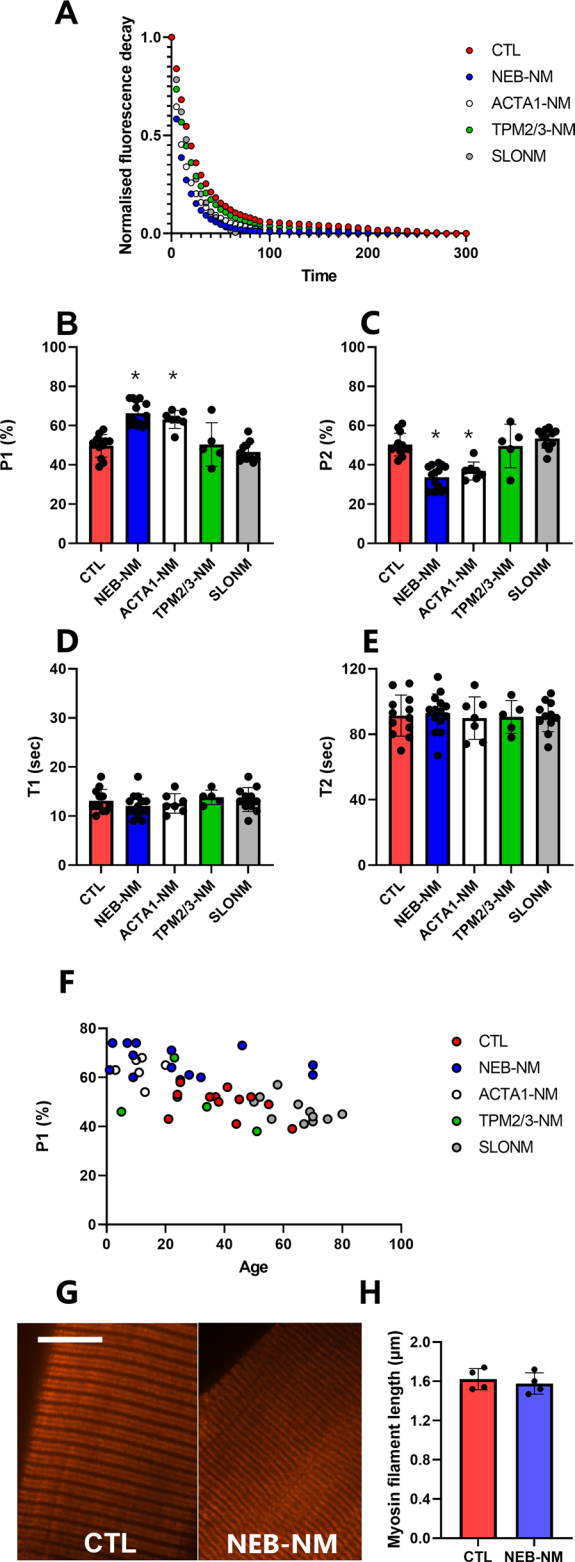
Myosin structure and relaxed conformational states in humans. Typical Mant-ATP chase experimental data show exponential decays for muscle fibres isolated from all the different groups (**A**). The proportion of myosin molecules in the disordered-relaxed (P1, **B**) and super-relaxed states (P2, **C**) as well as their respective ATP turnover lifetimes (T1, **D** and T2, **E**) are presented. **F** Shows the P1 data as a function of age. By staining and imaging myosin using super-resolution microscopy (**G**, scale bar = 10 µm) myosin filament length is calculated (**H**). Dots are individual subject’s average data. Means and standard deviations also appear on histograms. *Denotes a difference (*p* < 0.05) when compared with controls (CTL)

We first assessed the proportion of myosin heads in the super-relaxed state in human control and NM samples. Since this conformational state strongly correlates with the rate of ATP consumption in resting muscle fibres [40], we used a Mant-ATP chase protocol. As the scientific literature indicates that myofibres from NM patients mainly express the cardiac/skeletal slow myosin heavy chain [10], we restricted our analysis to this type of fibres. A total of 427 muscle fibres were tested (8–10 myofibres for each of the 12 controls and for each of the 37 patients—list of subjects in Additional file 1: Table S1). *NEB*-NM patients overall exhibited faster ATP consumption indicating significantly lower levels of myosin heads in the super-relaxed state when compared with controls (Fig. 1A–C). Despite this alteration, the actual ATP turnover time of super-relaxed myosin molecules was not affected (Fig. 1D, E). We repeated similar experiments in NM human tissue with mutations in the *ACTA1*, *TPM2* or *TPM3* genes (associated with defects in actin or actin-binding proteins, Additional file 1: Table S1). Interestingly, *ACTA1*-NM individuals displayed similar features as *NEB*-NM patients (Fig. 1A–E), whilst *TPM2*-NM and *TPM3*-NM were indistinguishable from controls (Fig. 1A–E). To gain insight into whether these alterations are specific to *NEB* (and *ACTA1*) gene mutations or related to NM-related histopathological changes, we studied myofibres from patients with an acquired form of the disease known as sporadic late-onset NM (SLONM). SLONM contrasts with typical genetic NM, since it occurs in the absence of any known mutations in NM-related genes. The onset of SLONM is also usually different as it starts in adulthood and progresses rapidly in a limb-girdle and axial pattern [36]. Nevertheless, histopathologically, nemaline rods, sarcomeric disarray and reduced cellular force-generating capacity have been observed [30]. SLONM patients did not exhibit any significant difference in the number/ATP turnover rate of myosin heads in the super-relaxed state when compared with controls, *TPM2*-NM and *TPM3*-NM patients (Fig. 1A–E). As the age range for the 37 patients with genetic or acquired NM varied between 1 and 70 years old, we tested whether our results are age dependent. Plotting myosin conformational state as a function of age did not reveal any long-term secondary adaptation (Fig. 1F).

To further evaluate if the changes in the presence of *NEB* mutations are due to disarray of myosin filaments, we used super-resolution microscopy followed by DDecon analysis. Thirty-five myofibres were tested from four controls and three *NEB*-NM patients (5 fibres per subject). Regular striated arrays of myosin filaments were observed for both patients and controls (Fig. 1G). Additionally, the length of these filaments had ranges that were consistent with the inter-individual and inter-muscle heterogeneity reported previously (Fig. 1H) [44].

These results indicate that, in the presence of *NEB* mutations, myosin heads are not properly sequestered (onto the myosin filament backbone) in resting skeletal muscle, consuming unusually large quantities of ATP. Our results also suggest that these alterations are not specific to *NEB* mutations but rather to myofilament-linked mechanisms that are common with *ACTA1* mutations. NM-associated histopathological disruptions such as the presence of nemaline rods may not be sufficient to drive the myosin maladaptations.

#### The decreased number of super-relaxed myosin molecules may be linked to some alterations affecting myosin-binding proteins in human *NEB*-NM and in a nebulin conditional knockout mouse model

**Fig. 2:**
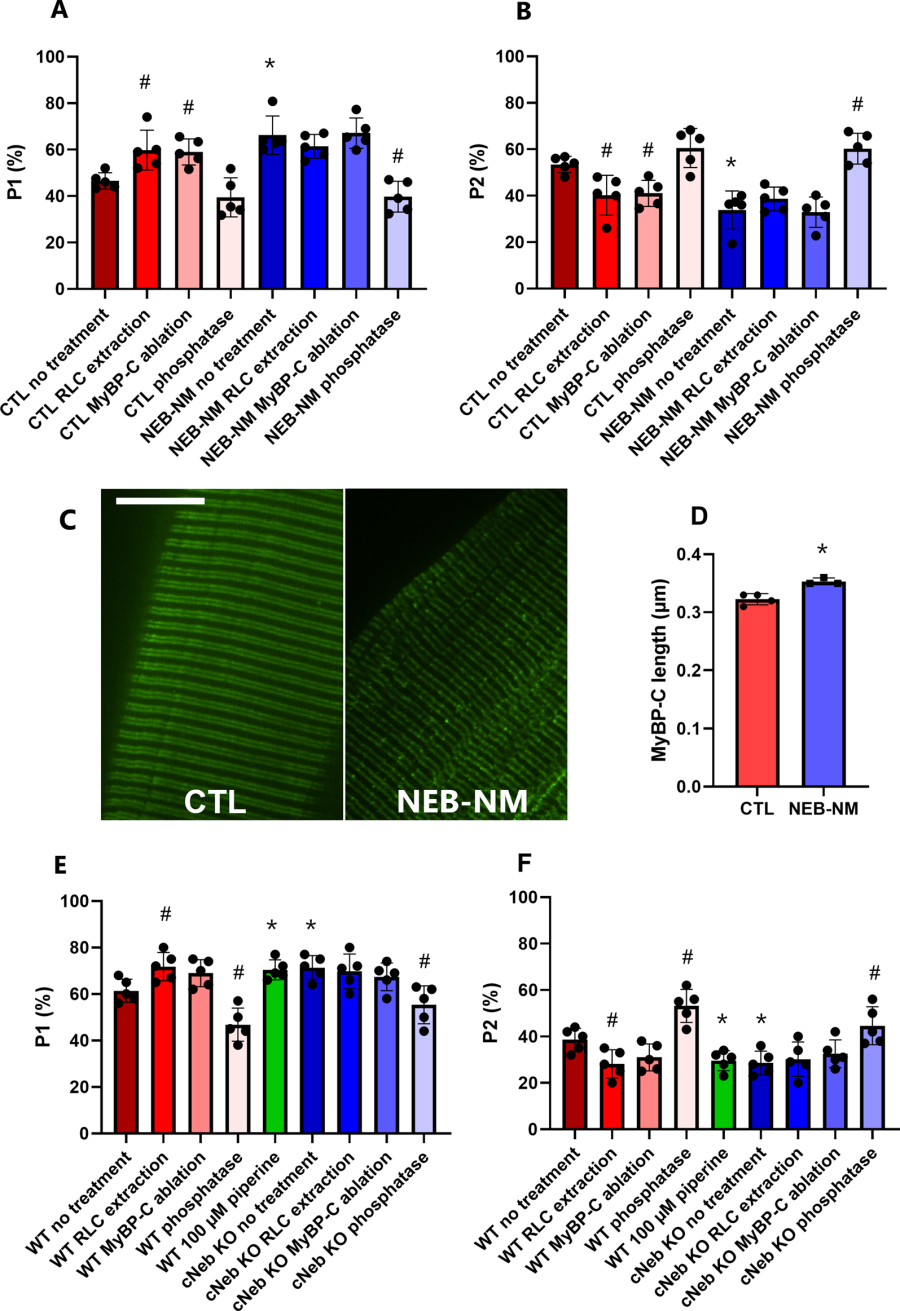
Modulation of Myosin Regulatory light chains (RLC) and Myosin-binding protein C (MyBP-C) levels and phosphorylations in humans and mice. The proportion of myosin heads in the disordered-relaxed (P1, **A**) and super-relaxed states (P2, **B**) are presented for humans. Dots are individual subject’s average data. **C** Is a typical super-resolution image (A, scale bar = 10 µm), and resultant MyBP-C filament length (**D**). Additionally, the proportion of myosin molecules in the disordered-relaxed (P1, **E**) and super-relaxed states (P2, **F**) for wild-type (WT) and transgenic (cNeb KO) mice are shown. Dots are individual mouse’s average data. Means and standard deviations also appear on all histograms. *Denotes a significant difference (*p* < 0.05) when compared with controls/WT with similar treatment. # refers to a significant difference (*p* < 0.05) when compared with before treatment for similar group (CTL/NEB-NM or WT/cNeb KO)

Myosin-binding protein C (MyBP-C) acts as a linker for myofilaments (between actin and myosin filaments); its role in stabilizing the myosin super-relaxed state has recently been highlighted by mutations in its core leading to deleterious hypertrophic cardiomyopathy [42]. Cardiac MyBP-C has then been extensively studied, whilst skeletal MyBP-C requires further attention [2]. As a proof-of-concept, to explore MyBP-C’s potential functional role in the increased disordered-relaxed state of myosin heads in *NEB*-NM, we first partially ablated the endogenous MyBP-C [7, 8] by using a published protocol consisting of soaking individual muscle fibres in an extracting buffer for 1-h at room temperature [7, 8]. The precise amount of MyBP-C ablated is thought to be more than 70% [7, 8] but was not directly evaluated in the present study as we did not get reproducible western blot/antibody data from single muscle fibres. A total of 91 myofibres were tested from five controls and five *NEB*-NM patients (8–10 fibres per subject). With the Mant-ATP chase protocol, MyBP-C partial ablation significantly decreased the number of myosin heads in the super-relaxed state in controls but not in *NEB*-NM patients (Fig. 2A, B). Hence, MyBP-C partial absence may alleviate the differences between *NEB*-NM patients and controls. Besides MyBP-C, other myosin-binding proteins may be involved. Myosin regulatory light chains (RLC) bind to the lever arm region of myosin heads and play an important role in maintaining the integrity of the super-relaxed state [45]. To, once again, explore whether RLC may also contribute to the changes seen in *NEB*-NM patients, we extracted the endogenous RLCs by incubating myofibres in a well-recognized extracting buffer for 30 min at 4 °C [5]. As for MyBP-C, the exact level of RLC extracted is thought to be more than 90% [5], nevertheless, we did not assess it here as we did not have reliable western blot/antibody results from individual myofibres. Fibres going through this process were then thoroughly washed with the rigor solution before running Mant-ATP chase experiments. A total of 72 myofibres were tested from five controls and five *NEB*-NM patients (7 to 8 fibres per subject). As for MyBP-C partial ablation, RLC partial extraction significantly lowered the proportion of myosin molecules in the super-relaxed state in controls but not in *NEB*-NM patients (Fig. 2A, B), indicating that RLC partial ablation may reduce the differences between *NEB*-NM patients and controls. As myofilament proteins are subject to multiple post-translational modifications impacting their function (especially MyBP-C and RLC), we explored whether modulating the phosphorylation status of the myofilament proteins would have effects on myosin head conformation in the patients. For that, we incubated myofibres in a lambda phosphatase solution for 1-h at room temperature [29]. The extent to which the lambda phosphatase solution lowers phosphorylation in individual myosin/myosin-binding proteins remains unclear as in the present study, we did not run western blots confirming the dephosphorylation. In total, 86 muscle fibres were used from five controls and five *NEB*-NM patients (8–10 fibres per subject). Mant-ATP chase experiments then revealed that the phosphatase treatment increased the proportion of myosin molecules in the super-relaxed conformation in *NEB*-NM patients (Fig. 2A, B). Importantly, the lambda phosphatase treatment dampened the differences observed between *NEB*-NM patients and controls.

As MyBP-C and RLC may have some functional implications in the disrupted super-relaxed state of *NEB*-NM patients, we imaged MyBP-C localization/disarray by applying super-resolution microscopy and DDecon analysis. 35 myofibres were tested from four controls and three *NEB*-NM patients (5 fibres per individual). Similar to myosin filaments, we observed regular MyBP-C striations in both patients and controls (Fig. 2C). Nevertheless, strikingly, the length of each MyBP-C segment was found subtly increased in *NEB*-NM patients when compared with controls (Fig. 2D). This suggests that MyBP-C localization extends beyond the C-zone in patients. To pursue MyBP-C investigations with the leftover human tissue, we measured the global content and phosphorylation levels (S59 and T84) for slow MyBP-C using Western blotting and antibodies known to work with human muscle samples. We found tendencies towards lower total abundance (Additional file 1: Fig. S1A) and higher phosphorylations (Additional file 1: Fig. S1B-C), even though data appeared patient-specific and thus overall variable.

To validate all the above human results, we intended to assess whether similar changes are recapitulated in a relevant mouse model of NM. Whilst the conventional *NEB* knockout model and a model in which exon 55 of the *NEB* gene is deleted exist, mice die within days after birth due to complex developmental defects and abnormalities [14]. As patients with *NEB* mutations often survive to adulthood with considerably milder myopathic phenotypes than the two mouse models described above, to investigate the consequences of *NEB* mutations/decreased myosin super-relaxed state, we took advantage of a conditional nebulin KO mouse model (cNeb KO) where muscle-specific deletions are present from birth [16]. We used 94 muscle fibres from five cNeb KO and five control mice (8 to 10 fibres per animal). We verified the presence of a myosin super-relaxed state destabilization in cNeb KO mice using the Mant-ATP chase protocol (Fig. 2E, F). Additional 154 mouse myofibres (5–6 fibres per mouse) were run where MyBP-C was partially ablated or RLC extracted or phosphorylation down-regulated using the same experimental protocols as for humans. Interestingly, we observed the same significant differences as for humans strengthening our findings (Fig. 2E, F).

Overall, our results indicate a potential role of MyBP-C deletion and/or RLC extraction and/or dephosphorylation in disrupting the myosin super-relaxed state and related ATP consumption in resting muscle fibres from *NEB*-NM patients and from cNeb KO mice.

#### The lower proportion of myosin heads in the super-relaxed conformation is associated with a metabolic remodeling in nebulin conditional knockout mouse model

**Fig. 3:**
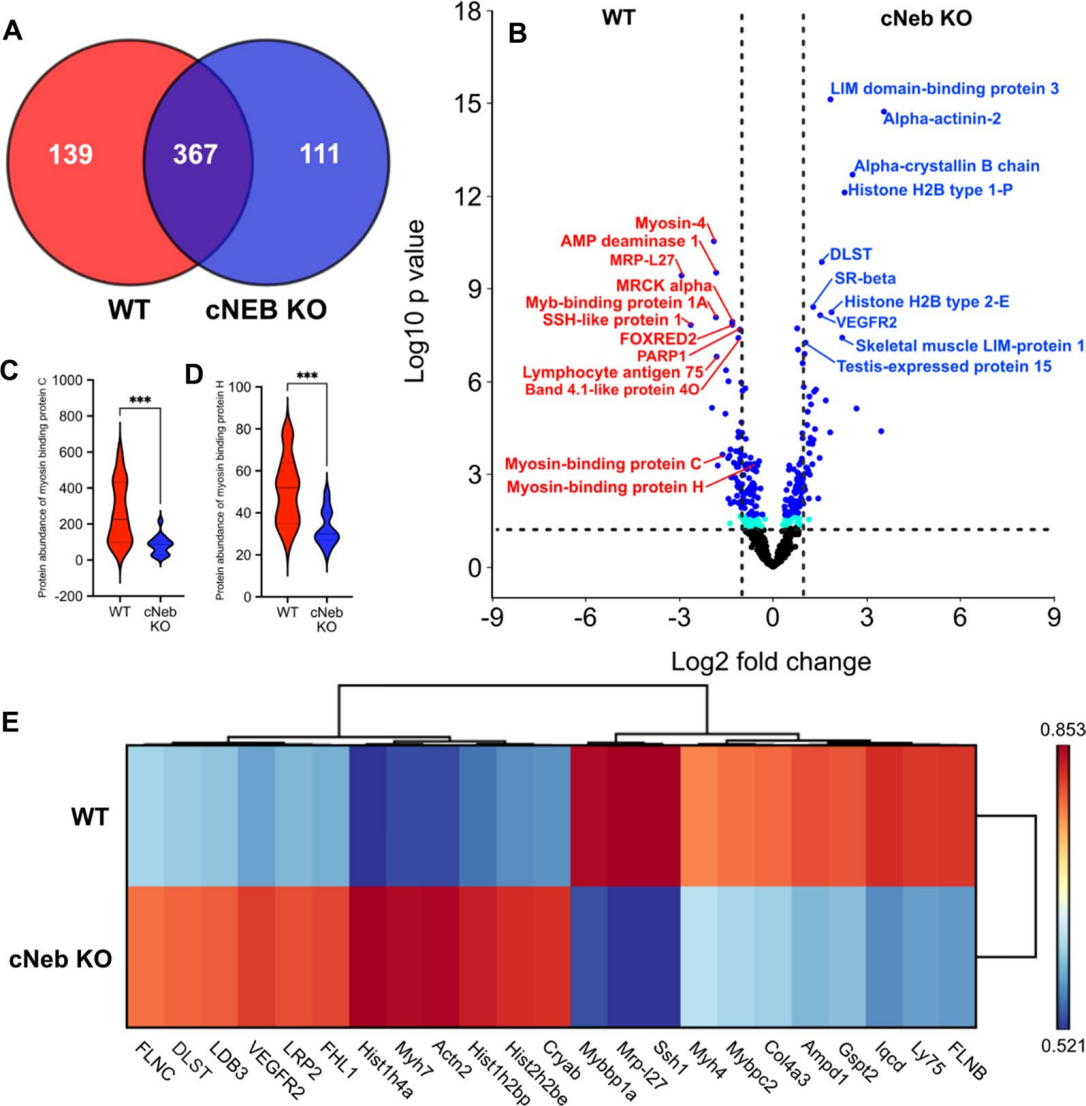
Comparative analysis of protein changes. **A** Venn diagram depicting detected proteins that possessed a significantly greater expression in WT (red) and cNEB KO (blue)respectively, as well as those unchanged between experimental groups. **B** Volcano plot displaying Log2 fold change against Log10 *p*-value. Dark blue dots indicate FDR (q value) < 0.05 whilst light blue dots indicate *p* < 0.05 and black dots indicate *p* > 0.05. The top 10 most significant proteins for each experimental group have been annotated in blue (cNEB KO) or red (WT) using protein names except were due to size constraints gene names were used (MRP-L27, MRCK alpha, FOXRED2, PARP1, DLST, SR-Beta and VEGFR2 (39S ribosomal protein L27-mitochondrial, Serine/threonine-protein kinase MRCK alpha, FAD-dependent oxidoreductase domain-containing protein 2, Poly[ADP-ribose] polymerase 1,2-Oxoglutarate Dehydrogenase Complex Component E2, Signal recognition particle receptor subunit beta, Vascular endothelial growth factor receptor 2, respectively). Myosin binding protein C and H were also annotated and their abundances are highlighted in the volcano plots in **C**, **D**. **E** A heat map was created to illustrate the proteins with the greatest fold change, all proteins included possessed a Log2 FC > 1.5. For readability gene names rather than protein names were included in the heap map the conversion between gene name and protein names can be found in Additional file 1: Table S2. ***Indicates *p* < 0.001

**Fig. 4:**
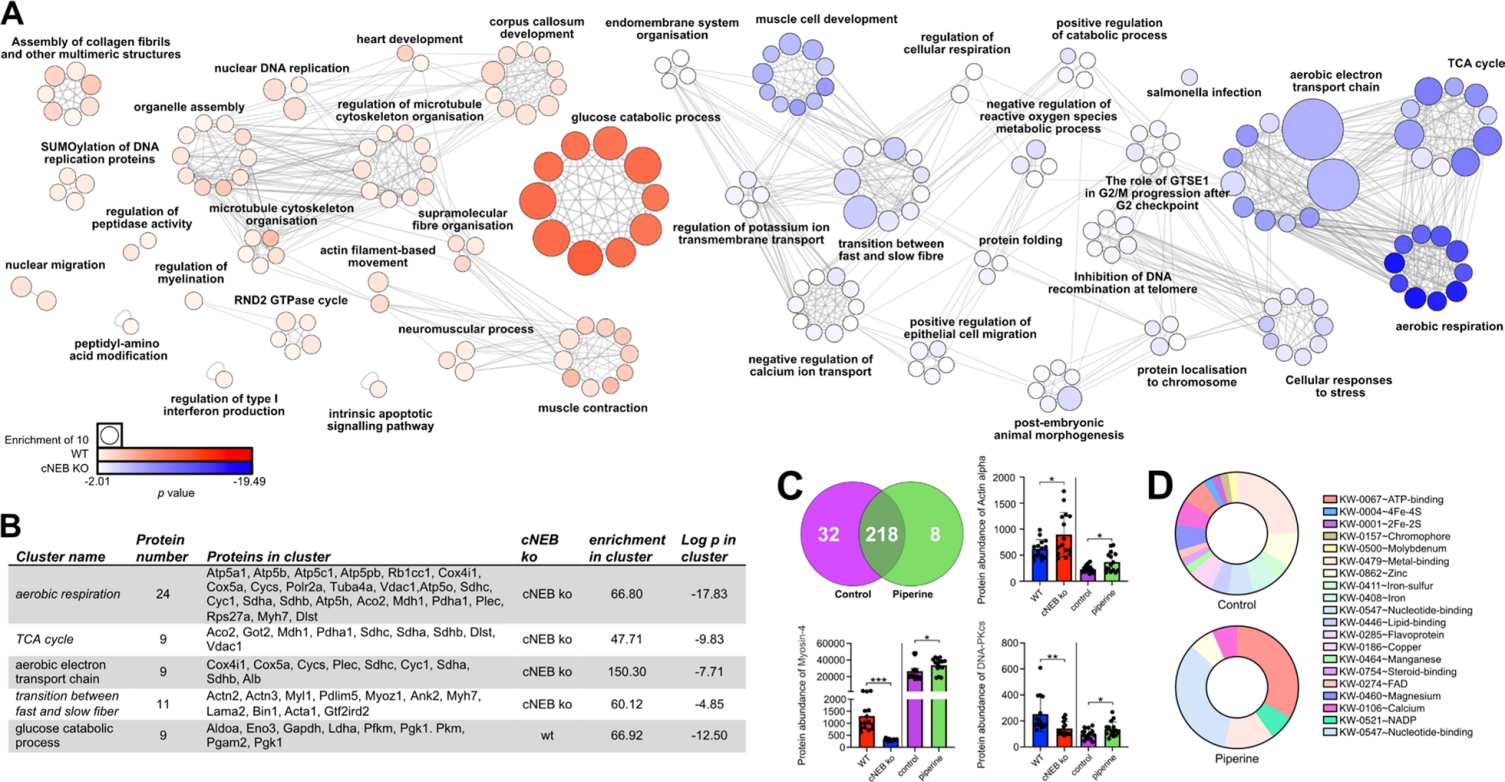
Ontological analysis of the proteins differentially expressed between WT and cNEB KO and proteomic differences induced following piperine administration. **A** Ontological associations between 111 proteins upregulated in cNEB KO (blue) and 139 proteins upregulated in WT mice (red) were established using Metascape and visualised using Cytoscape. Grey lines indicate a direct interaction, circle size is determined by enrichment and circle colour is determined by *p* value. Proteins upregulated in the WT mice can also be considered as down regulated in the cNEB KO. WT and cNEB KO networks were created separately with identical enrichment and *p* value scaling parameters. For graphical representation both WT and cNEB KO networks were scaled to match the enrichment key. The clusters with the highest enrichment are documented in the table shown in B, with the enrichment, *p* value and proteins present within the cluster. All full list of all clusters and proteins present is available in Additional file 1: Table S2 (**B**). **C** Venn diagram depicting detected proteins that were significantly expressed in control (purple) and following piperine administration (green), as well as those unchanged between experimental groups. Bar graphs depicting significant differences present in the three proteins which possessed significant differences between either WT and cNEB KO or between Control and Piperine administration, the two datasets were not subjected to comparative statistics. **D** Pie charts represent the Uniprot binding terms associated with the significantly up regulated proteins in either control or piperine administration obtained using DAVID bioinformatics database

To gain insights into the consequences on energy metabolism/usage of our findings, we pursued additional animal model experiments. We isolated individual limb (tibialis cranialis) muscle fibres from cNeb KO and control mice and ran a proteomics analysis through quantitative LC–MS/MS tandem mass spectrometry. To this end, we utilised manually dissected single fibres to reduce the influence of proteins from other tissue and cell types and to more closely correlate our findings with the above single fibre observations. We were able to quantify 617 proteins, of which, further filtration by p-value (*p* < 0.05) revealed that 250 proteins were differentially expressed, of these 111 and 139 were upregulated in the cNeb KO and control mice, respectively (Fig. 3A, Additional file 1: Table S2). We generated a volcano plot to visualize differentially upregulated proteins, annotated with the top 10 most significant proteins in each experimental group. Importantly, where a protein is denoted as more highly expressed in controls, it can also be interpreted as cNeb KO mice possessing a reduction in expression. The most significantly upregulated proteins belonged to the cNeb KO group and consisted of ZASP (Z-band Alternatively Spliced PDZ-motif) or LIM domain-binding protein 3 and alpha-actinin-2 with the latter possessing the highest log2FC (Fig. 3B). Moreover, we observed a significant change in myosin binding proteins H and C (Fig. 3B–D). Further, all proteins with a log2fc > 1.5 were visualised in the heatmap in (Fig. 3E), which highlighted a number of proteins involved in cytoskeletal structure but also and importantly metabolic pathways. To more accurately determine functional associations, we carried out gene ontology (GO) analysis on all proteins that passed p-value (*p* < 0.05) filtration using the Metascape analysis resource (Fig. 4A, B, Additional file 1: Table S3). Biological functions associated with metabolism appear distinct between cNeb KO and control muscles. A suppression of proteins involved in glucose catabolic processes was observed in cNeb KO mice when compared with controls. This was accompanied by an increase in aerobic respiration, aerobic electron transport chain and Tricarboxylic Acid Cycle (TCA) in cNeb KO mice when compared with controls. These findings, coupled with an increase in proteins associated with the transition between fast and slow fibre type pathways, indicate an alteration in ATP production in cNeb KO muscle towards higher yielding aerobic pathways. Fibres for these proteomic studies were all derived from the tibialis cranialis skeletal muscles where the fibre type is predominantly fast twitch. However, to determine whether fibre type sampling may be the underlying cause of these proteomic differences fibre type classification using myosin quantities was performed [11]. All samples were categorised as fast glycolytic fibres (IIx/IIb) (Additional file 1: Table S4). Additionally, following Pearson correlation analysis (Additional file 1: Fig. S2), all samples were more similar based on genomic background than fibre type.

Next, we wanted to determine whether the changes seen above in cNeb KO mice were related to the decrease in the number of myosin heads in the super-relaxed state. To achieve this, we used mouse extensor digitorum longus control muscles (this is a fast-twitch limb skeletal muscle as tibialis cranialis) and incubated them with piperine, an alkaloid found in pepper which has previously been observed to reduce the number of myosin molecules in the super-relaxed state [21, 43]. After three days of incubation in 100 µM of piperine (optimal concentration to destabilise the super-relaxed state—confirmed in Fig. 2E, F), we again carried out LC–MS/MS tandem mass spectrometry. A total of 260 proteins were detected following low TMT exclusion. However only 42 significant protein changes were observed following *p*-value (*p* < 0.05) filtration. The Venn diagram revealed that the 34 proteins significantly increased expression in the control group, whist only 8 were observed to have increased significantly following piperine administration (Fig. 4, Additional file 1: Table S5). Of these significant proteins, only three were also differentially expressed in the cNeb KO mice versus control database (Myosin-4, DNA-dependent protein kinase catalytic subunit and actin, alpha skeletal muscle). Due to the low number of significant differences, performing similar GO analysis as with the previous dataset was not possible, instead we aimed to determine whether there was an upregulation of proteins associated with specific protein–ligand interactions that may be ultimately responsible for the differences associated with the previously observed, chronic decrease in the myosin super-relaxed state. Indeed, while control fibres possessed an array of different ligand binding, piperine administration predominantly upregulated proteins which possessed ATP/nucleotide binding (Fig. 4D, Additional file 1: Table S6). These proteins may therefore be involved in detecting early dysregulation of ATP utilization present following the disturbance of myosin conformational states.

## Discussion

Our study is one of the first to characterise the myosin super-relaxed state in human skeletal muscle, as most of the scientific literature thus far is on cardiac tissue. We demonstrate that isolated muscle fibres from humans diagnosed with *NEB*-NM have a surprising destabilization of myosin super-relaxed state and excessive energy consumption. Consistent with these observations, we indicate that such ATP overconsumption has potential consequences on the myofibre proteome of a mouse NM model.

### Myosin super-relaxed state destabilization as a pathological contributor and/or a compensatory process

The super-relaxed conformation is a highly conserved and regulated state [35]. Its dysregulation in the context of skeletal muscle diseases is a novel finding. A reduction in the super-relaxed state is involved in the aetiology of other genetic diseases of cardiac muscle, such as hypertrophic cardiomyopathy (HCM). HCM is estimated to affect at least 1 in 500 individuals and is primarily caused by mutations in genes encoding the human β-cardiac/skeletal slow myosin heavy chain (*MYH7*) or cardiac MyBP-C (*MYBPC3*) [41, 42]. The subtle pathogenic amino acid substitutions in the β-cardiac/skeletal slow myosin heavy chain mesa region destabilize the inter-head motif area crucial for forming and preserving the super-relaxed state [3, 27, 41], whilst variants in *MYBPC3* cause truncations and haploinsufficiency in cardiac MyBP-C, releasing its restricting power on myosin heads and lowering the number of myosin molecules in the super-relaxed conformation [42]. All these are recognised as major components of the hyper-contractile HCM pathophysiology accounting for impaired cellular relaxation and enhanced force-generating capability [35]. Hence, here, it is reasonable to postulate that the decreased level of myosin heads in the super-relaxed state that we observed in *NEB*-NM (and *ACTA1*-NM) patients contribute to the aetiology of hypo-contractile NM through under-appreciated metabolic changes. More precisely, its involvement may be complex and may initially be a compensatory mechanism by which muscle fibres have more myosin heads available for actin binding to account for the depressed actin filament activation and cellular force-producing capacity [17, 25, 26, 30]. Indeed, disordering of myosin heads is proposed to facilitate the interaction of myosin with actin [19]. These interactions would be in weakly bound states that do not generate force but would contribute to stiffness [19]. In the long-term, this contractile over-compensation may become detrimental. Myosin super-relaxed state destabilization in *NEB*-NM (and *ACTA1*-NM) patients may have major consequences on ATP consumption and muscle metabolism, straining energy resources. This would be in line with the glycogen deposition and misshapen mitochondria observed in some patients and in most of the mouse models [18, 33].

### Consequences: metabolic reprogramming when the myosin super-relaxed state is downregulated

Although more comprehensive studies are warranted, shifting myosin heads away from their super-relaxed conformation means excessive energy consumption and most likely explains the profound abnormalities in energy usage seen in *NEB*-NM (and *ACTA1*-NM) patients’ muscle biopsy specimens [18, 33]. Skeletal muscle depends on a large number of pathways to produce ATP with cellular respiration being the most efficient machinery, supplying more than 90% of the basal energy requirements [6]. In the present study, we observed, in the presence of abnormal nebulin content, a metabolic reprogramming consisting of a shift away from glycolytic pathways to mitochondrial oxidative phosphorylation to meet the increased energy demands. This may have potential whole-body consequences. On average, adult humans utilise 8 MJ day^−1^. Most of this utilisation, known as the basal metabolic rate, is required for basic cellular functions. Even though the resting skeletal muscle metabolic rate per unit volume is low (0.5 W kg^−1^), it accounts for approximately 25% of the obligatory whole-body thermogenesis [47]. Here, the disordered myosin heads in patients may generate a greater overall thermogenesis [19]. Shifting myosin heads away from their super-relaxed conformation by as little as 10% may induce an increase in thermogenesis and energy usage by 0.7 MJ day^−1^ [19]. Over a period of a year this would lead to a weight loss of 7 kg of fat [19]. Shifting heads towards a disordered conformation by 20%, as found in the present work, would double skeletal muscle thermogenesis and would increase the whole-body basal metabolic rate by 16% [40]. This would explain clinical findings reporting NM patients being lean or underweight.

### Causes: myosin-binding protein disruption as a potential contributor of the decreased number of super-relaxed myosin molecules

In contrast to HCM, in the present study, the downregulation of the super-relaxed conformation in *NEB*-NM (and *ACTA1*-NM) cannot be attributed to the mutations but rather to indirect processes that could interfere with the levels of myofilament proteins. Our proteomics analysis has confirmed reductions of fast MyBP-C and fast RLC contents together with an up-regulation of myosin essential light chains (ELC) 1/3 in the presence of nebulin mutation. Thus, here, we explored the potential roles of myosin-binding proteins in *NEB*-NM. We observed significant functional differences when MyBP-C is partially ablated, RLC extracted or myofilament dephosphorylated. According to the literature, modulating the numbers of cardiac RLC or MyBP-C modifies the number of myosin molecules in the super-relaxed state by destabilizing the thick filaments, untethering myosin heads [12, 20, 45]. Moreover, when comparing the phosphorylated state of cardiac MyBP-C to its dephosphorylated state, it has been shown that the phosphorylated state promotes a higher myosin order whilst the phosphomimetic state favours disordered myosin indicative of a decreased proportion of myosin heads in the super-relaxed state [13]. Considering all these findings, it is tempting to suggest that RLC or MyBP-C are involved in the depression of the super-relaxed conformation in *NEB*-NM. The low number of patients tested for our MyBP-C- and RLC-related experiments as well as the absence of precise characterisations of MyBP-C and RLC deletions in our functional assays are obvious limitations here. Hence, further studies specifically focusing their attention on these aspects are required.

## Conclusion

Taken together, our data show that, in resting muscle fibres from *NEB*-NM patients, the myosin-stabilizing conformational state is disrupted. Our findings also suggest that the subsequent significant increase in basal ATP consumption leads to a modification of the myofibre proteome, more specifically of energy proteins. Our results then give new unexpected insights into unexplained *NEB*-NM pathological features, namely odd appearance of energetic proteins, and further highlight the potential benefits of drugs targeting myosin activity in NM patients.

## Supplementary Information

**Additional file 1: Fig. S1.** Myosin-binding protein C (MyBP-C) content and phosphorylation level. **A**–**C** display typical western blots and data normalized to GAPDH with total slow MyBP-C (**A**), S59 (**B**) and T84 (**C**) phosphorylation levels. **Fig. S2.** Pearson Correlations. Each individual sample was compared to each other, revealing samples in the same experimental groups are more similar to each other than any other fibres in the opposing experimental group. This includes WT_2 fibre, which was calculated to be a pure type 2x fibre, similar to all other cNEB KO myofibres. **Table S1.** Patient and control muscle biopsy samples used. **Table S2.** All proteins detected in manually dissected fibres originating from WT and cNEB KO mice. All proteins detected during LC-MS/MS tandem mass spectrometry, following filtration of missing values. #protein abundance values. Significant upregulation in each experimental group determined based on *p* < 0.05. **Table S3.** Proteins assigned to functional clusters detected in manually dissected fibres originating from WT and cNEB KO mice. Metascape determined both enrichment and *p* values for protein clusters and associated proteins. Visual representation of pathway clusters found in Fig. 4. **Table S4.** Fibre type classification. Overall fibre type per sample was calculated utilizing protein abundances from mass spectrometry and previously outlined myosin heavy chain percentages for each fibre type. **Table S5.** All proteins detected in WT enzymatically disassociated fibres with and without piperine administration. All proteins detected during LC-MS/MS tandem mass spectrometry, following filtration of missing values. ^#^Protein abundance values. Significant upregulation in each experimental group determined based on *p *< 0.05. **Table S6.** Uniprot ligand binding for significant proteins detected in WT enzymatically disassociated fibres with and without piperine administration. Likely ligand binding for all significant proteins (*p *< 0.05) obtained with and without piperine administration determined via the DAVID bioinformatic database.

## Data Availability

The proteomics datasets supporting the conclusions of this article are included in the supplementary file.
