# Supplementary material for: NEB mutations disrupt the super-relaxed state of myosin and remodel the muscle metabolic proteome in nemaline myopathy
Source: Acta Neuropathol Commun. 2022 Dec 17;10:185. doi: 10.1186/s40478-022-01491-9 (PMC9758823; doi:10.1186/s40478-022-01491-9)
Supplement: Supplementary file 1 — Additional file 1: Fig. S1. Myosin-binding protein C (MyBP-C) content and phosphorylation level. A–C display typical western blots and data normalized to GAPDH with total slow MyBP-C (A), S59 (B) and T84 (C) phosphorylation levels. Fig. S2. Pearson Correlations. Each individual sample was compared to each other, revealing samples in the same experimental groups are more similar to each other than any other fibres in the opposing experimental group. This includes WT_2 fibre, which was calculated to be a pure type 2x fibre, similar to all other cNEB KO myofibres. Table S1. Patient and control muscle biopsy samples used. Table S2. All proteins detected in manually dissected fibres originating from WT and cNEB KO mice. All proteins detected during LC-MS/MS tandem mass spectrometry, following filtration of missing values. #protein abundance values. Significant upregulation in each experimental group determined based on p < 0.05. Table S3. Proteins assigned to functional clusters detected in manually dissected fibres originating from WT and cNEB KO mice. Metascape determined both enrichment and p values for protein clusters and associated proteins. Visual representation of pathway clusters found in Fig. 4. Table S4. Fibre type classification. Overall fibre type per sample was calculated utilizing protein abundances from mass spectrometry and previously outlined myosin heavy chain percentages for each fibre type. Table S5. All proteins detected in WT enzymatically disassociated fibres with and without piperine administration. All proteins detected during LC-MS/MS tandem mass spectrometry, following filtration of missing values. #Protein abundance values. Significant upregulation in each experimental group determined based on p < 0.05. Table S6. Uniprot ligand binding for significant proteins detected in WT enzymatically disassociated fibres with and without piperine administration. Likely ligand binding for all significant proteins (p < 0.05) obtained with and without pipe [file 40478_2022_1491_MOESM1_ESM.docx]

***NEB* mutations disrupt the super-relaxed state of myosin and remodel the muscle metabolic proteome in nemaline myopathy**

**Supplementary Figure 1**

**Myosin-binding protein C (MyBP-C) content and phosphorylation level.**

(A), (B) and (C) display typical western blots and data normalized to GAPDH with total slow MyBP-C (A), S59 (B) and T84 (C) phosphorylation levels.

**Supplementary Figure 2**

**Pearson Correlations.**

Each individual sample was compared to each other, revealing samples in the same experimental groups are more similar to each other than any other fibres in the opposing experimental group. This includes WT_2 fibre, which was calculated to be a pure type 2x fibre, similar to all other cNEB KO myofibres.

**Supplementary Table 1**

**Patient and control muscle biopsy samples used.**

| **Age (years)** | **Gender** | **Mutation** | **Disease** | **Source** |
| --- | --- | --- | --- | --- |
| 70 | Female | *NEB* (c.22594C>T and c.5238+335_5764-407del) | Nemaline myopathy | Helsinki, Finland |
| 28 | Female | *NEB* (c.2836-2A>G and c.5763+5G>A) | Nemaline myopathy | Copenhagen, Denmark |
| 46 | Male | *NEB* (c.17234C>T and c.2271_22713del) | Nemaline myopathy | Copenhagen, Denmark |
| 32 | Male | *NEB* (c.23526_23527del and c.24792_24793del) | Nemaline myopathy | Helsinki, Finland |
| 22 | Male | *NEB* (c.12667G>A) | Nemaline myopathy | Valencia, Spain |
| 2 | Male | *NEB* (c.4082+5G>T) | Nemaline myopathy | Milan, Italy |
| 70 | Female | *NEB* (c.19097G>T and c.508-7T>A) | Nemaline myopathy | Helsinki, Finland |
| 22 | Male | *NEB* (c.18164T>C and c.15973C>T) | Nemaline myopathy | Valencia, Spain |
| 9 | Male | *NEB* (c.12676_12677delTA and c.10357A>C) | Nemaline myopathy | Valencia, Spain |
| 10 | Female | *NEB* (c.4489A>G and c.11947_11948 in sAGGACTATG) | Nemaline myopathy | Sao Paulo, Brazil |
| 1 | Male | *NEB* (c.7609delA and c.3593A>G) | Nemaline myopathy | Sao Paulo, Brazil |
| 7 | Male | *NEB* (c.19101+5G>A and c.9723+1G>A) | Nemaline myopathy | Sao Paulo, Brazil |
| 9 | Female | *NEB* (c.19102-4del and c.8889+1G>A) | Nemaline myopathy | Sao Paulo, Brazil |
| 25 | Female | *NEB* (Deletion of exons 11 to 107) | Nemaline myopathy | Tampere, Finland |
| 12 | Male | *ACTA1* (c.487C>CG) | Nemaline myopathy | Sao Paulo, Brazil |
| 3 | Male | *ACTA1* (c.611C>T) | Nemaline myopathy | Sao Paulo, Brazil |
| 13 | Male | *ACTA1* (c.487C>CG) | Nemaline myopathy | Sao Paulo, Brazil |
| 11 | Female | *ACTA1* (c.158A>T) | Nemaline myopathy | Sao Paulo, Brazil |
| 20 | Male | *ACTA1* (c.16G>A) | Nemaline myopathy | Copenhagen, Denmark |
| 3 | Male | *ACTA1* (c.235A>G) | Nemaline myopathy | Genoa, Italy |
| 10 | Male | *ACTA1* (c.841T>C) | Nemaline myopathy | Genoa, Italy |
| 5 | Female | *TPM2* (c.541_542invGA>AG) | Nemaline myopathy | Helsinki, Finland |
| 24 | Female | *TPM3* (c.301G>A) | Nemaline myopathy | Helsinki, Finland |
| 34 | Female | *TPM3* (c.301G>A) | Nemaline myopathy | Helsinki, Finland |
| 23 | Female | *TPM3* (c.301G>A) | Nemaline myopathy | Helsinki, Finland |
| 51 | Female | *TPM3* (c.301G>T) | Nemaline myopathy | Tampere, Finland |
| 65 | Male | - | Sporadic late onset nemaline myopathy | Rome, Italy |
| 69 | Female | - | Sporadic late onset nemaline myopathy | Rome, Italy |
| 50 | Male | - | Sporadic late onset nemaline myopathy | Rome, Italy |
| 56 | Female | - | Sporadic late onset nemaline myopathy | Leuven, Belgium |
| 52 | Female | - | Sporadic late onset nemaline myopathy | Leuven, Belgium |
| 58 | Male | - | Sporadic late onset nemaline myopathy | Nijmegen, Netherlands |
| 70 | Female | - | Sporadic late onset nemaline myopathy | Tampere, Finland |
| 75 | Female | - | Sporadic late onset nemaline myopathy | Tampere, Finland |
| 67 | Female | - | Sporadic late onset nemaline myopathy | Tampere, Finland |
| 70 | Male | - | Sporadic late onset nemaline myopathy | Tampere, Finland |
| 80 | Female | - | Sporadic late onset nemaline myopathy | Tampere, Finland |
| 55 | Male | - | - | London, UK |
| 25 | Female | - | - | London, UK |
| 63 | Female | - | - | London, UK |
| 37 | Male | - | - | London, UK |
| 44 | Female | - | - | London, UK |
| 38 | Female | - | - | Copenhagen, Denmark |
| 45 | Female | - | - | Copenhagen, Denmark |
| 49 | Male | - | - | Copenhagen, Denmark |
| 21 | Female | - | - | Copenhagen, Denmark |
| 24 | Female | - | - | Copenhagen, Denmark |
| 35 | Female | - | - | Copenhagen, Denmark |
| 41 | Female | - | - | Copenhagen, Denmark |

**Supplementary Table 2**

**All proteins detected in manually dissected fibres originating from WT and cNEB KO mice.**

All proteins detected during LC-MS/MS tandem mass spectrometry, following filtration of missing values. #protein abundance values. Significant upregulation in each experimental group determined based on *p* < 0.05.

| ***Gene name*** | ***Uniprot ID*** | ***Protein name*** | ***Control ^#^*** | ***cNEB ^#^*** | ***Log_2_ FC*** | ***p value*** | ***q value*** |
| --- | --- | --- | --- | --- | --- | --- | --- |
| **UPREGULATED IN WT** | | | | | | | |
| Myh4 | Q5SX39 | Myosin-4 | 1304.200 | 317.133 | -1.894 | < 0.001 | < 0.001 |
| Ckm | P07310 | Creatine kinase M-type | 1988.800 | 1374.733 | -0.573 | 0.003 | 0.010 |
| Aldoa | P05064 | Fructose-bisphosphate aldolase A | 868.200 | 542.600 | -0.719 | < 0.001 | 0.002 |
| Tpm1 | P58771 | Tropomyosin alpha-1 chain | 340.800 | 234.600 | -0.484 | 0.006 | 0.017 |
| Mybpc2 | Q5XKE0 | Myosin-binding protein C, fast-type | 259.867 | 83.200 | -1.617 | < 0.001 | 0.002 |
| Mylpf | P97457 | Myosin regulatory light chain 2, skeletal muscle isoform | 583.933 | 444.600 | -0.520 | 0.019 | 0.045 |
| Eno3 | P21550 | Beta-enolase | 634.333 | 397.933 | -0.722 | 0.003 | 0.010 |
| Gapdh | P16858 | Glyceraldehyde-3-phosphate dehydrogenase | 471.800 | 323.533 | -0.608 | 0.002 | 0.008 |
| Tnnt3 | Q9QZ47 | Troponin T, fast skeletal muscle | 652.600 | 455.867 | -0.493 | 0.001 | 0.003 |
| Casq1 | O09165 | Calsequestrin-1 | 439.133 | 371.933 | -0.260 | 0.028 | 0.061 |
| Pfkm | P47857 | ATP-dependent 6-phosphofructokinase, muscle type | 385.533 | 291.000 | -0.446 | < 0.001 | 0.002 |
| Pkm | P52480 | Pyruvate kinase PKM | 174.733 | 115.600 | -0.588 | 0.001 | 0.003 |
| Pgam2 | O70250 | Phosphoglycerate mutase 2 | 180.333 | 117.533 | -0.632 | 0.029 | 0.061 |
| Ldha | P06151 | L-lactate dehydrogenase A chain | 58.600 | 30.600 | -0.909 | 0.012 | 0.031 |
| Pgk1 | P09411 | Phosphoglycerate kinase 1 | 315.933 | 232.200 | -0.514 | 0.028 | 0.061 |
| Mybph | P70402 | Myosin-binding protein H | 50.333 | 32.733 | -0.597 | < 0.001 | 0.003 |
| Ampd1 | Q3V1D3 | AMP deaminase 1 | 134.867 | 38.667 | -1.821 | < 0.001 | > 0.001 |
| Pdlim7 | Q3TJD7 | PDZ and LIM domain protein 7 | 142.333 | 97.733 | -0.645 | 0.002 | 0.008 |
| Flnb | Q80X90 | Filamin-B | 97.467 | 23.333 | -1.769 | 0.001 | 0.003 |
| Serpinb6 | Q60854 | Serpin B6 | 45.267 | 32.067 | -0.669 | 0.033 | 0.067 |
| Myh14 | Q6URW6 | Myosin-14 | 45.467 | 25.333 | -0.785 | 0.049 | 0.094 |
| Mylk | Q6PDN3 | Myosin light chain kinase, smooth muscle | 29.600 | 21.000 | -0.585 | 0.031 | 0.064 |
| Tln2 | Q71LX4 | Talin-2 | 53.533 | 29.200 | -1.052 | 0.023 | 0.052 |
| Top2b | Q64511 | DNA topoisomerase 2-beta | 93.867 | 48.533 | -1.011 | < 0.001 | < 0.001 |
| Dnah3 | Q8BW94 | Dynein heavy chain 3, axonemal | 167.400 | 117.333 | -0.651 | 0.026 | 0.057 |
| Eppk1 | Q8R0W0 | Epiplakin | 70.933 | 51.067 | -0.448 | 0.042 | 0.083 |
| Dst | Q91ZU6 | Dystonin | 294.733 | 198.533 | -0.541 | 0.011 | 0.028 |
| Dnah8 | Q91XQ0 | Dynein heavy chain 8, axonemal | 179.600 | 117.200 | -0.636 | 0.001 | 0.004 |
| Mycbp2 | Q7TPH6 | E3 ubiquitin-protein ligase MYCBP2 | 88.400 | 52.133 | -0.737 | < 0.001 | 0.003 |
| Syne2 | Q6ZWQ0 | Nesprin-2 | 173.133 | 131.067 | -0.397 | 0.003 | 0.010 |
| Nin | Q61043 | Ninein | 115.600 | 69.467 | -0.724 | 0.003 | 0.009 |
| Fat2 | Q5F226 | Protocadherin Fat 2 | 28.600 | 19.933 | -0.596 | 0.005 | 0.016 |
| Xirp2 | Q4U4S6 | Xin actin-binding repeat-containing protein 2 | 108.200 | 63.800 | -0.908 | 0.024 | 0.054 |
| Top2a | Q01320 | DNA topoisomerase 2-alpha | 136.267 | 108.667 | -0.406 | 0.047 | 0.092 |
| Mcm8 | Q9CWV1 | DNA helicase MCM8 | 27.733 | 19.000 | -0.531 | 0.041 | 0.082 |
| Lrif1 | Q8CDD9 | Ligand-dependent nuclear receptor-interacting factor 1 | 68.267 | 44.867 | -0.782 | 0.035 | 0.071 |
| Myo9b | Q9QY06 | Unconventional myosin-IXb | 59.000 | 32.600 | -0.830 | 0.022 | 0.051 |
| Tspoap1 | Q7TNF8 | Peripheral-type benzodiazepine receptor-associated protein 1 | 43.400 | 24.200 | -0.893 | 0.004 | 0.013 |
| Ccdc150 | Q8CDI7 | Coiled-coil domain-containing protein 150 | 76.000 | 35.533 | -1.046 | < 0.001 | 0.001 |
| Itpr1 | P11881 | Inositol 1,4,5-trisphosphate receptor type 1 | 65.333 | 35.933 | -0.822 | 0.031 | 0.064 |
| Fam208b | Q5DTT3 | Protein FAM208B | 68.267 | 36.000 | -0.857 | 0.038 | 0.076 |
| Ranbp2 | Q9ERU9 | E3 SUMO-protein ligase RanBP2 | 107.533 | 49.933 | -1.106 | < 0.001 | 0.001 |
| Clmn | Q8C5W0 | Calmin | 71.000 | 29.733 | -0.971 | 0.012 | 0.030 |
| Herc2 | Q4U2R1 | E3 ubiquitin-protein ligase HERC2 | 109.867 | 83.333 | -0.363 | 0.048 | 0.092 |
| Atad5 | Q4QY64 | ATPase family AAA domain-containing protein 5 | 125.600 | 73.933 | -0.867 | 0.001 | 0.003 |
| Ep400 | Q8CHI8 | E1A-binding protein p400 | 72.600 | 43.733 | -1.359 | 0.009 | 0.024 |
| Cacna1c | Q01815 | Voltage-dependent L-type calcium channel subunit alpha-1C | 75.267 | 38.200 | -1.115 | 0.001 | 0.003 |
| Ifih1 | Q8R5F7 | Interferon-induced helicase C domain-containing protein 1 | 45.467 | 26.667 | -0.945 | 0.001 | 0.005 |
| Zcchc2 | Q69ZB8 | Zinc finger CCHC domain-containing protein 2 | 53.333 | 33.067 | -0.956 | 0.010 | 0.026 |
| Syce1 | Q9D495 | Synaptonemal complex central element protein 1 | 33.600 | 20.267 | -0.783 | < 0.001 | 0.003 |
| Kif21a | Q9QXL2 | Kinesin-like protein KIF21A | 85.800 | 63.267 | -0.520 | 0.039 | 0.078 |
| Col27a1 | Q5QNQ9 | Collagen alpha-1(XXVII) chain | 68.467 | 37.800 | -0.907 | 0.028 | 0.061 |
| Pla2g4c | Q64GA5 | Cytosolic phospholipase A2 gamma | 102.867 | 57.267 | -0.838 | 0.008 | 0.022 |
| Fam83f | Q3UKU4 | Protein FAM83F | 61.200 | 34.533 | -0.830 | 0.001 | 0.004 |
| Ptpn13 | Q64512 | Tyrosine-protein phosphatase non-receptor type 13 | 114.933 | 61.533 | -0.894 | 0.001 | 0.003 |
| Tiam2 | Q6ZPF3 | T-lymphoma invasion and metastasis-inducing protein 2 | 56.467 | 31.867 | -0.980 | 0.001 | 0.005 |
| Ccdc187 | Q8C5V8 | Coiled-coil domain-containing protein 187 | 74.867 | 40.067 | -1.211 | 0.001 | 0.005 |
| Cep70 | Q6IQY5 | Centrosomal protein of 70 kDa | 143.800 | 74.800 | -1.046 | < 0.001 | 0.002 |
| Wdr66 | E9Q743 | Cilia- and flagella-associated protein 251 | 87.067 | 48.667 | -0.872 | < 0.001 | 0.001 |
| Rab3gap2 | Q8BMG7 | Rab3 GTPase-activating protein non-catalytic subunit | 85.533 | 25.800 | -1.428 | 0.006 | 0.018 |
| Med12 | A2AGH6 | Mediator of RNA polymerase II transcription subunit 12 | 91.733 | 49.667 | -1.011 | 0.011 | 0.028 |
| Smarcc2 | Q6PDG5 | SWI/SNF complex subunit SMARCC2 | 53.933 | 28.133 | -1.335 | 0.001 | 0.005 |
| Sh3rf1 | Q69ZI1 | E3 ubiquitin-protein ligase SH3RF1 | 52.533 | 33.600 | -0.611 | 0.025 | 0.055 |
| Prkdc | P97313 | DNA-dependent protein kinase catalytic subunit | 252.267 | 142.067 | -0.764 | 0.001 | 0.005 |
| Myo6 | Q64331 | Unconventional myosin-VI | 53.400 | 38.667 | -0.504 | 0.023 | 0.052 |
| Washc5 | Q8C2E7 | WASH complex subunit 5 | 41.800 | 24.267 | -0.686 | 0.016 | 0.039 |
| Mast4 | Q811L6 | Microtubule-associated serine/threonine-protein kinase 4 | 179.267 | 131.200 | -0.954 | 0.049 | 0.094 |
| Lrrc9 | Q8CDN9 | Leucine-rich repeat-containing protein 9 | 58.467 | 39.000 | -0.639 | 0.004 | 0.012 |
| Patj | Q63ZW7 | InaD-like protein | 31.333 | 14.933 | -1.130 | 0.002 | 0.007 |
| Hnrnpm | Q9D0E1 | Heterogeneous nuclear ribonucleoprotein M | 55.600 | 34.733 | -1.015 | 0.012 | 0.030 |
| Ankrd11 | E9Q4F7 | Ankyrin repeat domain-containing protein 11 | 49.200 | 32.533 | -0.680 | 0.023 | 0.052 |
| Jbts17 | Q8CE72 | Protein JBTS17 | 69.867 | 40.933 | -0.956 | 0.005 | 0.017 |
| Gatad1 | Q920S3 | GATA zinc finger domain-containing protein 1 | 71.133 | 22.400 | -1.434 | < 0.001 | 0.002 |
| Drc7 | Q6V3W6 | Dynein regulatory complex subunit 7 | 70.067 | 32.933 | -1.159 | 0.001 | 0.003 |
| Uaca | Q8CGB3 | Uveal autoantigen with coiled-coil domains and ankyrin repeats | 185.933 | 84.867 | -1.380 | 0.039 | 0.078 |
| Col5a1 | O88207 | Collagen alpha-1(V) chain | 30.733 | 19.000 | -0.696 | 0.008 | 0.023 |
| Mon2 | Q80TL7 | Protein MON2 homolog | 50.800 | 27.667 | -0.990 | < 0.001 | 0.001 |
| Zmym2 | Q9CU65 | Zinc finger MYM-type protein 2 | 38.000 | 24.800 | -0.792 | 0.037 | 0.075 |
| Lrrc45 | Q8CIM1 | Leucine-rich repeat-containing protein 45 | 75.933 | 28.933 | -1.392 | < 0.001 | 0.002 |
| Gnpat | P98192 | Dihydroxyacetone phosphate acyltransferase | 66.267 | 36.533 | -0.986 | 0.002 | 0.008 |
| Cenpj | Q569L8 | Centromere protein J | 64.000 | 40.133 | -0.956 | 0.031 | 0.064 |
| Cyp2b9 | P12790 | Cytochrome P450 2B9 | 56.733 | 36.333 | -0.680 | 0.037 | 0.076 |
| Itih1 | Q61702 | Inter-alpha-trypsin inhibitor heavy chain H1 | 46.467 | 32.600 | -0.694 | 0.036 | 0.073 |
| Otof | Q9ESF1 | Otoferlin | 112.733 | 68.600 | -0.677 | 0.007 | 0.022 |
| Chd4 | Q6PDQ2 | Chromodomain-helicase-DNA-binding protein 4 | 156.400 | 87.733 | -1.140 | 0.002 | 0.009 |
| Col4a3 | Q9QZS0 | Collagen alpha-3(IV) chain | 126.867 | 46.733 | -1.525 | < 0.001 | < 0.001 |
| Ext2 | P70428 | Exostosin-2 | 44.667 | 27.467 | -0.802 | 0.003 | 0.011 |
| Acaca | Q5SWU9 | Acetyl-CoA carboxylase 1 | 71.267 | 43.333 | -0.616 | 0.026 | 0.058 |
| Pum3 | Q8BKS9 | Pumilio homolog 3 | 52.867 | 35.933 | -0.584 | 0.003 | 0.010 |
| Ankef1 | Q9D2J7 | Ankyrin repeat and EF-hand domain-containing protein 1 | 34.733 | 19.600 | -0.825 | 0.008 | 0.022 |
| Smg8 | Q8VE18 | Protein SMG8 | 34.800 | 26.533 | -0.467 | 0.043 | 0.084 |
| Safb | D3YXK2 | Scaffold attachment factor B1 | 53.467 | 26.400 | -1.113 | 0.003 | 0.010 |
| Spata6 | Q3U6K5 | Spermatogenesis-associated protein 6 | 55.667 | 25.133 | -1.364 | < 0.001 | 0.001 |
| Fat4 | Q2PZL6 | Protocadherin Fat 4 | 31.800 | 17.267 | -0.851 | < 0.001 | 0.003 |
| Ggt7 | Q99JP7 | Glutathione hydrolase 7 | 66.467 | 47.733 | -0.543 | 0.036 | 0.073 |
| Pola1 | P33609 | DNA polymerase alpha catalytic subunit | 36.400 | 23.267 | -1.004 | 0.004 | 0.012 |
| Nsf | P46460 | Vesicle-fusing ATPase | 62.000 | 35.800 | -1.054 | 0.008 | 0.022 |
| Tnrc18 | Q80WC3 | Trinucleotide repeat-containing gene 18 protein | 139.733 | 91.333 | -0.730 | 0.004 | 0.014 |
| Pde10a | Q8CA95 | cAMP and cAMP-inhibited cGMP 3',5'-cyclic phosphodiesterase 10A | 30.067 | 22.800 | -0.462 | 0.042 | 0.082 |
| Ssh1 | Q76I79 | Protein phosphatase Slingshot homolog 1 | 96.733 | 18.000 | -2.635 | < 0.001 | < 0.001 |
| Sel1l3 | Q80TS8 | Protein sel-1 homolog 3 | 57.533 | 33.933 | -1.254 | < 0.001 | 0.003 |
| Cebpz | P53569 | CCAAT/enhancer-binding protein zeta | 126.200 | 58.867 | -1.017 | < 0.001 | < 0.001 |
| Ly75 | Q60767 | Lymphocyte antigen 75 | 91.333 | 22.600 | -1.807 | < 0.001 | < 0.001 |
| Aspscr1 | Q8VBT9 | Tether containing UBX domain for GLUT4 | 50.400 | 20.533 | -1.428 | < 0.001 | < 0.001 |
| Parp1 | P11103 | Poly [ADP-ribose] polymerase 1 | 111.800 | 55.600 | -1.060 | < 0.001 | < 0.001 |
| Cdk5rap2 | Q8K389 | CDK5 regulatory subunit-associated protein 2 | 118.333 | 77.000 | -0.569 | 0.011 | 0.029 |
| Vps26b | Q8C0E2 | Vacuolar protein sorting-associated protein 26B | 82.867 | 40.400 | -1.115 | < 0.001 | 0.001 |
| Col7a1 | Q63870 | Collagen alpha-1(VII) chain | 58.000 | 39.600 | -0.740 | 0.029 | 0.061 |
| Foxred2 | Q3USW5 | FAD-dependent oxidoreductase domain-containing protein 2 | 97.333 | 40.133 | -1.306 | < 0.001 | < 0.001 |
| Mrp-l27 | Q99N92 | 39S ribosomal protein L27, mitochondrial | 112.867 | 15.000 | -2.930 | < 0.001 | < 0.001 |
| Rnf10 | Q3UIW5 | RING finger protein 10 | 28.067 | 18.867 | -0.675 | 0.002 | 0.008 |
| Ptprs | B0V2N1 | Receptor-type tyrosine-protein phosphatase S | 40.267 | 24.067 | -0.620 | 0.022 | 0.049 |
| Znf451 | Q8C0P7 | E3 SUMO-protein ligase ZNF451 | 87.333 | 42.600 | -0.957 | 0.007 | 0.019 |
| Tfpi | O54819 | Tissue factor pathway inhibitor | 83.733 | 44.333 | -1.033 | 0.008 | 0.023 |
| Ift88 | Q61371 | Intraflagellar transport protein 88 homolog | 42.333 | 26.000 | -0.769 | 0.019 | 0.045 |
| Stk11ip | Q3TAA7 | Serine/threonine-protein kinase 11-interacting protein | 32.800 | 17.933 | -0.909 | < 0.001 | 0.002 |
| Mybbp1a | Q7TPV4 | Myb-binding protein 1A | 34.333 | 9.667 | -1.831 | < 0.001 | < 0.001 |
| Trim68 | Q8K243 | E3 ubiquitin-protein ligase TRIM68 | 70.000 | 31.267 | -1.185 | < 0.001 | 0.001 |
| Rpgrip1l | Q8CG73 | Protein fantom | 50.600 | 24.867 | -1.071 | < 0.001 | 0.003 |
| MRCK alpha | Q3UU96 | Serine/threonine-protein kinase MRCK alpha | 201.000 | 82.867 | -1.302 | < 0.001 | < 0.001 |
| Bicc1 | Q99MQ1 | Protein bicaudal C homolog 1 | 96.800 | 69.133 | -0.678 | 0.047 | 0.091 |
| Sh2d3c | Q9QZS8 | SH2 domain-containing protein 3C | 58.533 | 45.400 | -0.337 | 0.020 | 0.046 |
| Cluap1 | Q8R3P7 | Clusterin-associated protein 1 | 43.600 | 30.400 | -0.546 | 0.039 | 0.078 |
| Myo5a | Q99104 | Unconventional myosin-Va | 68.733 | 35.467 | -0.978 | < 0.001 | < 0.001 |
| Gspt2 | Q149F3 | Eukaryotic peptide chain release factor GTP-binding subunit ERF3B | 69.267 | 26.000 | -1.504 | < 0.001 | < 0.001 |
| Ttc23l | A6H6E9 | Tetratricopeptide repeat protein 23-like | 45.800 | 25.200 | -0.887 | < 0.001 | < 0.001 |
| Wdfy3 | Q6VNB8 | WD repeat and FYVE domain-containing protein 3 | 94.067 | 64.333 | -0.694 | 0.023 | 0.052 |
| Ppp5c | Q60676 | Serine/threonine-protein phosphatase 5 | 92.133 | 46.000 | -1.154 | 0.005 | 0.016 |
| Zc3h18 | Q0P678 | Zinc finger CCCH domain-containing protein 18 | 89.400 | 43.733 | -1.098 | 0.009 | 0.024 |
| Antxr1 | Q9CZ52 | Anthrax toxin receptor 1 | 153.533 | 78.267 | -1.056 | < 0.001 | 0.002 |
| Adamts19 | P59509 | A disintegrin and metalloproteinase with thrombospondin motifs 19 | 97.867 | 42.667 | -1.245 | 0.008 | 0.022 |
| Galnt3 | P70419 | Polypeptide N-acetylgalactosaminyltransferase 3 | 32.667 | 15.333 | -0.907 | 0.004 | 0.014 |
| Dna2 | Q6ZQJ5 | DNA replication ATP-dependent helicase/nuclease DNA2 | 102.733 | 69.533 | -0.536 | 0.001 | 0.004 |
| Fig4 | Q91WF7 | Polyphosphoinositide phosphatase | 38.733 | 25.067 | -0.577 | 0.019 | 0.045 |
| Dph2 | Q9CR25 | 2-(3-amino-3-carboxypropyl)histidine synthase subunit 2 | 33.400 | 24.333 | -0.459 | 0.035 | 0.071 |
| Kif14 | L0N7N1 | Kinesin-like protein KIF14 | 51.467 | 26.200 | -0.981 | 0.007 | 0.022 |
| Iqcd | Q9D3V1 | Dynein regulatory complex protein 10 | 89.133 | 27.267 | -1.954 | < 0.001 | < 0.001 |
| Agbl3 | Q8CDP0 | Cytosolic carboxypeptidase 3 | 48.067 | 31.933 | -0.548 | 0.030 | 0.062 |
| Frmd3 | Q8BHD4 | FERM domain-containing protein 3 | 59.267 | 26.733 | -1.110 | < 0.001 | < 0.001 |
|  |  |  |  |  |  |  |  |
| UPREGULATED IN cNEB ko | | | | | | | |
| Myh1 | Q5SX40 | Myosin-1 | 1814.467 | 2801.733 | 0.666 | 0.001 | 0.004 |
| Myh7 | Q91Z83 | Myosin-7 | 165.400 | 906.400 | 3.470 | < 0.001 | 0.001 |
| Actn3 | O88990 | Alpha-actinin-3 | 356.933 | 723.733 | 0.922 | < 0.001 | 0.002 |
| Actn2 | Q9JI91 | Alpha-actinin-2 | 73.667 | 767.533 | 3.556 | < 0.001 | < 0.001 |
| Acta1 | P68134 | Actin, alpha skeletal muscle | 623.800 | 894.600 | 0.424 | 0.044 | 0.086 |
| Atp5f1b | P56480 | ATP synthase subunit beta, mitochondrial | 134.733 | 226.467 | 0.723 | 0.004 | 0.014 |
| Flnc | Q8VHX6 | Filamin-C | 75.400 | 310.533 | 1.691 | < 0.001 | < 0.001 |
| Atp5f1a | Q03265 | ATP synthase subunit alpha, mitochondrial | 322.733 | 632.667 | 0.950 | < 0.001 | < 0.001 |
| Ca3 | P16015 | Carbonic anhydrase 3 | 247.933 | 454.133 | 0.770 | 0.002 | 0.006 |
| Alb | P07724 | Serum albumin | 178.333 | 416.133 | 1.218 | < 0.001 | < 0.001 |
| Aco2 | Q99KI0 | Aconitate hydratase, mitochondrial | 210.333 | 328.600 | 0.584 | 0.006 | 0.018 |
| Myl1 | P05977 | Myosin light chain 1/3, skeletal muscle isoform | 416.133 | 547.267 | 0.388 | 0.015 | 0.035 |
| Ckmt2 | Q6P8J7 | Creatine kinase S-type, mitochondrial | 98.333 | 182.067 | 0.890 | < 0.001 | 0.003 |
| Fhl1 | P97447 | Four and a half LIM domains protein 1 | 73.533 | 352.533 | 2.217 | < 0.001 | < 0.001 |
| Ldb3 | Q9JKS4 | LIM domain-binding protein 3 | 60.267 | 213.000 | 1.842 | < 0.001 | < 0.001 |
| Got2 | P05202 | Aspartate aminotransferase, mitochondrial | 94.733 | 175.133 | 0.781 | 0.003 | 0.011 |
| Myoz1 | Q9JK37 | Myozenin-1 | 106.333 | 187.133 | 0.803 | < 0.001 | < 0.001 |
| Hsp90ab1 | P11499 | Heat shock protein HSP 90-beta | 82.333 | 163.733 | 1.001 | 0.002 | 0.009 |
| Vdac1 | Q60932 | Voltage-dependent anion-selective channel protein 1 | 44.000 | 63.333 | 0.568 | 0.016 | 0.039 |
| Cryab | P23927 | Alpha-crystallin B chain | 61.333 | 366.733 | 2.551 | < 0.001 | < 0.001 |
| Plec | Q9QXS1 | Plectin | 134.200 | 196.133 | 0.523 | 0.001 | 0.003 |
| Sdha | Q8K2B3 | Succinate dehydrogenase [ubiquinone] flavoprotein subunit, mitochondrial | 49.867 | 88.600 | 0.778 | 0.036 | 0.073 |
| Atp5o | Q9DB20 | ATP synthase subunit O, mitochondrial | 72.800 | 124.533 | 0.800 | 0.002 | 0.008 |
| Pdlim5 | Q8CI51 | PDZ and LIM domain protein 5 | 30.667 | 59.133 | 1.144 | < 0.001 | 0.001 |
| Bin1 | O08539 | Myc box-dependent-interacting protein 1 | 81.267 | 173.667 | 1.165 | < 0.001 | < 0.001 |
| Tuba4a | P68368 | Tubulin alpha-4A chain | 54.133 | 93.733 | 0.736 | 0.002 | 0.009 |
| Mdh1 | P14152 | Malate dehydrogenase, cytoplasmic | 29.800 | 46.467 | 0.592 | 0.017 | 0.040 |
| Hspd1 | P63038 | 60 kDa heat shock protein, mitochondrial | 88.067 | 116.333 | 0.408 | 0.007 | 0.019 |
| Pdlim3 | O70209 | PDZ and LIM domain protein 3 | 30.733 | 44.867 | 0.695 | 0.017 | 0.040 |
| Atp5h | Q9DCX2 | ATP synthase subunit d, mitochondrial | 14.400 | 31.467 | 1.094 | < 0.001 | < 0.001 |
| Hist1h1d | P43277 | Histone H1.3 | 20.467 | 35.867 | 0.950 | 0.002 | 0.008 |
| Atp5f1c | Q91VR2 | ATP synthase subunit gamma, mitochondrial | 28.133 | 57.533 | 0.974 | 0.009 | 0.024 |
| Cox4i1 | P19783 | Cytochrome c oxidase subunit 4 isoform 1, mitochondrial | 54.733 | 112.200 | 1.009 | < 0.001 | < 0.001 |
| Cyc1 | Q9D0M3 | Cytochrome c1, heme protein, mitochondrial | 31.133 | 54.867 | 0.779 | 0.001 | 0.005 |
| Hist1h2bp | Q8CGP2 | Histone H2B type 1-P | 24.933 | 124.400 | 2.294 | < 0.001 | < 0.001 |
| Atp5f1 | Q9CQQ7 | ATP synthase F(0) complex subunit B1, mitochondrial | 27.267 | 52.733 | 0.950 | 0.001 | 0.006 |
| Pdha1 | P35486 | Pyruvate dehydrogenase E1 component subunit alpha, somatic form, mitochondrial | 41.333 | 55.400 | 0.427 | 0.023 | 0.052 |
| Myoz3 | Q8R4E4 | Myozenin-3 | 24.067 | 64.933 | 1.377 | < 0.001 | < 0.001 |
| Rps27a | P62983 | Ubiquitin-40S ribosomal protein S27a | 59.667 | 177.200 | 1.317 | < 0.001 | < 0.001 |
| Hist1h4a | P62806 | Histone H4 | 20.267 | 281.000 | 2.674 | < 0.001 | < 0.001 |
| Mb | P04247 | Myoglobin | 53.533 | 134.933 | 1.094 | 0.001 | 0.003 |
| Cycs | P62897 | Cytochrome c, somatic | 22.400 | 47.800 | 0.805 | 0.024 | 0.054 |
| Slc25a3 | Q8VEM8 | Phosphate carrier protein, mitochondrial | 23.200 | 35.133 | 0.620 | 0.014 | 0.035 |
| Sdhb | Q9CQA3 | Succinate dehydrogenase [ubiquinone] iron-sulfur subunit, mitochondrial | 55.533 | 89.800 | 0.724 | 0.011 | 0.029 |
| Rps5 | P97461 | 40S ribosomal protein S5 | 25.733 | 60.267 | 1.111 | < 0.001 | < 0.001 |
| Pacsin3 | Q99JB8 | Protein kinase C and casein kinase II substrate protein 3 | 40.467 | 68.000 | 0.806 | 0.018 | 0.042 |
| Hist2h2be | Q64524 | Histone H2B type 2-E | 19.000 | 76.200 | 1.875 | < 0.001 | < 0.001 |
| Apobec2 | Q9WV35 | C->U-editing enzyme APOBEC-2 | 61.800 | 120.733 | 0.988 | < 0.001 | < 0.001 |
| Cox5a | P12787 | Cytochrome c oxidase subunit 5A, mitochondrial | 27.333 | 55.400 | 0.949 | < 0.001 | 0.001 |
| Dlst | Q9D2G2 | 2-Oxoglutarate Dehydrogenase Complex Component E2 | 40.467 | 115.600 | 1.563 | < 0.001 | < 0.001 |
| Rtn4 | Q99P72 | Reticulon-4 | 20.400 | 39.933 | 0.940 | < 0.001 | 0.001 |
| Rps8 | P62242 | 40S ribosomal protein S8 | 89.133 | 133.200 | 0.567 | 0.033 | 0.068 |
| Hsp90b1 | P08113 | Endoplasmin | 31.267 | 49.733 | 0.754 | 0.003 | 0.009 |
| Hist1h1a | P43275 | Histone H1.1 | 45.933 | 88.867 | 1.002 | 0.001 | 0.004 |
| Msn | P26041 | Moesin | 53.200 | 68.667 | 0.390 | 0.034 | 0.070 |
| Klk1b9 | P15949 | Kallikrein 1-related peptidase b9 | 24.800 | 34.933 | 0.551 | 0.019 | 0.044 |
| Lama2 | Q60675 | Laminin subunit alpha-2 | 50.400 | 77.133 | 0.883 | 0.008 | 0.022 |
| Sdhc | Q9CZB0 | Succinate dehydrogenase cytochrome b560 subunit, mitochondrial | 43.000 | 111.733 | 1.298 | < 0.001 | 0.001 |
| Nol8 | Q3UHX0 | Nucleolar protein 8 | 16.200 | 31.733 | 1.263 | < 0.001 | 0.003 |
| Rpl10l | P86048 | 60S ribosomal protein L10-like | 22.667 | 51.067 | 1.166 | < 0.001 | 0.002 |
| Rnf213 | E9Q555 | E3 ubiquitin-protein ligase RNF213 | 97.333 | 184.867 | 0.839 | < 0.001 | 0.003 |
| Dync2h1 | Q45VK7 | Cytoplasmic dynein 2 heavy chain 1 | 124.800 | 151.133 | 0.315 | 0.043 | 0.084 |
| Kiaa1210 | E9Q0C6 | Acrosomal protein KIAA1210 | 24.533 | 48.067 | 0.945 | 0.004 | 0.012 |
| Apc2 | Q9Z1K7 | Adenomatous polyposis coli protein 2 | 54.400 | 106.400 | 0.852 | 0.042 | 0.083 |
| Tbcd | Q8BYA0 | Tubulin-specific chaperone D | 35.400 | 53.133 | 0.561 | 0.010 | 0.026 |
| Cacna1e | Q61290 | Voltage-dependent R-type calcium channel subunit alpha-1E | 45.467 | 162.600 | 1.450 | 0.006 | 0.018 |
| Cfap54 | Q8C6S9 | Cilia- and flagella-associated protein 54 | 54.733 | 89.000 | 0.807 | 0.002 | 0.009 |
| Setbp1 | Q9Z180 | SET-binding protein | 23.267 | 55.800 | 1.130 | 0.001 | 0.004 |
| Polr2a | P08775 | DNA-directed RNA polymerase II subunit RPB1 | 33.133 | 48.533 | 0.465 | 0.007 | 0.021 |
| Kmt2a | P55200 | Histone-lysine N-methyltransferase 2A | 70.467 | 96.467 | 0.435 | 0.003 | 0.011 |
| Kif26b | Q7TNC6 | Kinesin-like protein KIF26B | 42.867 | 71.000 | 0.792 | 0.001 | 0.004 |
| Fyco1 | Q8VDC1 | FYVE and coiled-coil domain-containing protein 1 | 28.067 | 67.733 | 1.267 | < 0.001 | 0.001 |
| Srrm1 | Q52KI8 | Serine/arginine repetitive matrix protein 1 | 37.600 | 88.267 | 1.495 | < 0.001 | 0.002 |
| Cdh23 | Q99PF4 | Cadherin-23 | 27.733 | 45.267 | 0.718 | 0.007 | 0.022 |
| Ank2 | Q8C8R3 | Ankyrin-2 | 19.133 | 31.867 | 0.774 | 0.008 | 0.022 |
| Flg2 | Q2VIS4 | Filaggrin-2 | 52.800 | 92.000 | 0.845 | 0.011 | 0.029 |
| Armc4 | B2RY50 | Armadillo repeat-containing protein 4 | 27.133 | 61.133 | 1.026 | 0.003 | 0.011 |
| Casp8ap2 | Q9WUF3 | CASP8-associated protein 2 | 48.133 | 102.200 | 1.181 | 0.001 | 0.005 |
| Lrp2 | A2ARV4 | Low-density lipoprotein receptor-related protein 2 | 49.333 | 157.467 | 1.833 | < 0.001 | 0.001 |
| Prkce | P16054 | Protein kinase C epsilon type | 35.867 | 59.667 | 0.918 | 0.001 | 0.005 |
| Rb1cc1 | Q9ESK9 | RB1-inducible coiled-coil protein 1 | 168.667 | 271.333 | 0.846 | 0.028 | 0.060 |
| Asap3 | Q5U464 | Arf-GAP with SH3 domain, ANK repeat and PH domain-containing protein 3 | 31.867 | 41.133 | 0.370 | 0.011 | 0.029 |
| Kntc1 | Q8C3Y4 | Kinetochore-associated protein 1 | 35.733 | 50.000 | 0.513 | 0.038 | 0.076 |
| Dopey2 | Q3UHQ6 | Protein dopey-2 | 38.400 | 62.400 | 0.687 | 0.004 | 0.012 |
| Brwd1 | Q921C3 | Bromodomain and WD repeat-containing protein 1 | 53.800 | 86.533 | 0.725 | 0.003 | 0.010 |
| SR-Beta | P47758 | Signal recognition particle receptor subunit beta | 13.200 | 31.533 | 1.288 | < 0.001 | < 0.001 |
| Znf518a | B2RRF6 | Zinc finger protein 518A | 65.333 | 132.000 | 1.202 | < 0.001 | 0.001 |
| Aspm | Q8CJ27 | Abnormal spindle-like microcephaly-associated protein homolog | 118.533 | 171.933 | 0.507 | 0.001 | 0.004 |
| Ubr1 | O70481 | E3 ubiquitin-protein ligase UBR1 | 14.200 | 33.933 | 0.905 | 0.004 | 0.013 |
| Atrx | Q61687 | Transcriptional regulator ATRX | 64.600 | 140.600 | 0.964 | 0.001 | 0.005 |
| Chst3 | O88199 | Carbohydrate sulfotransferase 3 | 46.333 | 79.867 | 0.777 | < 0.001 | < 0.001 |
| Trappc12 | Q8K2L8 | Trafficking protein particle complex subunit 12 | 34.600 | 65.667 | 0.914 | 0.003 | 0.011 |
| Gtf2ird2 | Q99NI3 | General transcription factor II-I repeat domain-containing protein 2 | 54.933 | 88.333 | 0.748 | 0.012 | 0.031 |
| Crebbp | P45481 | CREB-binding protein | 42.400 | 58.467 | 0.574 | 0.010 | 0.026 |
| Myt1l | P97500 | Myelin transcription factor 1-like proldb3tein | 110.467 | 149.667 | 0.546 | 0.017 | 0.041 |
| Rbm28 | Q8CGC6 | RNA-binding protein 28 | 60.667 | 106.467 | 1.152 | 0.029 | 0.061 |
| #VALUE! | Q3USZ8 | Deleted in autism protein 1 homolog | 57.667 | 73.600 | 0.386 | 0.020 | 0.047 |
| Ptchd3 | Q0EEE2 | Patched domain-containing protein 3 | 53.933 | 72.667 | 0.478 | 0.021 | 0.049 |
| Catsperg2 | C6KI89 | Cation channel sperm-associated protein subunit gamma 2 | 38.067 | 86.600 | 1.061 | 0.001 | 0.003 |
| Dennd5b | A2RSQ0 | DENN domain-containing protein 5B | 38.800 | 65.200 | 0.560 | 0.027 | 0.058 |
| Kat6a | Q8BZ21 | Histone acetyltransferase KAT6A | 67.533 | 92.133 | 0.396 | 0.028 | 0.061 |
| Cep170b | Q80U49 | Centrosomal protein of 170 kDa protein B | 51.933 | 73.333 | 0.913 | 0.044 | 0.086 |
| Tex15 | F8VPN2 | Testis-expressed protein 15 | 123.000 | 255.867 | 1.042 | < 0.001 | < 0.001 |
| Plk1 | Q07832 | Serine/threonine-protein kinase PLK1 | 38.533 | 57.000 | 0.531 | 0.002 | 0.008 |
| Pnpt1 | Q8K1R3 | Polyribonucleotide nucleotidyltransferase 1, mitochondrial | 67.067 | 108.933 | 1.147 | 0.006 | 0.018 |
| Dcaf12 | Q8BGZ3 | DDB1- and CUL4-associated factor 12 | 21.933 | 44.267 | 0.899 | 0.006 | 0.017 |
| Oas1b | Q60856 | Inactive 2'-5'-oligoadenylate synthase 1B | 24.667 | 35.867 | 0.586 | 0.008 | 0.023 |
| Gstcd | Q5RL51 | Glutathione S-transferase C-terminal domain-containing protein | 24.267 | 43.667 | 0.660 | 0.024 | 0.054 |
| Kdm7a | Q3UWM4 | Lysine-specific demethylase 7A | 27.667 | 76.267 | 1.346 | < 0.001 | 0.001 |
| Ush1c | Q9ES64 | Harmonin | 30.467 | 73.333 | 1.179 | < 0.001 | 0.001 |
| Vegfr2 | P35918 | Vascular endothelial growth factor receptor 2 | 14.733 | 41.467 | 1.513 | < 0.001 | < 0.001 |
|  |  |  |  |  |  |  |  |
| NO SIGNIFICANT DIFFERENCES BETWEEN WT AND cNEB ko | | | | | | | |
| Ttn | A2ASS6 | Titin | 1230.533 | 1664.000 | 0.248 | 0.199 | 0.303 |
| Atp2a1 | Q8R429 | Sarcoplasmic/endoplasmic reticulum calcium ATPase 1 | 1188.467 | 1067.533 | -0.182 | 0.387 | 0.511 |
| Myh3 | P13541 | Myosin-3 | 84.533 | 91.467 | 0.187 | 0.652 | 0.761 |
| Pygm | Q9WUB3 | Glycogen phosphorylase, muscle form | 806.133 | 695.133 | -0.233 | 0.228 | 0.333 |
| Tpm2 | P58774 | Tropomyosin beta chain | 561.067 | 507.333 | -0.075 | 0.758 | 0.836 |
| Mdh2 | P08249 | Malate dehydrogenase, mitochondrial | 174.800 | 232.667 | 0.411 | 0.067 | 0.123 |
| Tpi1 | P17751 | Triosephosphate isomerase | 98.667 | 82.667 | -0.365 | 0.200 | 0.302 |
| Srl | Q7TQ48 | Sarcalumenin | 136.933 | 190.067 | 0.511 | 0.094 | 0.158 |
| Hspa8 | P63017 | Heat shock cognate 71 kDa protein | 68.533 | 112.600 | 0.612 | 0.060 | 0.112 |
| Slc25a4 | P48962 | ADP/ATP translocase 1 | 235.200 | 295.133 | 0.291 | 0.070 | 0.126 |
| Cs | Q9CZU6 | Citrate synthase, mitochondrial | 165.933 | 228.400 | 0.468 | 0.052 | 0.098 |
| Adssl1 | P28650 | Adenylosuccinate synthetase isozyme 1 | 59.267 | 65.733 | 0.167 | 0.589 | 0.703 |
| Ndufs1 | Q91VD9 | NADH-ubiquinone oxidoreductase 75 kDa subunit, mitochondrial | 96.600 | 123.000 | 0.241 | 0.411 | 0.530 |
| Got1 | P05201 | Aspartate aminotransferase, cytoplasmic | 35.667 | 46.800 | 0.198 | 0.530 | 0.645 |
| Ryr1 | E9PZQ0 | Ryanodine receptor 1 | 122.933 | 121.600 | -0.081 | 0.685 | 0.786 |
| Anxa6 | P14824 | Annexin A6 | 36.200 | 45.467 | 0.344 | 0.269 | 0.379 |
| Hspa5 | P20029 | Endoplasmic reticulum chaperone BiP | 36.267 | 40.133 | 0.116 | 0.717 | 0.811 |
| Eef1a2 | P62631 | Elongation factor 1-alpha 2 | 224.933 | 217.133 | -0.071 | 0.642 | 0.756 |
| Ugp2 | Q91ZJ5 | UTP--glucose-1-phosphate uridylyltransferase | 57.667 | 78.867 | 0.398 | 0.136 | 0.222 |
| Myom1 | Q62234 | Myomesin-1 | 58.533 | 68.200 | 0.169 | 0.464 | 0.586 |
| Tnni2 | P13412 | Troponin I, fast skeletal muscle | 173.467 | 143.133 | -0.326 | 0.107 | 0.180 |
| Etfb | Q9DCW4 | Electron transfer flavoprotein subunit beta | 59.600 | 92.400 | 0.545 | 0.168 | 0.265 |
| Gpi | P06745 | Glucose-6-phosphate isomerase | 98.600 | 84.800 | -0.307 | 0.209 | 0.312 |
| Rtn2 | O70622 | Reticulon-2 | 62.800 | 55.600 | -0.204 | 0.526 | 0.643 |
| Pvalb | P32848 | Parvalbumin alpha | 162.200 | 120.400 | -0.463 | 0.116 | 0.193 |
| Atp1a2 | Q6PIE5 | Sodium/potassium-transporting ATPase subunit alpha-2 | 41.000 | 69.867 | 0.727 | 0.089 | 0.151 |
| Krt5 | Q922U2 | Keratin, type II cytoskeletal 5 | 136.200 | 149.933 | 0.209 | 0.360 | 0.481 |
| Dld | O08749 | Dihydrolipoyl dehydrogenase, mitochondrial | 111.333 | 139.200 | 0.328 | 0.148 | 0.236 |
| Fh | P97807 | Fumarate hydratase, mitochondrial | 23.133 | 29.933 | 0.375 | 0.153 | 0.244 |
| Obscn | A2AAJ9 | Obscurin | 161.733 | 186.133 | 0.346 | 0.381 | 0.507 |
| Eci1 | P42125 | Enoyl-CoA delta isomerase 1, mitochondrial | 68.467 | 80.933 | 0.012 | 0.978 | 0.978 |
| Immt | Q8CAQ8 | MICOS complex subunit Mic60 | 31.267 | 25.400 | -0.154 | 0.667 | 0.772 |
| Acat1 | Q8QZT1 | Acetyl-CoA acetyltransferase, mitochondrial | 49.400 | 51.600 | 0.199 | 0.557 | 0.674 |
| Sypl2 | O89104 | Synaptophysin-like protein 2 | 35.000 | 29.867 | -0.367 | 0.238 | 0.344 |
| Uqcrb | Q9D855 | Cytochrome b-c1 complex subunit 7 | 85.200 | 136.533 | 0.750 | 0.053 | 0.100 |
| Nipsnap2 | O55126 | Protein NipSnap homolog 2 | 51.267 | 82.800 | 0.488 | 0.229 | 0.334 |
| Decr1 | Q9CQ62 | 2,4-dienoyl-CoA reductase, mitochondrial | 62.467 | 83.400 | 0.416 | 0.212 | 0.314 |
| Hspg2 | Q05793 | Basement membrane-specific heparan sulfate proteoglycan core protein | 28.667 | 23.867 | -0.155 | 0.410 | 0.532 |
| Sod2 | P09671 | Superoxide dismutase [Mn], mitochondrial | 33.133 | 43.533 | 0.353 | 0.261 | 0.373 |
| Tnni1 | Q9WUZ5 | Troponin I, slow skeletal muscle | 68.733 | 59.200 | -0.131 | 0.600 | 0.713 |
| Mtco2 | P00405 | Cytochrome c oxidase subunit 2 | 35.600 | 35.000 | -0.043 | 0.912 | 0.933 |
| Gyg1 | Q9R062 | Glycogenin-1 | 29.600 | 35.467 | 0.124 | 0.662 | 0.769 |
| P4hb | P09103 | Protein disulfide-isomerase | 68.867 | 56.733 | -0.259 | 0.479 | 0.600 |
| Park7 | Q99LX0 | Protein/nucleic acid deglycase DJ-1 | 64.667 | 87.400 | 0.351 | 0.209 | 0.312 |
| Krt42 | Q6IFX2 | Keratin, type I cytoskeletal 42 | 68.867 | 50.200 | -0.191 | 0.650 | 0.764 |
| Hist1h1b | P43276 | Histone H1.5 | 38.133 | 28.000 | -0.279 | 0.262 | 0.374 |
| Krt79 | Q8VED5 | Keratin, type II cytoskeletal 79 | 28.867 | 25.667 | 0.030 | 0.922 | 0.942 |
| Tmprss13 | Q5U405 | Transmembrane protease serine 13 | 124.533 | 171.000 | 0.531 | 0.133 | 0.218 |
| Ryr2 | E9Q401 | Ryanodine receptor 2 | 152.333 | 133.267 | -0.405 | 0.169 | 0.266 |
| Myh9 | Q8VDD5 | Myosin-9 | 86.400 | 90.000 | 0.159 | 0.596 | 0.709 |
| Snta1 | Q61234 | Alpha-1-syntrophin | 21.800 | 22.000 | -0.020 | 0.927 | 0.944 |
| Ryr3 | A2AGL3 | Ryanodine receptor 3 | 95.133 | 102.200 | 0.162 | 0.759 | 0.836 |
| Ccdc88a | Q5SNZ0 | Girdin | 99.800 | 117.000 | 0.089 | 0.821 | 0.877 |
| Myh10 | Q61879 | Myosin-10 | 24.933 | 22.933 | -0.173 | 0.459 | 0.582 |
| Fsip2 | A2ARZ3 | Fibrous sheath-interacting protein 2 | 246.667 | 251.200 | -0.005 | 0.979 | 0.977 |
| Zfp292 | Q9Z2U2 | Zinc finger protein 292 | 78.333 | 65.600 | -0.354 | 0.405 | 0.526 |
| Cenpe | Q6RT24 | Centromere-associated protein E | 60.133 | 48.067 | -0.494 | 0.147 | 0.236 |
| Nipbl | Q6KCD5 | Nipped-B-like protein | 81.667 | 95.467 | 0.120 | 0.625 | 0.741 |
| Cep290 | Q6A078 | Centrosomal protein of 290 kDa | 145.267 | 159.600 | 0.086 | 0.700 | 0.795 |
| Syne1 | Q6ZWR6 | Nesprin-1 | 265.733 | 298.267 | 0.170 | 0.304 | 0.414 |
| Macf1 | Q9QXZ0 | Microtubule-actin cross-linking factor 1 | 289.733 | 248.200 | -0.340 | 0.140 | 0.227 |
| Myo9a | Q8C170 | Unconventional myosin-IXa | 135.333 | 90.133 | -0.723 | 0.079 | 0.140 |
| Unc13c | Q8K0T7 | Protein unc-13 homolog C | 55.200 | 40.933 | -0.520 | 0.185 | 0.284 |
| Wdr19 | Q3UGF1 | WD repeat-containing protein 19 | 64.133 | 49.533 | -0.463 | 0.163 | 0.259 |
| Lrp1b | Q9JI18 | Low-density lipoprotein receptor-related protein 1B | 48.533 | 39.067 | -0.281 | 0.340 | 0.459 |
| Tbc1d12 | Q6A039 | TBC1 domain family member 12 | 42.800 | 30.400 | -0.430 | 0.091 | 0.154 |
| Ice1 | E9Q286 | Little elongation complex subunit 1 | 61.667 | 81.200 | 0.558 | 0.057 | 0.108 |
| Usp34 | Q6ZQ93 | Ubiquitin carboxyl-terminal hydrolase 34 | 38.800 | 59.400 | 0.563 | 0.106 | 0.178 |
| Znf638 | Q61464 | Zinc finger protein 638 | 56.933 | 62.800 | 0.049 | 0.887 | 0.921 |
| Ash1l | Q99MY8 | Histone-lysine N-methyltransferase ASH1L | 207.267 | 191.333 | -0.282 | 0.282 | 0.394 |
| Asxl2 | Q8BZ32 | Putative Polycomb group protein ASXL2 | 55.333 | 53.600 | 0.165 | 0.570 | 0.685 |
| Col6a2 | Q02788 | Collagen alpha-2(VI) chain | 96.000 | 73.467 | -0.421 | 0.127 | 0.209 |
| Zranb3 | Q6NZP1 | DNA annealing helicase and endonuclease ZRANB3 | 76.600 | 80.200 | 0.057 | 0.850 | 0.897 |
| Dnah5 | Q8VHE6 | Dynein heavy chain 5, axonemal | 72.400 | 74.667 | -0.194 | 0.654 | 0.761 |
| Dync1h1 | Q9JHU4 | Cytoplasmic dynein 1 heavy chain 1 | 152.200 | 123.400 | -0.317 | 0.116 | 0.194 |
| Rrbp1 | Q99PL5 | Ribosome-binding protein 1 | 47.600 | 43.067 | -0.122 | 0.737 | 0.815 |
| Chd2 | E9PZM4 | Chromodomain-helicase-DNA-binding protein 2 | 54.933 | 70.533 | 0.581 | 0.254 | 0.364 |
| Magi3 | Q9EQJ9 | Membrane-Associated Guanylate Kinase-Related 3 | 146.133 | 169.067 | 0.092 | 0.683 | 0.784 |
| Adgb | G3UZ78 | Androglobin | 135.867 | 90.733 | -0.667 | 0.059 | 0.111 |
| Mia3 | Q8BI84 | Transport and Golgi organization protein 1 homolog | 34.267 | 33.333 | 0.084 | 0.772 | 0.842 |
| Tln1 | P26039 | Talin-1 | 78.733 | 71.667 | -0.294 | 0.404 | 0.527 |
| Fbf1 | A2A870 | Fas-binding factor 1 | 73.467 | 53.467 | -0.394 | 0.084 | 0.145 |
| F8 | Q06194 | Coagulation factor VIII | 32.533 | 27.400 | -0.386 | 0.143 | 0.230 |
| Hsd17b4 | P51660 | Peroxisomal multifunctional enzyme type 2 | 55.667 | 40.067 | -0.304 | 0.434 | 0.553 |
| Pclo | Q9QYX7 | Protein piccolo | 221.267 | 138.667 | -0.730 | 0.052 | 0.099 |
| Setx | A2AKX3 | Probable helicase senataxin | 51.200 | 62.933 | 0.328 | 0.416 | 0.535 |
| Pdcd11 | Q6NS46 | Protein RRP5 homolog | 85.533 | 104.067 | 0.193 | 0.288 | 0.398 |
| Cit | P49025 | Citron Rho-interacting kinase | 83.067 | 91.867 | 0.148 | 0.353 | 0.473 |
| Gcn1 | E9PVA8 | eIF-2-alpha kinase activator GCN1 | 62.333 | 78.600 | 0.230 | 0.305 | 0.415 |
| Gcc2 | Q8CHG3 | GRIP and coiled-coil domain-containing protein 2 | 121.933 | 80.400 | -0.875 | 0.072 | 0.130 |
| Nos1 | Q9Z0J4 | Nitric oxide synthase, brain | 95.933 | 85.133 | -0.213 | 0.215 | 0.317 |
| Myof | Q69ZN7 | Myoferlin | 22.267 | 23.333 | 0.191 | 0.452 | 0.574 |
| Mical3 | Q8CJ19 | [F-actin]-monooxygenase MICAL3 | 84.400 | 77.200 | -0.011 | 0.959 | 0.960 |
| Fat3 | Q8BNA6 | Protocadherin Fat 3 | 66.933 | 54.467 | -0.275 | 0.181 | 0.280 |
| Evpl | Q9D952 | Envoplakin | 72.733 | 57.467 | -0.569 | 0.166 | 0.262 |
| Top3b | Q9Z321 | DNA topoisomerase 3-beta-1 | 48.133 | 32.800 | -0.496 | 0.097 | 0.163 |
| Cep350 | E9Q309 | Centrosome-associated protein 350 | 132.467 | 141.133 | 0.263 | 0.348 | 0.467 |
| Smc6 | Q924W5 | Structural maintenance of chromosomes protein 6 | 88.000 | 69.333 | -0.349 | 0.287 | 0.397 |
| Sptan1 | P16546 | Spectrin alpha chain, non-erythrocytic 1 | 101.000 | 88.467 | -0.312 | 0.181 | 0.279 |
| Rasgrf2 | P70392 | Ras-specific guanine nucleotide-releasing factor 2 | 32.267 | 38.067 | 0.415 | 0.144 | 0.232 |
| Heatr5a | Q5PRF0 | HEAT repeat-containing protein 5A | 35.000 | 21.000 | -0.573 | 0.079 | 0.139 |
| Cd4 | P06332 | T-cell surface glycoprotein CD4 | 53.133 | 45.667 | -0.258 | 0.281 | 0.393 |
| Cep162 | Q6ZQ06 | Centrosomal protein of 162 kDa | 61.667 | 62.867 | 0.205 | 0.687 | 0.786 |
| Cttnbp2 | B9EJA2 | Cortactin-binding protein 2 | 72.000 | 72.933 | -0.042 | 0.900 | 0.926 |
| Bod1l | E9Q6J5 | Biorientation of chromosomes in cell division protein 1-like 1 | 79.067 | 105.067 | 0.403 | 0.102 | 0.171 |
| Cacna1a | P97445 | Voltage-dependent P/Q-type calcium channel subunit alpha-1A | 63.533 | 69.133 | 0.422 | 0.265 | 0.374 |
| Dhx29 | Q6PGC1 | ATP-dependent RNA helicase DHX29 | 94.200 | 103.067 | -0.396 | 0.387 | 0.511 |
| Filip1 | Q9CS72 | Filamin-A-interacting protein 1 | 65.333 | 113.333 | 0.540 | 0.077 | 0.138 |
| Trip12 | G5E870 | E3 ubiquitin-protein ligase TRIP12 | 81.667 | 72.600 | -0.087 | 0.755 | 0.836 |
| Usp24 | B1AY13 | Ubiquitin carboxyl-terminal hydrolase 24 | 41.800 | 35.467 | -0.053 | 0.892 | 0.922 |
| Dmd | P11531 | Dystrophin | 111.000 | 98.667 | -0.201 | 0.303 | 0.413 |
| Pcnt | P48725 | Pericentrin | 82.467 | 70.867 | -0.284 | 0.306 | 0.414 |
| Dnah2 | P0C6F1 | Dynein heavy chain 2, axonemal | 90.067 | 103.733 | -0.032 | 0.944 | 0.955 |
| Specc1 | Q5SXY1 | Cytospin-B | 33.133 | 32.133 | -0.167 | 0.557 | 0.671 |
| Brca2 | P97929 | Breast cancer type 2 susceptibility protein homolog | 143.067 | 83.067 | -0.677 | 0.120 | 0.198 |
| Ppfia2 | Q8BSS9 | Liprin-alpha-2 | 56.400 | 39.000 | 0.188 | 0.726 | 0.814 |
| Pbrm1 | Q8BSQ9 | Protein polybromo-1 | 65.533 | 64.867 | 0.220 | 0.431 | 0.551 |
| Rbbp6 | P97868 | E3 ubiquitin-protein ligase RBBP6 | 67.133 | 71.067 | -0.155 | 0.650 | 0.763 |
| Kalrn | A2CG49 | Kalirin | 54.533 | 37.133 | -0.548 | 0.129 | 0.213 |
| Shroom3 | Q9QXN0 | Protein Shroom3 | 86.733 | 81.267 | -0.051 | 0.765 | 0.837 |
| Ccdc18 | Q640L5 | Coiled-coil domain-containing protein 18 | 59.467 | 60.000 | -0.149 | 0.760 | 0.836 |
| Jmjd1c | Q69ZK6 | Probable JmjC domain-containing histone demethylation protein 2C | 33.667 | 48.133 | 0.177 | 0.667 | 0.770 |
| Pzp | Q61838 | Pregnancy zone protein | 53.933 | 60.467 | 0.152 | 0.688 | 0.785 |
| Unc80 | Q8BLN6 | Protein unc-80 homolog | 78.467 | 92.400 | 0.410 | 0.191 | 0.292 |
| Knl1 | Q66JQ7 | Kinetochore scaffold 1 | 86.733 | 79.200 | -0.157 | 0.425 | 0.545 |
| Vav2 | Q60992 | Guanine nucleotide exchange factor VAV2 | 66.133 | 60.200 | -0.190 | 0.557 | 0.672 |
| Cep250 | Q60952 | Centrosome-associated protein CEP250 | 96.267 | 109.000 | -0.051 | 0.882 | 0.918 |
| Speg | Q62407 | Striated muscle-specific serine/threonine-protein kinase | 40.400 | 64.667 | 0.180 | 0.704 | 0.799 |
| Kcnh3 | Q9WVJ0 | Potassium voltage-gated channel subfamily H member 3 | 35.200 | 40.333 | 0.258 | 0.200 | 0.302 |
| Chd6 | A3KFM7 | Chromodomain-helicase-DNA-binding protein 6 | 145.400 | 169.000 | 0.088 | 0.652 | 0.762 |
| Tnks1bp1 | P58871 | 182 kDa tankyrase-1-binding protein | 62.533 | 79.733 | 0.476 | 0.373 | 0.497 |
| Ktn1 | Q61595 | Kinectin | 36.867 | 45.600 | 0.188 | 0.480 | 0.600 |
| Dapk1 | Q80YE7 | Death-associated protein kinase 1 | 88.133 | 84.600 | -0.051 | 0.692 | 0.790 |
| Lrrc4b | P0C192 | Leucine-rich repeat-containing protein 4B | 71.400 | 59.133 | -0.369 | 0.301 | 0.413 |
| H2afy | Q9QZQ8 | Core histone macro-H2A.1 | 43.733 | 54.000 | 0.488 | 0.094 | 0.158 |
| Nf1 | Q04690 | Neurofibromin | 101.933 | 73.000 | -0.139 | 0.731 | 0.816 |
| Svil | Q8K4L3 | Supervillin | 61.333 | 48.800 | -0.314 | 0.461 | 0.584 |
| Slf1 | Q8R3P9 | SMC5-SMC6 complex localization factor protein 1 | 55.800 | 87.333 | 0.465 | 0.326 | 0.440 |
| Slf2 | Q6P9P0 | SMC5-SMC6 complex localization factor protein 2 | 91.533 | 74.467 | -0.246 | 0.156 | 0.248 |
| Sel1l2 | Q3V172 | Protein sel-1 homolog 2 | 28.267 | 20.667 | -0.418 | 0.200 | 0.303 |
| Vps13c | Q8BX70 | Vacuolar protein sorting-associated protein 13C | 45.200 | 42.600 | 0.026 | 0.956 | 0.958 |
| Drc1 | Q3USS3 | Dynein regulatory complex protein 1 | 26.867 | 24.533 | 0.048 | 0.887 | 0.920 |
| Thoc2 | B1AZI6 | THO complex subunit 2 | 75.600 | 101.067 | 0.438 | 0.059 | 0.110 |
| Efcab5 | A0JP43 | EF-hand calcium-binding domain-containing protein 5 | 40.867 | 24.867 | -0.543 | 0.131 | 0.215 |
| Man1a2 | P39098 | Mannosyl-oligosaccharide 1,2-alpha-mannosidase IB | 84.267 | 69.267 | -0.325 | 0.241 | 0.348 |
| Kif21b | Q9QXL1 | Kinesin-like protein KIF21B | 75.133 | 63.800 | -0.544 | 0.186 | 0.284 |
| Akap9 | Q70FJ1 | A-kinase anchor protein 9 | 104.933 | 154.933 | 0.100 | 0.839 | 0.893 |
| Adamts15 | P59384 | A disintegrin and metalloproteinase with thrombospondin motifs 15 | 80.200 | 126.000 | 0.543 | 0.086 | 0.147 |
| Znf318 | Q99PP2 | Zinc finger protein 318 | 110.000 | 125.933 | 0.214 | 0.298 | 0.410 |
| Smg1 | Q8BKX6 | Serine/threonine-protein kinase SMG1 | 72.200 | 98.933 | 0.120 | 0.731 | 0.814 |
| Chd9 | Q8BYH8 | Chromodomain-helicase-DNA-binding protein 9 | 109.267 | 105.533 | 0.295 | 0.484 | 0.604 |
| Spta1 | P08032 | Spectrin alpha chain, erythrocytic 1 | 68.467 | 107.467 | 0.560 | 0.268 | 0.379 |
| Rev3l | Q61493 | DNA polymerase zeta catalytic subunit | 42.600 | 47.000 | 0.250 | 0.466 | 0.587 |
| Psmd6 | Q99JI4 | 26S proteasome non-ATPase regulatory subunit 6 | 42.200 | 33.667 | -0.478 | 0.062 | 0.117 |
| Aff4 | Q9ESC8 | AF4/FMR2 family member 4 | 34.333 | 45.467 | 0.254 | 0.429 | 0.550 |
| Sptbn1 | Q62261 | Spectrin beta chain, non-erythrocytic 1 | 98.800 | 78.733 | -0.165 | 0.786 | 0.853 |
| Col2a1 | P28481 | Collagen alpha-1(II) chain | 29.000 | 32.000 | 0.044 | 0.860 | 0.903 |
| Topaz1 | E5FYH1 | Protein TOPAZ1 | 75.667 | 63.533 | -0.199 | 0.730 | 0.817 |
| Bahcc1 | Q3UHR0 | BAH and coiled-coil domain-containing protein 1 | 68.733 | 64.333 | 0.024 | 0.931 | 0.945 |
| Fgd6 | Q69ZL1 | FYVE, RhoGEF and PH domain-containing protein 6 | 64.067 | 87.733 | 0.112 | 0.797 | 0.860 |
| Plekha7 | Q3UIL6 | Pleckstrin homology domain-containing family A member 7 | 46.000 | 60.267 | 0.333 | 0.377 | 0.501 |
| Actl7b | Q9QY83 | Actin-like protein 7B | 25.600 | 23.733 | -0.118 | 0.678 | 0.781 |
| Pi4ka | E9Q3L2 | Phosphatidylinositol 4-kinase alpha | 69.667 | 58.533 | 0.087 | 0.849 | 0.898 |
| Hydin | Q80W93 | Hydrocephalus-inducing protein | 88.600 | 65.400 | -0.190 | 0.681 | 0.783 |
| Fasn | P19096 | Fatty acid synthase | 39.200 | 52.867 | 0.118 | 0.731 | 0.814 |
| Ftsj3 | Q9DBE9 | pre-rRNA processing protein FTSJ3 | 20.533 | 24.933 | 0.342 | 0.173 | 0.269 |
| Plxna4 | Q80UG2 | Plexin-A4 | 41.333 | 44.200 | -0.152 | 0.657 | 0.763 |
| Usp9x | P70398 | Probable ubiquitin carboxyl-terminal hydrolase FAF-X | 136.400 | 103.267 | -0.379 | 0.264 | 0.375 |
| Dis3 | Q9CSH3 | Exosome complex exonuclease RRP44 | 56.867 | 70.133 | 0.265 | 0.397 | 0.520 |
| Trio | Q0KL02 | Triple functional domain protein | 32.600 | 29.867 | -0.164 | 0.490 | 0.609 |
| Znfx1 | Q8R151 | NFX1-type zinc finger-containing protein 1 | 47.000 | 32.333 | -0.834 | 0.068 | 0.125 |
| Cic | Q924A2 | Protein capicua homolog | 202.333 | 168.600 | -0.384 | 0.110 | 0.184 |
| Rusc1 | Q8BG26 | RUN and SH3 domain-containing protein 1 | 41.000 | 34.533 | -0.291 | 0.064 | 0.120 |
| Kif4 | P33174 | Chromosome-associated kinesin KIF4 | 45.933 | 31.333 | -0.500 | 0.082 | 0.141 |
| Sema4d | O09126 | Semaphorin-4D | 35.333 | 41.067 | 0.156 | 0.526 | 0.644 |
| Afdn | Q9QZQ1 | Afadin | 137.533 | 144.933 | 0.217 | 0.474 | 0.596 |
| Ttc28 | Q80XJ3 | Tetratricopeptide repeat protein 28 | 26.600 | 24.867 | -0.231 | 0.421 | 0.542 |
| Col4a4 | Q9QZR9 | Collagen alpha-4(IV) chain | 49.800 | 36.600 | -0.505 | 0.174 | 0.271 |
| Nup93 | Q8BJ71 | Nuclear pore complex protein Nup93 | 31.400 | 34.133 | 0.062 | 0.854 | 0.897 |
| Ankrd17 | Q99NH0 | Ankyrin repeat domain-containing protein 17 | 44.533 | 26.333 | -0.777 | 0.080 | 0.139 |
| Nktr | P30415 | NK-tumor recognition protein | 97.800 | 69.933 | -0.337 | 0.520 | 0.640 |
| Sptb | P15508 | Spectrin beta chain, erythrocytic | 93.133 | 74.333 | -0.422 | 0.167 | 0.263 |
| Rp1l1 | Q8CGM2 | Retinitis pigmentosa 1-like 1 protein | 39.400 | 32.867 | -0.283 | 0.170 | 0.267 |
| Plxnb1 | Q8CJH3 | Plexin-B1 | 62.867 | 78.800 | 0.390 | 0.178 | 0.275 |
| Tenm4 | Q3UHK6 | Teneurin-4 | 120.467 | 94.067 | -0.379 | 0.249 | 0.359 |
| Fhad1 | A6PWD2 | Forkhead-associated domain-containing protein 1 | 78.733 | 63.933 | -0.279 | 0.122 | 0.202 |
| Mki67 | E9PVX6 | Proliferation marker protein Ki-67 | 175.733 | 184.400 | 0.052 | 0.853 | 0.899 |
| Ncapg2 | Q6DFV1 | Condensin-2 complex subunit G2 | 73.933 | 85.933 | 0.505 | 0.253 | 0.364 |
| Atm | Q62388 | Serine-protein kinase ATM | 91.000 | 90.067 | 0.049 | 0.793 | 0.857 |
| Kdm5a | Q3UXZ9 | Lysine-specific demethylase 5A | 35.267 | 71.800 | 0.493 | 0.199 | 0.303 |
| Dnah17 | Q69Z23 | Dynein heavy chain 17, axonemal | 73.400 | 75.000 | 0.072 | 0.798 | 0.860 |
| Atr | Q9JKK8 | Serine/threonine-protein kinase ATR | 84.733 | 58.267 | -0.439 | 0.361 | 0.481 |
| Fancd2 | Q80V62 | Fanconi anemia group D2 protein homolog | 53.200 | 48.200 | -0.053 | 0.823 | 0.878 |
| Skt | A2AQ25 | Sickle tail protein | 57.733 | 64.600 | -0.138 | 0.762 | 0.836 |
| Senp5 | Q6NXL6 | Sentrin-specific protease 5 | 38.467 | 37.400 | -0.115 | 0.733 | 0.814 |
| Col4a1 | P02463 | Collagen alpha-1(IV) chain | 29.600 | 23.733 | -0.403 | 0.070 | 0.127 |
| Mphosph8 | Q3TYA6 | M-phase phosphoprotein 8 | 55.533 | 44.533 | 0.717 | 0.222 | 0.326 |
| Ubn2 | Q80WC1 | Ubinuclein-2 | 54.267 | 43.533 | 0.575 | 0.233 | 0.338 |
| Nbea | Q9EPN1 | Neurobeachin | 56.333 | 65.400 | 0.126 | 0.621 | 0.738 |
| Rock2 | P70336 | Rho-associated protein kinase 2 | 128.067 | 89.267 | -0.535 | 0.052 | 0.098 |
| Pde11a | P0C1Q2 | Dual 3',5'-cyclic-AMP and -GMP phosphodiesterase 11A | 36.867 | 36.933 | -0.367 | 0.286 | 0.397 |
| Abcc5 | Q9R1X5 | Multidrug resistance-associated protein 5 | 44.200 | 54.800 | 0.565 | 0.215 | 0.316 |
| Nemf | Q8CCP0 | Nuclear export mediator factor Nemf | 43.200 | 51.067 | 0.096 | 0.763 | 0.836 |
| Ankrd26 | Q811D2 | Ankyrin repeat domain-containing protein 26 | 141.867 | 98.467 | -0.273 | 0.503 | 0.620 |
| Kif15 | Q6P9L6 | Kinesin-like protein KIF15 | 38.733 | 30.333 | -0.482 | 0.060 | 0.112 |
| Smc1b | Q920F6 | Structural maintenance of chromosomes protein 1B | 166.467 | 167.867 | 0.037 | 0.853 | 0.898 |
| Fer1l4 | A3KGK3 | Fer-1-like protein 4 | 54.400 | 63.400 | -0.022 | 0.953 | 0.957 |
| Zfhx4 | Q9JJN2 | Zinc finger homeobox protein 4 | 80.933 | 83.867 | 0.137 | 0.536 | 0.652 |
| Ncor1 | Q60974 | Nuclear receptor corepressor 1 | 79.933 | 63.267 | -0.214 | 0.623 | 0.740 |
| Kdm3a | Q6PCM1 | Lysine-specific demethylase 3A | 45.267 | 56.400 | 0.341 | 0.236 | 0.342 |
| Nat10 | Q8K224 | RNA cytidine acetyltransferase | 38.000 | 33.933 | -0.095 | 0.768 | 0.839 |
| Utp14a | Q640M1 | U3 small nucleolar RNA-associated protein 14 homolog A | 52.533 | 40.600 | -0.408 | 0.138 | 0.224 |
| Dpysl4 | O35098 | Dihydropyrimidinase-related protein 4 | 33.133 | 34.467 | 0.042 | 0.839 | 0.891 |
| Prg4 | Q9JM99 | Proteoglycan 4 | 50.733 | 38.000 | -0.204 | 0.592 | 0.705 |
| Anks1b | Q8BIZ1 | Ankyrin repeat and sterile alpha motif domain-containing protein 1B | 20.667 | 23.067 | 0.049 | 0.842 | 0.892 |
| Lrrcc1 | Q69ZB0 | Leucine-rich repeat and coiled-coil domain-containing protein 1 | 34.467 | 38.800 | 0.096 | 0.638 | 0.752 |
| Ptprb | B2RU80 | Receptor-type tyrosine-protein phosphatase beta | 84.000 | 75.667 | 0.167 | 0.576 | 0.691 |
| Filip1l | Q6P6L0 | Filamin A-interacting protein 1-like | 53.667 | 50.600 | -0.323 | 0.489 | 0.609 |
| Clspn | Q80YR7 | Claspin | 41.600 | 43.267 | -0.030 | 0.897 | 0.924 |
| Ccdc80 | Q8R2G6 | Coiled-coil domain-containing protein 80 | 55.333 | 42.000 | -0.592 | 0.066 | 0.121 |
| Micu1 | Q8VCX5 | Calcium uptake protein 1, mitochondrial | 64.000 | 52.867 | -0.383 | 0.444 | 0.566 |
| Mis18bp1 | Q80WQ8 | Mis18-binding protein 1 | 86.000 | 77.933 | -0.175 | 0.549 | 0.666 |
| Chd1l | Q9CXF7 | Chromodomain-helicase-DNA-binding protein 1-like | 40.600 | 37.333 | -0.382 | 0.288 | 0.397 |
| Dnai1 | Q8C0M8 | Dynein intermediate chain 1, axonemal | 25.267 | 23.267 | -0.020 | 0.937 | 0.950 |
| Kctd19 | Q562E2 | BTB/POZ domain-containing protein KCTD19 | 41.800 | 68.400 | 0.814 | 0.088 | 0.150 |
| Trrap | Q80YV3 | Transformation/transcription domain-associated protein | 84.267 | 130.467 | 0.379 | 0.492 | 0.610 |
| Sycp2 | Q9CUU3 | Synaptonemal complex protein 2 | 98.067 | 89.133 | -0.080 | 0.825 | 0.879 |
| Golga4 | Q91VW5 | Golgin subfamily A member 4 | 83.467 | 92.867 | 0.165 | 0.306 | 0.414 |
| Pcm1 | Q9R0L6 | Pericentriolar material 1 protein | 35.067 | 39.667 | 0.290 | 0.396 | 0.520 |
| Rad50 | P70388 | DNA repair protein RAD50 | 57.733 | 37.400 | -0.256 | 0.580 | 0.693 |
| Adgrg4 | B7ZCC9 | Adhesion G-protein coupled receptor G4 | 41.867 | 40.400 | -0.056 | 0.781 | 0.848 |
| Zfyve26 | Q5DU37 | Zinc finger FYVE domain-containing protein 26 | 28.600 | 29.267 | -0.055 | 0.817 | 0.877 |
| Col6a5 | A6H584 | Collagen alpha-5(VI) chain | 93.933 | 90.467 | -0.021 | 0.923 | 0.941 |
| Map7d3 | A2AEY4 | MAP7 domain-containing protein 3 | 47.467 | 46.600 | 0.191 | 0.628 | 0.743 |
| Nsd1 | O88491 | Histone-lysine N-methyltransferase, H3 lysine-36 and H4 lysine-20 specific | 86.467 | 88.467 | 0.084 | 0.878 | 0.919 |
| Hirip3 | Q8BLH7 | HIRA-interacting protein 3 | 77.200 | 54.067 | -0.297 | 0.569 | 0.685 |
| Frem2 | Q6NVD0 | FRAS1-related extracellular matrix protein 2 | 46.733 | 36.267 | -0.309 | 0.392 | 0.515 |
| Cfap58 | B2RW38 | Cilia- and flagella-associated protein 58 | 42.600 | 35.400 | -0.282 | 0.198 | 0.303 |
| Col4a2 | P08122 | Collagen alpha-2(IV) chain | 96.600 | 88.400 | -0.213 | 0.653 | 0.762 |
| Srpra | Q9DBG7 | Signal recognition particle receptor subunit alpha | 44.267 | 41.600 | -0.103 | 0.724 | 0.815 |
| R3hdm2 | Q80TM6 | R3H domain-containing protein 2 | 68.267 | 56.667 | -0.349 | 0.141 | 0.227 |
| Abcb5 | B5X0E4 | ATP-binding cassette sub-family B member 5 | 40.133 | 41.867 | -0.019 | 0.945 | 0.954 |
| Samd9l | Q69Z37 | Sterile alpha motif domain-containing protein 9-like | 77.133 | 50.667 | -0.570 | 0.058 | 0.111 |
| Nuak2 | Q8BZN4 | NUAK family SNF1-like kinase 2 | 92.200 | 76.267 | -0.198 | 0.498 | 0.617 |
| Igsf10 | Q3V1M1 | Immunoglobulin superfamily member 10 | 79.133 | 66.400 | -0.304 | 0.177 | 0.274 |
| Neil3 | Q8K203 | Endonuclease 8-like 3 | 30.000 | 29.000 | -0.131 | 0.631 | 0.745 |
| Pole | Q9WVF7 | DNA polymerase epsilon catalytic subunit A | 66.733 | 49.467 | -0.508 | 0.281 | 0.394 |
| Col5a2 | Q3U962 | Collagen alpha-2(V) chain | 38.667 | 35.200 | -0.093 | 0.725 | 0.815 |
| #VALUE! | E0CYV9 | Uncharacterized protein C4orf54 homolog | 48.133 | 50.400 | 0.012 | 0.976 | 0.977 |
| Kndc1 | Q0KK55 | Kinase non-catalytic C-lobe domain-containing protein 1 | 49.267 | 37.800 | -0.391 | 0.211 | 0.314 |
| Dab2ip | Q3UHC7 | Disabled homolog 2-interacting protein | 49.733 | 72.133 | 0.440 | 0.093 | 0.158 |
| Ubr4 | A2AN08 | E3 ubiquitin-protein ligase UBR4 | 75.667 | 68.067 | -0.179 | 0.707 | 0.800 |
| Tdrd5 | Q5VCS6 | Tudor domain-containing protein 5 | 112.467 | 65.600 | -0.798 | 0.083 | 0.143 |
| Prex1 | Q69ZK0 | Phosphatidylinositol 3,4,5-trisphosphate-dependent Rac exchanger 1 protein | 66.067 | 42.333 | -0.804 | 0.063 | 0.117 |
| Kmt2c | Q8BRH4 | Histone-lysine N-methyltransferase 2C | 99.067 | 114.400 | 0.222 | 0.477 | 0.599 |
| Prpf4b | Q61136 | Serine/threonine-protein kinase PRP4 homolog | 38.133 | 38.400 | -0.062 | 0.800 | 0.861 |
| Ank3 | G5E8K5 | Ankyrin-3 | 58.600 | 54.600 | 0.093 | 0.813 | 0.874 |
| Myo3a | Q8K3H5 | Myosin-IIIa | 65.200 | 81.267 | 0.297 | 0.402 | 0.525 |
| Lrrk2 | Q5S006 | Leucine-rich repeat serine/threonine-protein kinase 2 | 65.267 | 46.267 | -0.337 | 0.386 | 0.512 |
| Col16a1 | Q8BLX7 | Collagen alpha-1(XVI) chain | 44.133 | 29.933 | -0.528 | 0.110 | 0.184 |
| Cenpc | P49452 | Centromere protein C | 66.400 | 76.067 | -0.081 | 0.821 | 0.879 |
| Trpm1 | Q2TV84 | Transient receptor potential cation channel subfamily M member 1 | 61.133 | 53.933 | -0.040 | 0.896 | 0.925 |
| Arhgef10 | Q8C033 | Rho guanine nucleotide exchange factor 10 | 46.800 | 67.000 | -0.080 | 0.881 | 0.919 |
| Znf518b | B2RRE4 | Zinc finger protein 518B | 34.000 | 27.133 | -0.303 | 0.223 | 0.326 |
| Slk | O54988 | STE20-like serine/threonine-protein kinase | 44.333 | 48.133 | 0.047 | 0.879 | 0.919 |
| Pol | P11369 | LINE-1 retrotransposable element ORF2 protein | 48.600 | 66.533 | 0.216 | 0.669 | 0.771 |
| Vps53 | Q8CCB4 | Vacuolar protein sorting-associated protein 53 homolog | 59.467 | 55.333 | -0.037 | 0.892 | 0.923 |
| Prpf40a | Q9R1C7 | Pre-mRNA-processing factor 40 homolog A | 43.000 | 44.333 | 0.169 | 0.540 | 0.656 |
| Ccnb3 | Q810T2 | G2/mitotic-specific cyclin-B3 | 31.200 | 28.200 | -0.386 | 0.206 | 0.308 |
| Dnah1 | E9Q8T7 | Dynein heavy chain 1, axonemal | 156.133 | 122.533 | -0.398 | 0.280 | 0.393 |
| Dlg2 | Q91XM9 | Disks large homolog 2 | 77.600 | 53.000 | -0.673 | 0.066 | 0.122 |
| Ddx58 | Q6Q899 | Probable ATP-dependent RNA helicase DDX58 | 50.067 | 64.533 | 0.245 | 0.344 | 0.462 |
| Cdc7 | Q9Z0H0 | Cell division cycle 7-related protein kinase | 44.800 | 38.133 | -0.424 | 0.117 | 0.195 |
| Cntrl | A2AL36 | Centriolin | 125.200 | 249.467 | 0.822 | 0.070 | 0.127 |
| Sin3a | Q60520 | Paired amphipathic helix protein Sin3a | 19.533 | 24.800 | 0.326 | 0.181 | 0.278 |
| Map1a | Q9QYR6 | Microtubule-associated protein 1A | 32.933 | 31.933 | 0.242 | 0.522 | 0.641 |
| Ddx54 | Q8K4L0 | ATP-dependent RNA helicase DDX54 | 94.600 | 67.400 | -0.741 | 0.090 | 0.154 |
| Smc3 | Q9CW03 | Structural maintenance of chromosomes protein 3 | 112.800 | 101.467 | -0.183 | 0.470 | 0.591 |
| Srp68 | Q8BMA6 | Signal recognition particle subunit SRP68 | 26.267 | 20.467 | -0.279 | 0.280 | 0.394 |
| Spef2 | Q8C9J3 | Sperm flagellar protein 2 | 32.400 | 59.467 | 0.786 | 0.051 | 0.098 |
| Cpsf1 | Q9EPU4 | Cleavage and polyadenylation specificity factor subunit 1 | 21.533 | 24.200 | 0.066 | 0.726 | 0.813 |
| Yars | Q91WQ3 | Tyrosine--tRNA ligase, cytoplasmic | 27.533 | 28.733 | 0.130 | 0.500 | 0.618 |
| Myo18a | Q9JMH9 | Unconventional myosin-XVIIIa | 104.867 | 79.200 | -0.399 | 0.165 | 0.261 |
| Arhgap24 | Q8C4V1 | Rho GTPase-activating protein 24 | 36.467 | 56.267 | 0.352 | 0.260 | 0.372 |
| Lyst | P97412 | Lysosomal-trafficking regulator | 115.267 | 91.267 | -0.561 | 0.086 | 0.148 |
| Nolc1 | E9Q5C9 | Nucleolar and coiled-body phosphoprotein 1 | 57.867 | 54.067 | -0.345 | 0.509 | 0.628 |
| Smc5 | Q8CG46 | Structural maintenance of chromosomes protein 5 | 24.067 | 32.133 | 0.443 | 0.087 | 0.149 |
| Map1b | P14873 | Microtubule-associated protein 1B | 139.667 | 165.000 | 0.207 | 0.297 | 0.410 |
| Cgnl1 | Q6AW69 | Cingulin-like protein 1 | 27.800 | 30.800 | 0.018 | 0.947 | 0.955 |
| Frmd4b | Q920B0 | FERM domain-containing protein 4B | 92.467 | 139.267 | 0.437 | 0.157 | 0.249 |
| Iqsec2 | Q5DU25 | IQ motif and SEC7 domain-containing protein 2 | 66.533 | 67.333 | 0.183 | 0.529 | 0.644 |
| Gldc | Q91W43 | Glycine dehydrogenase (decarboxylating), mitochondrial | 53.733 | 67.533 | -0.049 | 0.905 | 0.928 |
| Scyl2 | Q8CFE4 | SCY1-like protein 2 | 36.733 | 35.000 | -0.022 | 0.948 | 0.955 |
| Aldh18a1 | Q9Z110 | Delta-1-pyrroline-5-carboxylate synthase | 34.200 | 27.133 | -0.423 | 0.136 | 0.222 |
| Utp20 | Q5XG71 | Small subunit processome component 20 homolog | 140.667 | 163.667 | 0.266 | 0.203 | 0.305 |
| Myo1f | P70248 | Unconventional myosin-If | 50.000 | 33.667 | -0.638 | 0.185 | 0.284 |
| Mark2 | Q05512 | Serine/threonine-protein kinase MARK2 | 150.600 | 136.267 | -0.686 | 0.230 | 0.334 |
| Dock2 | Q8C3J5 | Dedicator of cytokinesis protein 2 | 81.200 | 131.733 | 0.631 | 0.072 | 0.129 |
| Fastkd1 | Q6DI86 | FAST kinase domain-containing protein 1, mitochondrial | 33.267 | 46.733 | 0.439 | 0.137 | 0.222 |
| Akap13 | E9Q394 | A-kinase anchor protein 13 | 83.467 | 86.933 | 0.033 | 0.906 | 0.928 |
| Akap12 | Q9WTQ5 | A-kinase anchor protein 12 | 89.200 | 89.067 | 0.510 | 0.288 | 0.396 |
| Lgmn | O89017 | Legumain | 32.867 | 36.600 | 0.152 | 0.525 | 0.643 |
| Map3k4 | O08648 | Mitogen-activated protein kinase kinase kinase 4 | 57.667 | 42.600 | -0.363 | 0.404 | 0.526 |
| Ctnnd2 | O35927 | Catenin delta-2 | 31.600 | 45.333 | 0.059 | 0.909 | 0.931 |
| Dcst1 | Q059Y8 | E3 ubiquitin-protein ligase DCST1 | 45.333 | 68.000 | 0.279 | 0.447 | 0.569 |
| Igsf3 | Q6ZQA6 | Immunoglobulin superfamily member 3 | 56.000 | 40.600 | -0.457 | 0.077 | 0.138 |
| Dyrk4 | Q8BI55 | Dual specificity tyrosine-phosphorylation-regulated kinase 4 | 44.667 | 37.600 | -0.223 | 0.413 | 0.531 |
| Kdm4a | Q8BW72 | Lysine-specific demethylase 4A | 37.933 | 26.000 | -0.390 | 0.283 | 0.394 |
| Zdbf2 | Q5SS00 | DBF4-type zinc finger-containing protein 2 homolog | 39.533 | 45.000 | 0.138 | 0.734 | 0.814 |
| Twf1 | Q91YR1 | Twinfilin-1 | 22.000 | 23.933 | 0.064 | 0.723 | 0.815 |
| Nfrkb | Q6PIJ4 | Nuclear factor related to kappa-B-binding protein | 50.733 | 32.000 | -0.290 | 0.462 | 0.584 |
| Ascc3 | E9PZJ8 | Activating signal cointegrator 1 complex subunit 3 | 56.000 | 46.733 | -0.608 | 0.126 | 0.208 |
| Ofd1 | Q80Z25 | Oral-facial-digital syndrome 1 protein homolog | 23.333 | 28.533 | 0.239 | 0.222 | 0.325 |
| Vps13b | Q80TY5 | Vacuolar protein sorting-associated protein 13B | 31.667 | 42.867 | 0.642 | 0.079 | 0.139 |
| Carmil1 | Q6EDY6 | F-actin-uncapping protein LRRC16A | 36.800 | 40.133 | 0.136 | 0.574 | 0.689 |
| Pkd1l2 | Q7TN88 | Polycystic kidney disease protein 1-like 2 | 61.133 | 61.000 | -0.032 | 0.927 | 0.943 |
| Prpf6 | Q91YR7 | Pre-mRNA-processing factor 6 | 42.067 | 58.267 | 0.477 | 0.069 | 0.124 |
| Ralbp1 | Q62172 | RalA-binding protein 1 | 79.333 | 53.133 | -0.539 | 0.119 | 0.198 |
| Ogt | Q8CGY8 | UDP-N-acetylglucosamine--peptide N-acetylglucosaminyltransferase 110 kDa subunit | 43.867 | 50.533 | 0.249 | 0.565 | 0.680 |
| Arid1a | A2BH40 | AT-rich interactive domain-containing protein 1A | 54.533 | 45.733 | -0.197 | 0.389 | 0.511 |
| Usp33 | Q8R5K2 | Ubiquitin carboxyl-terminal hydrolase 33 | 56.467 | 48.600 | -0.360 | 0.355 | 0.475 |
| Cdk12 | Q14AX6 | Cyclin-dependent kinase 12 | 62.267 | 87.600 | 0.215 | 0.553 | 0.670 |
| Ahctf1 | Q8CJF7 | Protein ELYS | 52.267 | 58.333 | 0.126 | 0.667 | 0.771 |
| Map9 | Q3TRR0 | Microtubule-associated protein 9 | 41.800 | 38.333 | -0.111 | 0.699 | 0.795 |
| Ppp1r12a | Q9DBR7 | Protein phosphatase 1 regulatory subunit 12A | 27.200 | 25.200 | -0.081 | 0.693 | 0.790 |
| Wipf2 | Q6PEV3 | WAS/WASL-interacting protein family member 2 | 36.133 | 43.867 | 0.432 | 0.200 | 0.301 |
| Cdk13 | Q69ZA1 | Cyclin-dependent kinase 13 | 39.400 | 30.667 | -0.229 | 0.381 | 0.506 |
| Ddx24 | Q9ESV0 | ATP-dependent RNA helicase DDX24 | 28.600 | 29.467 | 0.039 | 0.874 | 0.917 |
| Shank1 | D3YZU1 | SH3 and multiple ankyrin repeat domains protein 1 | 56.867 | 40.267 | -0.252 | 0.411 | 0.532 |
| Ccdc38 | Q8CDN8 | Coiled-coil domain-containing protein 38 | 47.733 | 51.933 | -0.179 | 0.757 | 0.837 |
| #VALUE! | Q8CEZ4 | Uncharacterized protein C2orf54 homolog | 40.933 | 43.333 | -0.102 | 0.721 | 0.815 |
| Col6a6 | Q8C6K9 | Collagen alpha-6(VI) chain | 79.867 | 71.000 | -0.387 | 0.172 | 0.269 |
| Chrdl1 | Q920C1 | Chordin-like protein 1 | 40.600 | 35.200 | -0.206 | 0.499 | 0.618 |
| Znf445 | Q8R2V3 | Zinc finger protein 445 | 95.200 | 122.867 | 0.266 | 0.651 | 0.762 |
| Grm4 | Q68EF4 | Metabotropic glutamate receptor 4 | 23.600 | 27.667 | 0.211 | 0.301 | 0.412 |
| Znf330 | Q922H9 | Zinc finger protein 330 | 45.133 | 53.600 | 0.212 | 0.214 | 0.317 |
| Arhgef12 | Q8R4H2 | Rho guanine nucleotide exchange factor 12 | 78.200 | 83.467 | 0.068 | 0.789 | 0.855 |
| Itih5 | Q8BJD1 | Inter-alpha-trypsin inhibitor heavy chain H5 | 37.467 | 48.733 | 0.249 | 0.356 | 0.475 |
| Iqca1 | Q9CUL5 | Dynein regulatory complex protein 11 | 46.667 | 32.533 | -0.284 | 0.307 | 0.415 |
| Strip2 | Q8C9H6 | Striatin-interacting proteins 2 | 96.800 | 107.133 | -0.049 | 0.882 | 0.917 |
| Rock1 | P70335 | Rho-associated protein kinase 1 | 97.000 | 59.533 | -0.475 | 0.255 | 0.365 |
| Stpg4 | Q9DAG5 | Protein STPG4 | 27.133 | 26.533 | -0.060 | 0.839 | 0.890 |
| Pskh1 | Q91YA2 | Serine/threonine-protein kinase H1 | 43.200 | 27.200 | -0.629 | 0.067 | 0.123 |
| Sytl3 | Q99N48 | Synaptotagmin-like protein 3 | 34.400 | 43.800 | 0.107 | 0.807 | 0.867 |
| Aurkaip1 | Q9DCJ7 | Aurora kinase A-interacting protein | 39.000 | 26.667 | -0.519 | 0.132 | 0.217 |
| Fcrl5 | Q68SN8 | Fc receptor-like protein 5 | 33.800 | 31.600 | -0.160 | 0.411 | 0.531 |
| Zfp57 | Q8C6P8 | Zinc finger protein 57 | 26.467 | 24.733 | -0.087 | 0.628 | 0.742 |
| Arhgap31 | A6X8Z5 | Rho GTPase-activating protein 31 | 54.600 | 47.667 | -0.685 | 0.077 | 0.137 |
| Col20a1 | Q923P0 | Collagen alpha-1(XX) chain | 40.733 | 44.800 | -0.177 | 0.585 | 0.699 |
| Epb41l2 | O70318 | Band 4.1-like protein 2 | 25.133 | 20.533 | -0.434 | 0.078 | 0.139 |
| Polr1b | P70700 | DNA-directed RNA polymerase I subunit RPA2 | 63.133 | 69.533 | -0.089 | 0.776 | 0.845 |
| Ttbk1 | Q6PCN3 | Tau-tubulin kinase 1 | 23.000 | 28.867 | 0.414 | 0.238 | 0.345 |
| Fas | P25446 | Tumor necrosis factor receptor superfamily member 6 | 54.000 | 46.000 | -0.396 | 0.341 | 0.460 |
| Polr3c | Q9D483 | DNA-directed RNA polymerase III subunit RPC3 | 122.533 | 152.533 | 0.407 | 0.068 | 0.124 |
| Htr3b | Q9JHJ5 | 5-hydroxytryptamine receptor 3B | 37.200 | 36.600 | 0.018 | 0.949 | 0.955 |
| Scmh1 | Q8K214 | Polycomb protein SCMH1 | 54.133 | 45.733 | -0.304 | 0.432 | 0.551 |
| Zfp28 | P10078 | Zinc finger protein 28 | 22.667 | 32.400 | 0.522 | 0.080 | 0.140 |
| Chrm3 | Q9ERZ3 | Muscarinic acetylcholine receptor M3 | 55.467 | 42.933 | -0.330 | 0.388 | 0.511 |
| Frmpd4 | A2AFR3 | FERM and PDZ domain-containing protein 4 | 32.467 | 38.400 | 0.023 | 0.941 | 0.953 |
| Cep57l1 | Q8VDS7 | Centrosomal protein CEP57L1 | 82.600 | 73.267 | -0.231 | 0.513 | 0.631 |
| Top1mt | Q8R4U6 | DNA topoisomerase I, mitochondrial | 31.067 | 28.667 | -0.090 | 0.780 | 0.848 |
| Sh2d4a | Q9D7V1 | SH2 domain-containing protein 4A | 19.133 | 23.133 | 0.300 | 0.307 | 0.414 |
| Map7d1 | A2AJI0 | MAP7 domain-containing protein 1 | 59.733 | 44.200 | -0.331 | 0.314 | 0.422 |
| Luzp1 | Q8R4U7 | Leucine zipper protein 1 | 69.533 | 76.733 | -0.049 | 0.901 | 0.926 |
| Mdga2 | P60755 | MAM domain-containing glycosylphosphatidylinositol anchor protein 2 | 39.733 | 31.333 | -0.335 | 0.213 | 0.316 |
| Plk3 | Q60806 | Serine/threonine-protein kinase PLK3 | 24.467 | 25.933 | 0.134 | 0.486 | 0.605 |
| Nuf2 | Q99P69 | Kinetochore protein Nuf2 | 31.667 | 29.400 | -0.311 | 0.263 | 0.374 |

**Supplementary Table 3**

**Proteins assigned to functional clusters detected in manually dissected fibres originating from WT and cNEB KO mice.**

Metascape determined both enrichment and p values for protein clusters and associated proteins. Visual representation of pathway clusters found in Fig. 4.

| *Cluster name* | *Protein number* | *Proteins in cluster* | *Up in WT or cNEB ko* | *Best enrich-ment in cluster* | *Best Log p in cluster* |
| --- | --- | --- | --- | --- | --- |
| *aerobic respiration* | 24 | ATP synthase subunit alpha, mitochondrial | cNEB ko | 66.80 | -17.83 |
|  |  | ATP synthase subunit beta, mitochondrial |  |  |  |
|  |  | ATP synthase subunit gamma, mitochondrial |  |  |  |
|  |  | ATP synthase F(0) complex subunit B1, mitochondrial |  |  |  |
|  |  | RB1-inducible coiled-coil protein 1 |  |  |  |
|  |  | Cytochrome c oxidase subunit 4 isoform 1, mitochondrial |  |  |  |
|  |  | Cytochrome c oxidase subunit 5A, mitochondrial |  |  |  |
|  |  | Cytochrome c, somatic |  |  |  |
|  |  | DNA-directed RNA polymerase II subunit RPB1 |  |  |  |
|  |  | Tubulin alpha-4A chain |  |  |  |
|  |  | Voltage-dependent anion-selective channel protein 1 |  |  |  |
|  |  | ATP synthase subunit O, mitochondrial |  |  |  |
|  |  | Succinate Dehydrogenase Complex Subunit C |  |  |  |
|  |  | Cytochrome c1, heme protein, mitochondrial |  |  |  |
|  |  | Succinate dehydrogenase [ubiquinone] flavoprotein subunit, mitochondrial |  |  |  |
|  |  | Succinate dehydrogenase [ubiquinone] iron-sulfur subunit, mitochondrial |  |  |  |
|  |  | ATP synthase subunit d, mitochondrial |  |  |  |
|  |  | Aconitate hydratase, mitochondrial |  |  |  |
|  |  | Malate dehydrogenase, cytoplasmic |  |  |  |
|  |  | Pyruvate dehydrogenase E1 component subunit alpha |  |  |  |
|  |  | Plectin |  |  |  |
|  |  | Dihydrolipoamide S-Succinyltransferase |  |  |  |
|  |  | Ubiquitin-40S ribosomal protein S27a |  |  |  |
|  |  | Myosin-7 |  |  |  |
| *TCA cycle* | 9 | Aconitate hydratase, mitochondrial | cNEB ko | 47.71 | -9.83 |
|  |  | Aspartate aminotransferase, mitochondrial |  |  |  |
|  |  | Malate dehydrogenase, cytoplasmic |  |  |  |
|  |  | Pyruvate dehydrogenase E1 component subunit alpha, somatic form, mitochondrial |  |  |  |
|  |  | Succinate Dehydrogenase Complex Subunit C |  |  |  |
|  |  | Succinate dehydrogenase [ubiquinone] flavoprotein subunit, mitochondrial |  |  |  |
|  |  | Succinate dehydrogenase [ubiquinone] iron-sulfur subunit, mitochondrial |  |  |  |
|  |  | Dihydrolipoamide S-Succinyltransferase |  |  |  |
|  |  | Voltage-dependent anion-selective channel protein 1 |  |  |  |
| aerobic electron transport chain | 9 | Cytochrome c oxidase subunit 4 isoform 1, mitochondrial | cNEB ko | 150.30 | -7.71 |
|  |  | Cytochrome c oxidase subunit 5A, mitochondrial |  |  |  |
|  |  | Cytochrome c, somatic |  |  |  |
|  |  | Plectin |  |  |  |
|  |  | Succinate Dehydrogenase Complex Subunit C |  |  |  |
|  |  | Cytochrome c1 |  |  |  |
|  |  | Succinate dehydrogenase [ubiquinone] flavoprotein subunit, mitochondrial |  |  |  |
|  |  | Succinate dehydrogenase [ubiquinone] iron-sulfur subunit, mitochondrial |  |  |  |
|  |  | Serum albumin |  |  |  |
| muscle cell development | 27 | Actin, alpha skeletal muscle | cNEB ko | 23.86 | -7.46 |
|  |  | Alpha-actinin-2 |  |  |  |
|  |  | Alpha-actinin-3 |  |  |  |
|  |  | Alpha-crystallin B chain |  |  |  |
|  |  | Four and a half LIM domains protein 1 |  |  |  |
|  |  | Low-density lipoprotein receptor-related protein 2 |  |  |  |
|  |  | Plectin |  |  |  |
|  |  | LIM domain-binding protein 3 |  |  |  |
|  |  | Myc box-dependent-interacting protein 1 |  |  |  |
|  |  | PDZ and LIM domain protein 3 |  |  |  |
|  |  | PDZ and LIM domain protein 5 |  |  |  |
|  |  | Myozenin-1 |  |  |  |
|  |  | Filamin-C |  |  |  |
|  |  | Ankyrin-2 |  |  |  |
|  |  | Myosin-7 |  |  |  |
|  |  | Endoplasmin |  |  |  |
|  |  | Harmonin |  |  |  |
|  |  | RB1-inducible coiled-coil protein 1 |  |  |  |
|  |  | Vascular endothelial growth factor receptor 2 |  |  |  |
|  |  | Myoglobin |  |  |  |
|  |  | Reticulon-4 |  |  |  |
|  |  | Deleted in autism protein 1 homolog |  |  |  |
|  |  | Cytoplasmic dynein 2 heavy chain 1 |  |  |  |
|  |  | Abnormal spindle-like microcephaly-associated protein homolog |  |  |  |
|  |  | 40S ribosomal protein S5 |  |  |  |
|  |  | Kinetochore-associated protein 1 |  |  |  |
|  |  | 60S ribosomal protein L10-like |  |  |  |
| *Cellular responses to stress* | 16 | Serum albumin | cNEB ko | 12.52 | -5.02 |
|  |  | Cytochrome c oxidase subunit 4 isoform 1, mitochondrial |  |  |  |
|  |  | Cytochrome c oxidase subunit 5A, mitochondrial |  |  |  |
|  |  | Alpha-crystallin B chain |  |  |  |
|  |  | Cytochrome c, somatic |  |  |  |
|  |  | Histone H1.3 |  |  |  |
|  |  | Heat shock protein HSP 90-beta |  |  |  |
|  |  | Tubulin alpha-4A chain |  |  |  |
|  |  | Histone H4 |  |  |  |
|  |  | Ubiquitin-40S ribosomal protein S27a |  |  |  |
|  |  | Histone H1.1 |  |  |  |
|  |  | Histone H2B type 2- |  |  |  |
|  |  | Transcriptional regulator ATRX |  |  |  |
|  |  | Kinetochore-associated protein 1 |  |  |  |
|  |  | Histone H2B type 1-P |  |  |  |
|  |  | Plectin |  |  |  |
| *transition between fast and slow fiber* | 11 | Alpha-actinin-2 | cNEB ko | 60.12 | -4.85 |
|  |  | Alpha-actinin-3 |  |  |  |
|  |  | Myosin light chain 1/3, skeletal muscle isoform |  |  |  |
|  |  | PDZ and LIM domain protein 5 |  |  |  |
|  |  | Myozenin-1 |  |  |  |
|  |  | Ankyrin-2 |  |  |  |
|  |  | Myosin-7 |  |  |  |
|  |  | Laminin subunit alpha-2 |  |  |  |
|  |  | Myc box-dependent-interacting protein 1 |  |  |  |
|  |  | Actin, alpha skeletal muscle |  |  |  |
|  |  | General transcription factor II-I repeat domain-containing protein 2 |  |  |  |
| *post-embryonic animal morphogenesis* | 10 | Abnormal spindle-like microcephaly-associated protein homolog | cNEB ko | 31.64 | -3.96 |
|  |  | Heat shock protein HSP 90-beta |  |  |  |
|  |  | Vascular endothelial growth factor receptor 2 |  |  |  |
|  |  | Plectin |  |  |  |
|  |  | Transcriptional regulator ATRX |  |  |  |
|  |  | PDZ and LIM domain protein 5 |  |  |  |
|  |  | Deleted in autism protein 1 homolog |  |  |  |
|  |  | Kinesin-like protein KIF26B |  |  |  |
|  |  | Low-density lipoprotein receptor-related protein 2 |  |  |  |
|  |  | Testis-expressed protein 15 |  |  |  |
| *negative regulation of reactive oxygen species metabolic process* | 7 | Alpha-crystallin B chain | cNEB ko | 10.74 | -3.27 |
|  |  | Cytochrome c, somatic |  |  |  |
|  |  | 60 kDa heat shock protein, mitochondrial |  |  |  |
|  |  | Voltage-dependent anion-selective channel protein 1 |  |  |  |
|  |  | CASP8-associated protein 2 |  |  |  |
|  |  | Phosphate carrier protein, mitochondrial |  |  |  |
|  |  | Polyribonucleotide nucleotidyltransferase 1, mitochondrial |  |  |  |
| *Salmonella infection* | 7 | Cytochrome c, somatic | cNEB ko | 5.545 | -3.55 |
|  |  | Heat shock protein HSP 90-beta |  |  |  |
|  |  | FYVE and coiled-coil domain-containing protein 1 |  |  |  |
|  |  | Endoplasmin |  |  |  |
|  |  | Tubulin alpha-4A chain |  |  |  |
|  |  | Filamin-C |  |  |  |
|  |  | Cytoplasmic dynein 2 heavy chain 1 |  |  |  |
| *positive regulation of catabolic process* | 10 | Alpha-actinin-3 | cNEB ko | 7.91 | -3.54 |
|  |  | RB1-inducible coiled-coil protein 1 |  |  |  |
|  |  | Vascular endothelial growth factor receptor 2 |  |  |  |
|  |  | FYVE and coiled-coil domain-containing protein 1 |  |  |  |
|  |  | Moesin |  |  |  |
|  |  | Protein kinase C epsilon type |  |  |  |
|  |  | Serine/threonine-protein kinase PLK1 |  |  |  |
|  |  | Voltage-dependent anion-selective channel protein 1 |  |  |  |
|  |  | Polyribonucleotide nucleotidyltransferase 1, mitochondrial |  |  |  |
|  |  | Protein kinase C and casein kinase II substrate protein 3 |  |  |  |
| *The role of GTSE1 in G2/M progression after G2 checkpoint* | 10 | Heat shock protein HSP 90-beta | cNEB ko | 10.83 | -3.28 |
|  |  | Serine/threonine-protein kinase PLK1 |  |  |  |
|  |  | Tubulin alpha-4A chain |  |  |  |
|  |  | Transcriptional regulator ATRX |  |  |  |
|  |  | Histone H4 |  |  |  |
|  |  | Ubiquitin-40S ribosomal protein S27a |  |  |  |
|  |  | Kinetochore-associated protein 1 |  |  |  |
|  |  | Histone H2B type 2- |  |  |  |
|  |  | RB1-inducible coiled-coil protein 1 |  |  |  |
|  |  | Voltage-dependent anion-selective channel protein 1 |  |  |  |
| *negative regulation of calcium ion transport* | 15 | Alpha-actinin-2 | cNEB ko | 10.73 | -3.27 |
|  |  | RB1-inducible coiled-coil protein 1 |  |  |  |
|  |  | Heat shock protein HSP 90-beta |  |  |  |
|  |  | Vascular endothelial growth factor receptor 2 |  |  |  |
|  |  | Protein kinase C epsilon type |  |  |  |
|  |  | Myc box-dependent-interacting protein 1 |  |  |  |
|  |  | Reticulon-4 |  |  |  |
|  |  | Harmonin |  |  |  |
|  |  | FYVE and coiled-coil domain-containing protein 1 |  |  |  |
|  |  | Moesin |  |  |  |
|  |  | Voltage-dependent anion-selective channel protein 1 |  |  |  |
|  |  | Transcriptional regulator ATRX |  |  |  |
|  |  | Four and a half LIM domains protein 1 |  |  |  |
|  |  | Protein kinase C and casein kinase II substrate protein 3 |  |  |  |
|  |  | Ankyrin-2 |  |  |  |
| *protein localization to chromosome* | 13 | Low-density lipoprotein receptor-related protein 2 | cNEB ko | 10.69 | -3.27 |
|  |  | 60 kDa heat shock protein, mitochondrial |  |  |  |
|  |  | Serine/threonine-protein kinase PLK1 |  |  |  |
|  |  | Signal recognition particle receptor subunit beta |  |  |  |
|  |  | Transcriptional regulator ATRX |  |  |  |
|  |  | Reticulon-4 |  |  |  |
|  |  | Nucleolar protein 8 |  |  |  |
|  |  | Testis-expressed protein 15 |  |  |  |
|  |  | Ankyrin-2 |  |  |  |
|  |  | Cytoplasmic dynein 2 heavy chain 1 |  |  |  |
|  |  | Kinetochore-associated protein 1 |  |  |  |
|  |  | Abnormal spindle-like microcephaly-associated protein homolog |  |  |  |
|  |  | 60S ribosomal protein L10-like |  |  |  |
| *positive regulation of epithelial cell migration* | 7 | ATP synthase subunit alpha, mitochondrial | cNEB ko | 10.74 | -2.95 |
|  |  | ATP synthase subunit beta, mitochondrial |  |  |  |
|  |  | Vascular endothelial growth factor receptor 2 |  |  |  |
|  |  | Protein kinase C epsilon type |  |  |  |
|  |  | Reticulon-4 |  |  |  |
|  |  | Alpha-crystallin B chain |  |  |  |
|  |  | Myoglobin |  |  |  |
| *regulation of potassium ion transmembrane transport* | 4 | Alpha-actinin-2 | cNEB ko | 11.56 | -2.93 |
|  |  | Four and a half LIM domains protein 1 |  |  |  |
|  |  | Myc box-dependent-interacting protein 1 |  |  |  |
|  |  | Ankyrin-2 |  |  |  |
| *protein folding* | 8 | Alpha-crystallin B chain | cNEB ko | 6.00 | -2.82 |
|  |  | 60 kDa heat shock protein, mitochondrial |  |  |  |
|  |  | Heat shock protein HSP 90-beta |  |  |  |
|  |  | Serine/threonine-protein kinase PLK1 |  |  |  |
|  |  | Reticulon-4 |  |  |  |
|  |  | Ankyrin-2 |  |  |  |
|  |  | Endoplasmin |  |  |  |
|  |  | Tubulin-specific chaperone D |  |  |  |
| *Vesicle-mediated transport* | 9 | Serum albumin | cNEB ko | 3.19 | -2.68 |
|  |  | Endoplasmin |  |  |  |
|  |  | Tubulin alpha-4A chain |  |  |  |
|  |  | Myc box-dependent-interacting protein 1 |  |  |  |
|  |  | Ubiquitin-40S ribosomal protein S27a |  |  |  |
|  |  | Protein kinase C and casein kinase II substrate protein 3 |  |  |  |
|  |  | Trafficking protein particle complex subunit 12 |  |  |  |
|  |  | Kinesin-like protein KIF26B |  |  |  |
|  |  | DENN domain-containing protein 5B |  |  |  |
| *Inhibition of DNA recombination at telomere* | 8 | Heat shock protein HSP 90-beta | cNEB ko | 11.13 | -2.60 |
|  |  | Pyruvate dehydrogenase E1 component subunit alpha, somatic form, mitochondrial |  |  |  |
|  |  | DNA-directed RNA polymerase II subunit RPB1 |  |  |  |
|  |  | Histone H4 |  |  |  |
|  |  | Histone H2B type 2- |  |  |  |
|  |  | Ubiquitin-40S ribosomal protein S27a |  |  |  |
|  |  | Serine/threonine-protein kinase PLK1 |  |  |  |
|  |  | Transcriptional regulator ATRX |  |  |  |
| *endomembrane system organization* | 9 | Plectin | cNEB ko | 7.24 | -2.48 |
|  |  | Serine/threonine-protein kinase PLK1 |  |  |  |
|  |  | Myc box-dependent-interacting protein 1 |  |  |  |
|  |  | Reticulon-4 |  |  |  |
|  |  | Protein kinase C and casein kinase II substrate protein 3 |  |  |  |
|  |  | Ankyrin-2 |  |  |  |
|  |  | Cytoplasmic dynein 2 heavy chain 1 |  |  |  |
|  |  | Trafficking protein particle complex subunit 12 |  |  |  |
|  |  | Low-density lipoprotein receptor-related protein 2 |  |  |  |
| *regulation of cellular respiration* | 5 | Cytochrome c, somatic | cNEB ko | 9.70 | -2.43 |
|  |  | Laminin subunit alpha-2 |  |  |  |
|  |  | Myosin-7 |  |  |  |
|  |  | Alpha-actinin-3 |  |  |  |
|  |  | Polyribonucleotide nucleotidyltransferase 1, mitochondrial |  |  |  |
| *glucose catabolic process* | 9 | Fructose-bisphosphate aldolase A | wt | 66.92 | -12.50 |
|  |  | Beta-enolase |  |  |  |
|  |  | Glyceraldehyde-3-phosphate dehydrogenase |  |  |  |
|  |  | L-lactate dehydrogenase A chain |  |  |  |
|  |  | ATP-dependent 6-phosphofructokinase, muscle |  |  |  |
|  |  | Phosphoglycerate kinase 1 |  |  |  |
|  |  | Pyruvate kinase PKM |  |  |  |
|  |  | Phosphoglycerate mutase 2 |  |  |  |
| *muscle contraction* | 23 | Voltage-dependent L-type calcium channel subunit alpha-1C | wt | 15.24 | -5.51 |
|  |  | Calsequestrin-1 |  |  |  |
|  |  | Unconventional myosin-Va |  |  |  |
|  |  | Troponin T, fast skeletal muscle |  |  |  |
|  |  | Tropomyosin alpha-1 chain |  |  |  |
|  |  | PDZ and LIM domain protein 7 |  |  |  |
|  |  | Anthrax toxin receptor 1 |  |  |  |
|  |  | Myosin-14 |  |  |  |
|  |  | WASH complex subunit 5 |  |  |  |
|  |  | Serine/threonine-protein kinase MRCK alpha |  |  |  |
|  |  | Protein phosphatase Slingshot homolog 1 |  |  |  |
|  |  | Xin actin-binding repeat-containing protein 2 |  |  |  |
|  |  | FERM domain-containing protein 3 |  |  |  |
|  |  | Filamin-B |  |  |  |
|  |  | Nesprin-2 |  |  |  |
|  |  | Fructose-bisphosphate aldolase A |  |  |  |
|  |  | Myosin-4 |  |  |  |
|  |  | Myosin regulatory light chain 2, skeletal muscle isoform |  |  |  |
|  |  | Phosphoglycerate mutase 2 |  |  |  |
|  |  | Myosin-binding protein C, fast-type |  |  |  |
|  |  | Inositol 1,4,5-trisphosphate receptor type 1 |  |  |  |
|  |  | Dihydroxyacetone phosphate acyltransferase |  |  |  |
|  |  | Polyphosphoinositide phosphatase |  |  |  |
| *microtubule cytoskeleton organization* | 17 | InaD-like protein | wt | 4.87 | -5.30 |
|  |  | Dynein heavy chain 8, axonemal |  |  |  |
|  |  | Dystonin |  |  |  |
|  |  | Glyceraldehyde-3-phosphate dehydrogenase |  |  |  |
|  |  | FAD-dependent oxidoreductase domain-containing protein 2 |  |  |  |
|  |  | Intraflagellar transport protein 88 homolog |  |  |  |
|  |  | Clusterin-associated protein 1 |  |  |  |
|  |  | CDK5 regulatory subunit-associated protein 2 |  |  |  |
|  |  | Centromere protein J |  |  |  |
|  |  | WASH complex subunit 5 |  |  |  |
|  |  | Serine/threonine-protein kinase MRCK |  |  |  |
|  |  | Nesprin-2 |  |  |  |
|  |  | Coiled-coil domain-containing protein 187 |  |  |  |
|  |  | Dynein regulatory complex subunit 7 |  |  |  |
|  |  | Kinesin-like protein KIF21A |  |  |  |
|  |  | Unconventional myosin-Va |  |  |  |
|  |  | Kinesin-like protein KIF14 |  |  |  |
| *organelle assembly* | 19 | Calsequestrin-1 | wt | 7.07 | -4.68 |
|  |  | Dynein heavy chain 8, axonemal |  |  |  |
|  |  | DNA-dependent protein kinase catalytic subunit |  |  |  |
|  |  | Intraflagellar transport protein 88 homolog |  |  |  |
|  |  | Troponin T, fast skeletal muscle |  |  |  |
|  |  | Tropomyosin alpha-1 chain |  |  |  |
|  |  | Spermatogenesis-associated protein 6 |  |  |  |
|  |  | Centrosomal protein of 70 kDa |  |  |  |
|  |  | Protein JBTS17 |  |  |  |
|  |  | Clusterin-associated protein 1 |  |  |  |
|  |  | CDK5 regulatory subunit-associated protein 2 |  |  |  |
|  |  | Centromere protein J |  |  |  |
|  |  | WASH complex subunit 5 |  |  |  |
|  |  | Cytosolic phospholipase A2 gamma |  |  |  |
|  |  | Protein fantom |  |  |  |
|  |  | Dynein regulatory complex subunit 7 |  |  |  |
|  |  | Protein bicaudal C homolog 1 |  |  |  |
|  |  | Inositol 1,4,5-trisphosphate receptor type 1 |  |  |  |
|  |  | Pyruvate kinase PKM |  |  |  |
| *Assembly of collagen fibrils and other multimeric structures* | 4 | Collagen alpha-3(IV) chain | wt | 16.25 | -4.62 |
|  |  | Collagen alpha-1(V) chain |  |  |  |
|  |  | Collagen alpha-1(VII) chain |  |  |  |
|  |  | Dystonin |  |  |  |
| *heart development* | 13 | Voltage-dependent L-type calcium channel subunit alpha-1C | wt | 3.58 | -4.14 |
|  |  | Collagen alpha-1(V) chain |  |  |  |
|  |  | DNA-dependent protein kinase catalytic subunit |  |  |  |
|  |  | Intraflagellar transport protein 88 homolog |  |  |  |
|  |  | Tropomyosin alpha-1 chain |  |  |  |
|  |  | Mediator of RNA polymerase II transcription subunit 12 |  |  |  |
|  |  | PDZ and LIM domain protein 7 |  |  |  |
|  |  | Protein JBTS17 |  |  |  |
|  |  | Clusterin-associated protein 1 |  |  |  |
|  |  | Protein bicaudal C homolog 1 |  |  |  |
|  |  | Xin actin-binding repeat-containing protein 2 |  |  |  |
|  |  | Protein fantom |  |  |  |
|  |  | Protocadherin Fat 4 |  |  |  |
| *supramolecular fiber organization* | 15 | Calsequestrin-1 | wt | 7.39 | -3.83 |
|  |  | Collagen alpha-1(V) chain |  |  |  |
|  |  | Dystonin |  |  |  |
|  |  | Unconventional myosin-Va |  |  |  |
|  |  | WD repeat and FYVE domain-containing protein 3 |  |  |  |
|  |  | Intraflagellar transport protein 88 homolog |  |  |  |
|  |  | Troponin T, fast skeletal muscle |  |  |  |
|  |  | Tropomyosin alpha-1 chain |  |  |  |
|  |  | Centromere protein J |  |  |  |
|  |  | WASH complex subunit 5 |  |  |  |
|  |  | Epiplakin |  |  |  |
|  |  | Xin actin-binding repeat-containing protein 2 |  |  |  |
|  |  | DNA-dependent protein kinase catalytic subunit |  |  |  |
|  |  | CDK5 regulatory subunit-associated protein 2 |  |  |  |
|  |  | Cytosolic phospholipase A2 gamma |  |  |  |
| *corpus callosum development* | 13 | Dihydroxyacetone phosphate acyltransferase | wt | 25.66 | -3.69 |
|  |  | Ninein |  |  |  |
|  |  | DNA-dependent protein kinase catalytic subunit |  |  |  |
|  |  | Receptor-type tyrosine-protein phosphatase S |  |  |  |
|  |  | Intraflagellar transport protein 88 homolog |  |  |  |
|  |  | DNA topoisomerase 2-beta |  |  |  |
|  |  | Protein JBTS17 |  |  |  |
|  |  | Ankyrin repeat domain-containing protein 11 |  |  |  |
|  |  | CDK5 regulatory subunit-associated protein 2 |  |  |  |
|  |  | Protein fantom |  |  |  |
|  |  | Nesprin-2 |  |  |  |
|  |  | Protocadherin Fat 4 |  |  |  |
|  |  | Kinesin-like protein KIF14 |  |  |  |
| *actin filament-based movement* | 6 | Voltage-dependent L-type calcium channel subunit alpha-1C | wt | 15.73 | -3.61 |
|  |  | Unconventional myosin-Va |  |  |  |
|  |  | Tropomyosin alpha-1 chain |  |  |  |
|  |  | Myosin-14 |  |  |  |
|  |  | Nesprin-2 |  |  |  |
|  |  | Protein phosphatase Slingshot homolog 1 |  |  |  |
| *nuclear DNA replication* | 12 | Ninein | wt | 22.16 | -3.49 |
|  |  | DNA polymerase alpha catalytic subunit |  |  |  |
|  |  | DNA-dependent protein kinase catalytic subunit |  |  |  |
|  |  | DNA topoisomerase 2-alpha |  |  |  |
|  |  | DNA topoisomerase 2-beta |  |  |  |
|  |  | Synaptonemal complex central element protein 1 |  |  |  |
|  |  | CDK5 regulatory subunit-associated protein 2 |  |  |  |
|  |  | Centromere protein J |  |  |  |
|  |  | WASH complex subunit 5 |  |  |  |
|  |  | ATPase family AAA domain-containing protein 5 |  |  |  |
|  |  | DNA replication ATP-dependent helicase/nuclease DNA2 |  |  |  |
|  |  | Kinesin-like protein KIF14 |  |  |  |
| *regulation of microtubule cytoskeleton organization* | 18 | InaD-like protein | wt | 9.42 | -3.28 |
|  |  | Dystonin |  |  |  |
|  |  | Ninein |  |  |  |
|  |  | Tropomyosin alpha-1 chain |  |  |  |
|  |  | Centrosomal protein of 70 kDa |  |  |  |
|  |  | E3 ubiquitin-protein ligase MYCBP2 |  |  |  |
|  |  | CDK5 regulatory subunit-associated protein 2 |  |  |  |
|  |  | Centromere protein J |  |  |  |
|  |  | WASH complex subunit 5 |  |  |  |
|  |  | Protein phosphatase Slingshot homolog 1 |  |  |  |
|  |  | Intraflagellar transport protein 88 homolog |  |  |  |
|  |  | Clusterin-associated protein 1 |  |  |  |
|  |  | Protein fantom |  |  |  |
|  |  | Receptor-type tyrosine-protein phosphatase S |  |  |  |
|  |  | Rab3 GTPase-activating protein non-catalytic subunit |  |  |  |
|  |  | Nesprin-2 |  |  |  |
|  |  | Spermatogenesis-associated protein 6 |  |  |  |
|  |  | Dynein regulatory complex subunit 7 |  |  |  |
| *nuclear migration* | 3 | Calmin | wt | 16.25 | -3.09 |
|  |  | Serine/threonine-protein kinase MRCK alpha |  |  |  |
|  |  | Nesprin-2 |  |  |  |
| *neuromuscular process* | 6 | Inositol 1,4,5-trisphosphate receptor type 1 | wt | 10.37 | -3.00 |
|  |  | Unconventional myosin-Va |  |  |  |
|  |  | Troponin T, fast skeletal muscle |  |  |  |
|  |  | Myosin-14 |  |  |  |
|  |  | Otoferlin |  |  |  |
|  |  | E3 ubiquitin-protein ligase MYCBP2 |  |  |  |
| *regulation of peptidase activity* | 9 | Collagen alpha-3(IV) chain | wt | 3.72 | -2.76 |
|  |  | Collagen alpha-1(VII) chain |  |  |  |
|  |  | Glyceraldehyde-3-phosphate dehydrogenase |  |  |  |
|  |  | Inter-alpha-trypsin inhibitor heavy chain H1 |  |  |  |
|  |  | Serpin B6 |  |  |  |
|  |  | Tissue factor pathway inhibitor |  |  |  |
|  |  | E3 ubiquitin-protein ligase SH3RF1 |  |  |  |
|  |  | Anthrax toxin receptor 1 |  |  |  |
|  |  | Uveal autoantigen with coiled-coil domains and ankyrin repeats |  |  |  |
| *RND2 GTPase cycle* | 10 | Dystonin | wt | 12.19 | -2.72 |
|  |  | Tyrosine-protein phosphatase non-receptor type 13 |  |  |  |
|  |  | E3 SUMO-protein ligase RanBP2 |  |  |  |
|  |  | T-lymphoma invasion and metastasis-inducing protein 2 |  |  |  |
|  |  | E3 ubiquitin-protein ligase SH3RF1 |  |  |  |
|  |  | Myosin-14 |  |  |  |
|  |  | Uveal autoantigen with coiled-coil domains and ankyrin repeats |  |  |  |
|  |  | Serine/threonine-protein kinase MRCK alpha |  |  |  |
|  |  | Coiled-coil domain-containing protein 187 |  |  |  |
|  |  | Kinesin-like protein KIF14 |  |  |  |
| *SUMOylation of DNA replication proteins* | 6 | E3 ubiquitin-protein ligase HERC2 | wt | 11.61 | -2.66 |
|  |  | E3 SUMO-protein ligase RanBP2 |  |  |  |
|  |  | DNA topoisomerase 2-alpha |  |  |  |
|  |  | DNA topoisomerase 2-beta |  |  |  |
|  |  | Scaffold attachment factor B1 |  |  |  |
|  |  | DNA helicase MCM8 |  |  |  |
| *intrinsic apoptotic signaling pathway* | 5 | Inositol 1,4,5-trisphosphate receptor type 1 | wt | 4.99 | -2.47 |
|  |  | Myb-binding protein 1A |  |  |  |
|  |  | DNA-dependent protein kinase catalytic subunit |  |  |  |
|  |  | Uveal autoantigen with coiled-coil domains and ankyrin repeats |  |  |  |
|  |  | ATPase family AAA domain-containing protein 5 |  |  |  |
| *regulation of type I interferon production* | 4 | Glyceraldehyde-3-phosphate dehydrogenase | wt | 6.08 | -2.36 |
|  |  | DNA polymerase alpha catalytic subunit |  |  |  |
|  |  | Receptor-type tyrosine-protein phosphatase S |  |  |  |
|  |  | Interferon-induced helicase C domain-containing protein 1 |  |  |  |
| *regulation of myelination* | 3 | RING finger protein 10 | wt | 7.99 | -2.20 |
|  |  | Polyphosphoinositide phosphatase |  |  |  |
|  |  | Kinesin-like protein KIF14 |  |  |  |
| *peptidyl-amino acid modification* | 11 | Polypeptide N-acetylgalactosaminyltransferase 3 | wt | 2.30 | -2.06 |
|  |  | Glyceraldehyde-3-phosphate dehydrogenase |  |  |  |
|  |  | DNA-dependent protein kinase catalytic subunit |  |  |  |
|  |  | E3 SUMO-protein ligase RanBP2 |  |  |  |
|  |  | 2-(3-amino-3-carboxypropyl)histidine synthase subunit 2 |  |  |  |
|  |  | Interferon-induced helicase C domain-containing protein 1 |  |  |  |
|  |  | E1A-binding protein p400 |  |  |  |
|  |  | Cytosolic carboxypeptidase 3 |  |  |  |
|  |  | E3 SUMO-protein ligase ZNF451 |  |  |  |
|  |  | Serine/threonine-protein kinase MRCK alpha |  |  |  |
|  |  | Microtubule-associated serine/threonine-protein kinase 4 |  |  |  |

**Supplementary Table 4**

**Fibre type classification.**

Overall fibre type per sample was calculated utilizing protein abundances from mass spectrometry and previously outlined myosin heavy chain percentages for each fibre type.


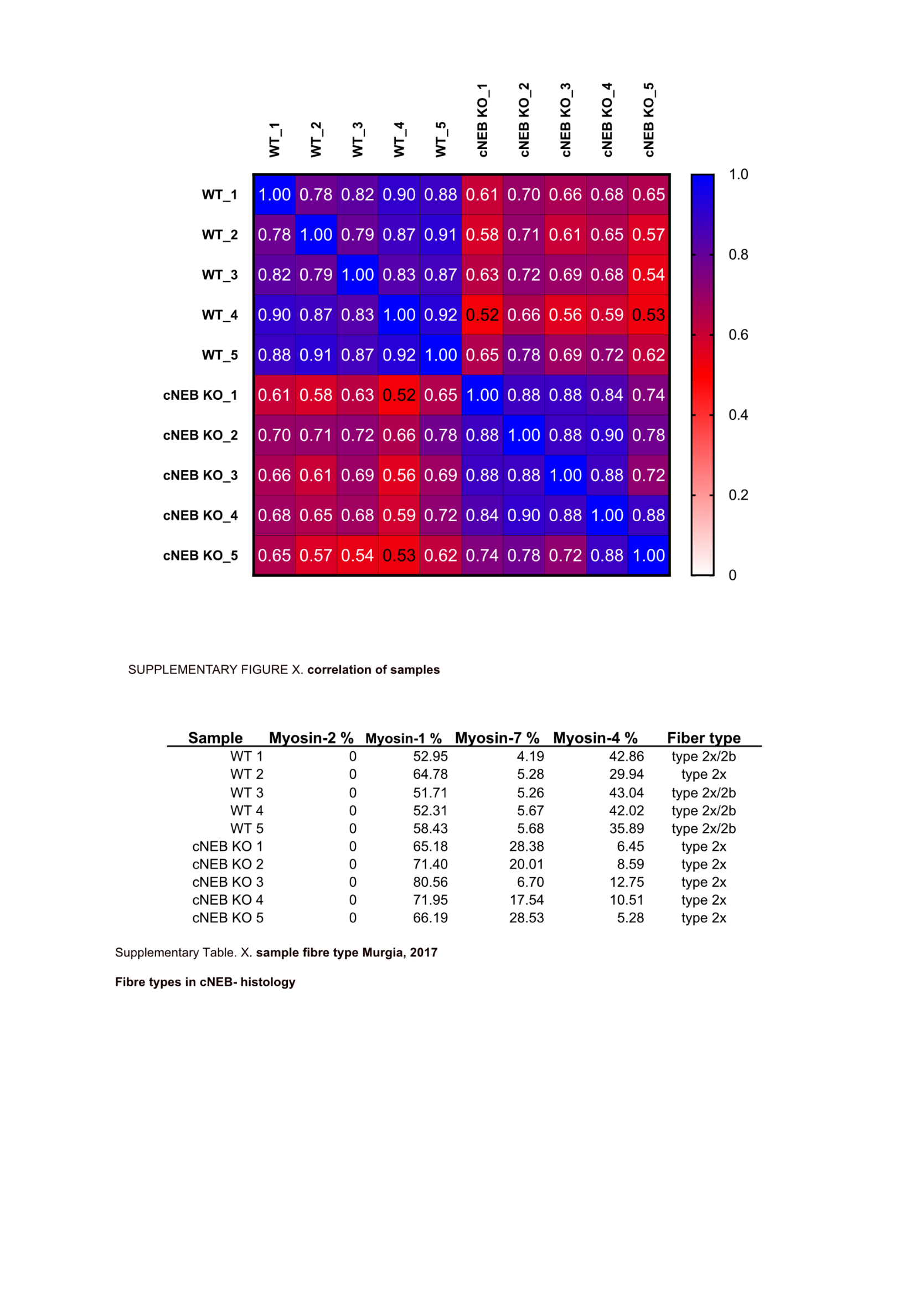


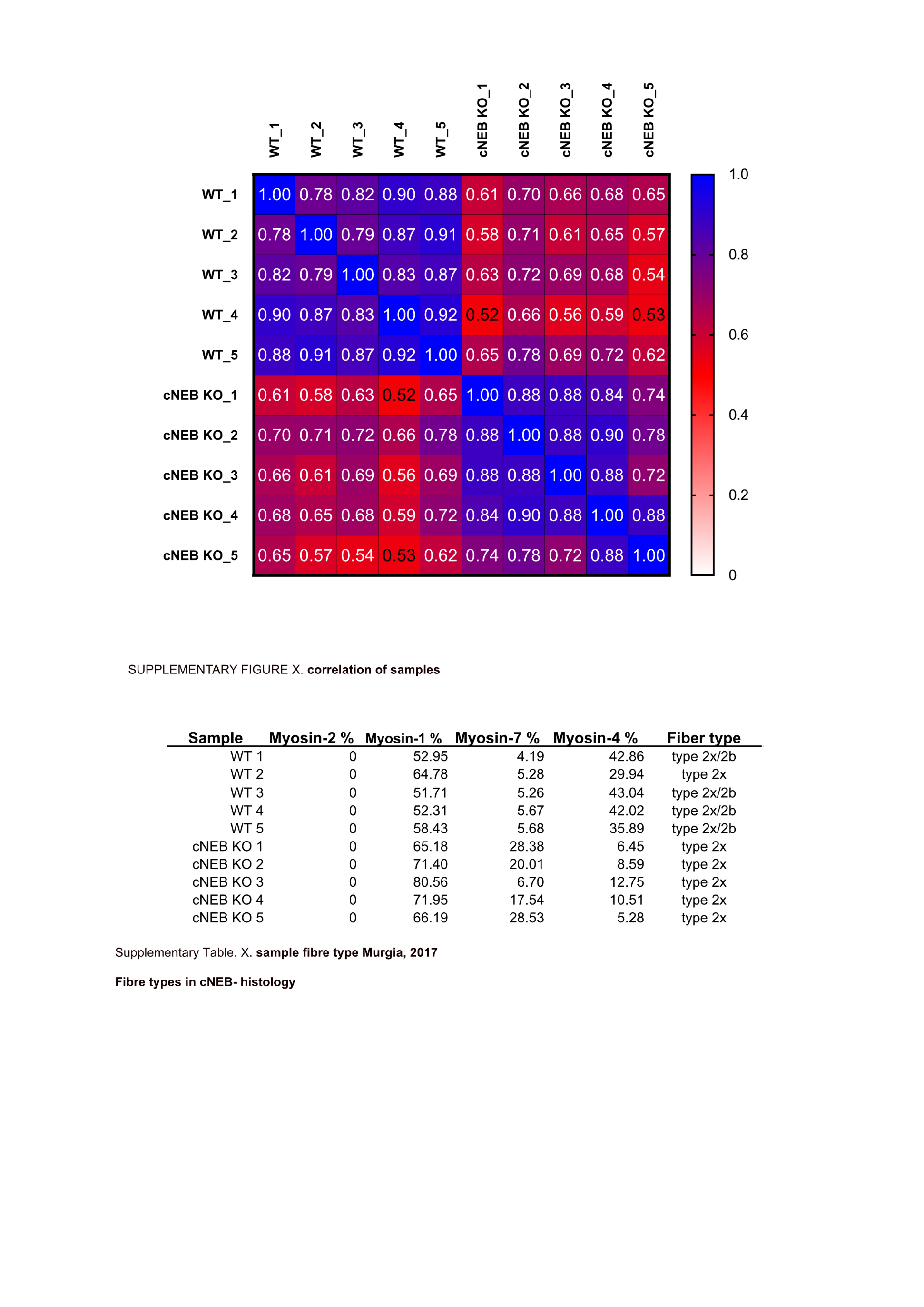


**Supplementary Table 5**

**All proteins detected in WT enzymatically disassociated fibres with and without piperine administration.**

All proteins detected during LC-MS/MS tandem mass spectrometry, following filtration of missing values. #protein abundance values. Significant upregulation in each experimental group determined based on *p* < 0.05.

| ***Gene name*** | ***Uniprot ID*** | ***Protein name*** | ***Control ^#^*** | ***cNEB ^#^*** | ***Log_2_ FC*** | ***p value*** | ***q value*** |
| --- | --- | --- | --- | --- | --- | --- | --- |
| **UPREGULATED IN CONTROL** | | | | | | | |
| Bpnt2 | Q80V26 | Golgi-resident adenosine 3',5'-bisphosphate 3'-phosphatase | 383.533 | 212.067 | 7.420 | 0.004 | 0.184 |
| Pin1 | Q9QUR7 | Peptidyl-prolyl cis-trans isomerase NIMA-interacting 1 | 296.400 | 156.933 | 7.102 | 0.001 | 0.124 |
| Spef2 | Q8C9J3 | Sperm flagellar protein 2 | 205.267 | 88.333 | 6.356 | 0.027 | 0.306 |
| Ahsg | P29699 | Alpha-2-HS-glycoprotein | 112.467 | 79.867 | 6.012 | 0.009 | 0.222 |
| Cep120 | Q7TSG1 | Centrosomal protein of 120 kDa | 432.533 | 259.333 | 7.872 | 0.011 | 0.222 |
| Grid1 | Q61627 | Glutamate receptor ionotropic, delta-1 | 443.867 | 295.933 | 8.037 | 0.016 | 0.250 |
| Sult1e1 | P49891 | Sulfotransferase 1E1 | 201.867 | 144.800 | 6.980 | 0.011 | 0.230 |
| Serpina1f | Q9DCQ7 | Alpha-1-antitrypsin 1-6 | 324.000 | 227.467 | 7.692 | 0.003 | 0.205 |
| Tmprss11e | Q5S248 | Transmembrane protease serine 11E | 438.067 | 310.467 | 8.110 | 0.008 | 0.248 |
| Fam184b | Q0KK56 | Protein FAM184B | 216.933 | 149.267 | 7.041 | 0.014 | 0.251 |
| Ttc23 | Q8CHY7 | Tetratricopeptide repeat protein 23 | 169.867 | 116.933 | 6.778 | < 0.001 | 0.054 |
| Rabgap1 | A2AWA9 | Rab GTPase-activating protein 1 | 481.000 | 355.733 | 8.232 | 0.031 | 0.333 |
| Mroh7 | A2AVR2 | Maestro heat-like repeat-containing protein family member 7 | 144.800 | 102.867 | 6.449 | 0.044 | 0.315 |
| Htra1 | Q9R118 | Serine protease HTRA1 | 278.200 | 195.733 | 7.477 | 0.009 | 0.257 |
| Lrp8 | Q924X6 | Low-density lipoprotein receptor-related protein 8 | 708.333 | 507.000 | 8.766 | 0.037 | 0.328 |
| Ccdc66 | Q6NS45 | Coiled-coil domain-containing protein 66 | 219.667 | 170.267 | 7.137 | 0.037 | 0.339 |
| Aox2 | Q5SGK3 | Aldehyde oxidase 2 | 204.867 | 146.733 | 7.062 | 0.013 | 0.246 |
| Ttll1 | Q91V51 | Probable tubulin polyglutamylase TTLL1 | 412.267 | 301.533 | 8.116 | 0.009 | 0.233 |
| Alb | P07724 | Albumin | 4424.200 | 3187.400 | 11.559 | 0.003 | 0.194 |
| H1.8 | Q8VIK3 | Histone H1.8 | 168.333 | 138.133 | 6.908 | 0.042 | 0.329 |
| Dtx2 | Q8R3P2 | Probable E3 ubiquitin-protein ligase DTX2 | 580.467 | 421.800 | 8.614 | 0.043 | 0.315 |
| Myo3b | Q1EG27 | Myosin-IIIb | 694.600 | 499.000 | 8.922 | 0.007 | 0.249 |
| Naa20 | P61600 | N-alpha-acetyltransferase 20 | 206.733 | 157.733 | 7.204 | 0.017 | 0.240 |
| Rif1 | Q6PR54 | Telomere-associated protein RIF1 | 229.133 | 181.133 | 7.383 | 0.016 | 0.262 |
| Nudt19 | P11930 | Nucleoside diphosphate-linked moiety X motif 19 | 269.200 | 202.333 | 7.593 | 0.017 | 0.238 |
| Ambp | Q07456 | Protein AMBP | 163.667 | 132.400 | 6.901 | 0.047 | 0.321 |
| Ampd2 | Q9DBT5 | AMP deaminase 2 | 756.067 | 572.933 | 9.091 | 0.018 | 0.238 |
| Heph | Q9Z0Z4 | Hephaestin | 174.667 | 136.733 | 6.990 | 0.036 | 0.348 |
| Mctp1 | E9PV86 | Multiple C2 and transmembrane domain-containing protein 1 | 269.733 | 215.133 | 7.646 | 0.049 | 0.309 |
| Ak6 | Q8VCP8 | Adenylate kinase isoenzyme 6 | 186.200 | 150.600 | 7.140 | 0.039 | 0.326 |
| Pold1 | P52431 | DNA polymerase delta catalytic subunit | 196.600 | 164.200 | 7.305 | 0.040 | 0.317 |
| Serpinc1 | P32261 | Antithrombin-III | 1085.000 | 915.867 | 9.814 | 0.047 | 0.314 |
|  |  |  |  |  |  |  |  |
| **UPREGULATED FOLLOWING PIPERINE ADMINISTRATION** | | | | | | | |
| Pvalb | P32848 | Parvalbumin alpha | 712.133 | 905.933 | 9.754 | 0.048 | 0.313 |
| Actc1 | P68033 | Actin, alpha cardiac muscle 1 | 7001.867 | 9077.267 | 13.081 | 0.027 | 0.318 |
| Hspa8 | P63017 | Heat shock cognate 71 kDa protein | 499.800 | 651.467 | 9.274 | 0.033 | 0.331 |
| Myh4 | Q5SX39 | Myosin-4 | 26619.600 | 33762.667 | 14.988 | 0.037 | 0.318 |
| Tecr | Q9CY27 | Very-long-chain enoyl-CoA reductase | 64.733 | 92.200 | 6.388 | 0.043 | 0.323 |
| Prkdc | P97313 | DNA-dependent protein kinase catalytic subunit | 98.067 | 136.667 | 6.990 | 0.032 | 0.326 |
| Acta1 | P68134 | Actin, alpha skeletal muscle | 232.933 | 367.733 | 8.342 | 0.023 | 0.279 |
| Pld6 | Q5SWZ9 | Mitochondrial cardiolipin hydrolase | 24.200 | 194.467 | 6.490 | < 0.001 | 0.092 |
|  |  |  |  |  |  |  |  |
| **NOT SIG** |  |  |  |  |  |  |  |
| Ttn | A2ASS6 | Titin | 3569.933 | 4338.467 | 0.225 | 0.502 | 0.736 |
| Atp2a1 | Q8R429 | Sarcoplasmic/endoplasmic reticulum calcium ATPase 1 | 5467.200 | 6215.933 | 0.177 | 0.254 | 0.544 |
| Pygm | Q9WUB3 | Glycogen phosphorylase, muscle form | 5286.733 | 6334.067 | 0.235 | 0.106 | 0.378 |
| Ckm | P07310 | Creatine kinase M-type | 8956.000 | 10303.067 | 0.171 | 0.180 | 0.467 |
| Actn3 | O88990 | Alpha-actinin-3 | 3514.000 | 4256.600 | 0.284 | 0.081 | 0.345 |
| Aldoa | P05064 | Fructose-bisphosphate aldolase A | 3259.600 | 3735.067 | 0.181 | 0.291 | 0.589 |
| Mybpc2 | Q5XKE0 | Myosin-binding protein C, fast-type | 1111.733 | 1250.067 | 0.198 | 0.130 | 0.405 |
| Tpm1 | P58771 | Tropomyosin alpha-1 chain | 4361.867 | 5119.467 | 0.310 | 0.168 | 0.461 |
| Tpm2 | P58774 | Tropomyosin beta chain | 1001.200 | 1167.667 | 0.282 | 0.193 | 0.475 |
| Eno3 | P21550 | Beta-enolase | 3135.667 | 3618.600 | 0.165 | 0.446 | 0.704 |
| Gapdh | P16858 | Glyceraldehyde-3-phosphate dehydrogenase | 1306.600 | 1450.200 | 0.097 | 0.604 | 0.792 |
| Pkm | P52480 | Pyruvate kinase PKM | 1025.067 | 1129.333 | 0.129 | 0.436 | 0.692 |
| Mylpf | P97457 | Myosin regulatory light chain 2, skeletal muscle isoform | 1872.133 | 2314.600 | 0.351 | 0.069 | 0.349 |
| Myl1 | P05977 | Myosin light chain 1/3, skeletal muscle isoform | 2001.467 | 2572.400 | 0.393 | 0.068 | 0.358 |
| Tnnt3 | Q9QZ47 | Troponin T, fast skeletal muscle | 1690.600 | 1988.000 | 0.252 | 0.162 | 0.454 |
| Des | P31001 | Desmin | 647.133 | 760.267 | 0.252 | 0.086 | 0.350 |
| Tpi1 | P17751 | Triosephosphate isomerase | 764.467 | 970.533 | 0.296 | 0.068 | 0.352 |
| Atp5f1b | P56480 | ATP synthase subunit beta, mitochondrial | 549.933 | 684.333 | 0.266 | 0.217 | 0.503 |
| Pfkm | P47857 | ATP-dependent 6-phosphofructokinase, muscle type | 1494.600 | 1578.000 | 0.061 | 0.594 | 0.784 |
| Atp5f1a | Q03265 | ATP synthase subunit alpha, mitochondrial | 361.000 | 441.400 | 0.288 | 0.106 | 0.382 |
| Pgam2 | O70250 | Phosphoglycerate mutase 2 | 1157.933 | 1329.000 | 0.166 | 0.403 | 0.662 |
| Ldha | P06151 | L-lactate dehydrogenase A chain | 1277.533 | 1428.333 | 0.131 | 0.491 | 0.731 |
| Slc25a4 | P48962 | ADP/ATP translocase 1 | 845.800 | 1109.667 | 0.378 | 0.074 | 0.350 |
| Casq1 | O09165 | Calsequestrin-1 | 1374.867 | 1770.667 | 0.339 | 0.072 | 0.352 |
| Myom1 | Q62234 | Myomesin-1 | 194.000 | 224.933 | 0.204 | 0.539 | 0.749 |
| Pgk1 | P09411 | Phosphoglycerate kinase 1 | 927.600 | 1035.400 | 0.169 | 0.396 | 0.656 |
| Mdh2 | P08249 | Malate dehydrogenase, mitochondrial | 322.533 | 411.933 | 0.324 | 0.116 | 0.382 |
| Pgm1 | Q9D0F9 | Phosphoglucomutase-1 | 189.333 | 199.933 | 0.069 | 0.678 | 0.818 |
| C3 | P01027 | Complement C3 | 1010.267 | 871.333 | -0.271 | 0.050 | 0.304 |
| Srl | Q7TQ48 | Sarcalumenin | 650.533 | 760.867 | 0.232 | 0.176 | 0.470 |
| Eno2 | P17183 | Gamma-enolase | 374.000 | 434.333 | 0.181 | 0.332 | 0.608 |
| Itih2 | Q61703 | Inter-alpha-trypsin inhibitor heavy chain H2 | 459.333 | 438.800 | -0.067 | 0.707 | 0.843 |
| Ryr1 | E9PZQ0 | #VALUE! | 454.200 | 410.933 | -0.113 | 0.641 | 0.794 |
| A2m | Q6GQT1 | Alpha-2-macroglobulin-P | 797.133 | 762.333 | -0.081 | 0.620 | 0.792 |
| Flnc | Q8VHX6 | Filamin-C | 133.533 | 140.933 | 0.096 | 0.765 | 0.890 |
| Fn1 | P11276 | Fibronectin | 560.133 | 499.733 | -0.188 | 0.359 | 0.618 |
| Gpi | P06745 | Glucose-6-phosphate isomerase | 467.867 | 531.800 | 0.177 | 0.307 | 0.593 |
| Ak1 | Q9R0Y5 | Adenylate kinase isoenzyme 1 | 225.267 | 292.867 | 0.314 | 0.304 | 0.597 |
| Vdac1 | Q60932 | Voltage-dependent anion-selective channel protein 1 | 315.000 | 381.667 | 0.304 | 0.435 | 0.694 |
| C4b | P01029 | Complement C4-B | 510.533 | 471.467 | -0.141 | 0.338 | 0.606 |
| Itih1 | Q61702 | Inter-alpha-trypsin inhibitor heavy chain H1 | 256.400 | 223.200 | -0.257 | 0.351 | 0.611 |
| Gsn | P13020 | Gelsolin | 576.133 | 508.133 | -0.220 | 0.464 | 0.709 |
| Tf | Q921I1 | Serotransferrin | 918.733 | 787.600 | -0.261 | 0.259 | 0.546 |
| Cryab | P23927 | Alpha-crystallin B chain | 755.000 | 687.867 | -0.102 | 0.504 | 0.735 |
| Tnni2 | P13412 | Troponin I, fast skeletal muscle | 407.267 | 520.333 | 0.327 | 0.579 | 0.765 |
| Gpd1 | P13707 | Glycerol-3-phosphate dehydrogenase [NAD(+)], cytoplasmic | 179.733 | 220.067 | 0.276 | 0.319 | 0.606 |
| Cs | Q9CZU6 | Citrate synthase, mitochondrial | 131.667 | 166.667 | 0.252 | 0.614 | 0.791 |
| Pzp | Q61838 | Pregnancy zone protein | 572.867 | 466.467 | -0.329 | 0.087 | 0.349 |
| F2 | P19221 | Prothrombin | 332.267 | 293.933 | -0.177 | 0.492 | 0.729 |
| Cp | Q61147 | Ceruloplasmin | 372.533 | 321.800 | -0.247 | 0.215 | 0.504 |
| Anxa6 | P14824 | Annexin A6 | 193.733 | 180.600 | -0.105 | 0.622 | 0.789 |
| Hsp90ab1 | P11499 | Heat shock protein HSP 90-beta | 511.333 | 605.200 | 0.256 | 0.192 | 0.478 |
| Hp | Q60574 | Haptoglobin | 225.600 | 194.867 | -0.229 | 0.076 | 0.341 |
| Rps27a | P62983 | Ubiquitin-40S ribosomal protein S27a | 581.867 | 789.467 | 0.418 | 0.063 | 0.359 |
| Serpind1 | P49182 | Heparin cofactor 2 | 231.467 | 187.200 | -0.218 | 0.379 | 0.640 |
| Ckmt2 | Q6P8J7 | Creatine kinase S-type, mitochondrial | 110.933 | 123.200 | 0.107 | 0.817 | 0.910 |
| Plg | P20918 | Plasminogen | 169.267 | 140.533 | -0.346 | 0.155 | 0.442 |
| Ampd1 | Q3V1D3 | AMP deaminase 1 | 437.933 | 461.733 | 0.089 | 0.678 | 0.822 |
| Sfn | O70456 | 14-3-3 protein sigma | 141.000 | 151.400 | 0.066 | 0.849 | 0.930 |
| Krt5 | Q922U2 | Keratin, type II cytoskeletal 5 | 378.400 | 439.467 | 0.185 | 0.269 | 0.563 |
| Eef1a2 | P62631 | Elongation factor 1-alpha 2 | 354.400 | 335.533 | -0.094 | 0.413 | 0.670 |
| Hpx | Q91X72 | Hemopexin | 381.333 | 310.400 | -0.257 | 0.314 | 0.601 |
| Serpinf2 | Q61247 | Alpha-2-antiplasmin | 44.733 | 44.333 | 0.043 | 0.805 | 0.907 |
| Park7 | Q99LX0 | Parkinson disease protein 7 homolog | 61.933 | 67.133 | 0.144 | 0.451 | 0.699 |
| Tmprss13 | Q5U405 | Transmembrane protease serine 13 | 1169.400 | 1170.533 | 0.126 | 0.613 | 0.794 |
| H4c1 | P62806 | Histone H4 | 79.600 | 96.933 | 0.260 | 0.330 | 0.613 |
| Uqcrc2 | Q9DB77 | Cytochrome b-c1 complex subunit 2, mitochondrial | 97.000 | 110.200 | 0.209 | 0.496 | 0.732 |
| Myoz3 | Q8R4E4 | Myozenin-3 | 69.600 | 71.533 | 0.100 | 0.772 | 0.894 |
| Nme2 | Q01768 | Nucleoside diphosphate kinase B | 117.467 | 114.800 | -0.006 | 0.982 | 1.000 |
| Uqcrc1 | Q9CZ13 | Cytochrome b-c1 complex subunit 1, mitochondrial | 214.467 | 235.933 | 0.131 | 0.560 | 0.748 |
| Rtn2 | O70622 | Reticulon-2 | 50.667 | 66.067 | 0.352 | 0.073 | 0.349 |
| Atp5pb | Q9CQQ7 | ATP synthase F(0) complex subunit B1, mitochondrial | 88.467 | 147.000 | 0.549 | 0.080 | 0.348 |
| Ogdh | Q60597 | 2-oxoglutarate dehydrogenase, mitochondrial | 241.267 | 306.000 | 0.356 | 0.167 | 0.462 |
| Itih4 | A6X935 | Inter alpha-trypsin inhibitor, heavy chain 4 | 87.533 | 81.267 | -0.139 | 0.617 | 0.790 |
| Kng1 | O08677 | Kininogen-1 | 140.733 | 132.333 | -0.116 | 0.530 | 0.745 |
| Ina | P46660 | Alpha-internexin | 147.133 | 166.600 | 0.191 | 0.335 | 0.605 |
| Afm | O89020 | Afamin | 471.200 | 352.733 | -0.434 | 0.139 | 0.421 |
| Sacs | Q9JLC8 | Sacsin | 280.667 | 214.533 | -0.495 | 0.100 | 0.374 |
| Apobec2 | Q9WV35 | C->U-editing enzyme APOBEC-2 | 223.467 | 329.333 | 0.421 | 0.111 | 0.381 |
| Filip1l | Q6P6L0 | Filamin A-interacting protein 1-like | 348.467 | 332.000 | -0.078 | 0.782 | 0.894 |
| Chmp5 | Q9D7S9 | Charged multivesicular body protein 5 | 436.000 | 349.200 | -0.381 | 0.329 | 0.616 |
| Hydin | Q80W93 | Hydrocephalus-inducing protein | 277.067 | 276.267 | 0.000 | 0.999 | 0.999 |
| Plekhh1 | Q80TI1 | Pleckstrin homology domain-containing family H member 1 | 203.333 | 158.000 | -0.306 | 0.369 | 0.627 |
| Brd9 | Q3UQU0 | Bromodomain-containing protein 9 | 277.533 | 193.733 | -0.550 | 0.067 | 0.363 |
| Dnah8 | Q91XQ0 | Dynein heavy chain 8, axonemal | 589.800 | 461.000 | -0.405 | 0.219 | 0.504 |
| Rpusd4 | Q9CWX4 | Mitochondrial RNA pseudouridine synthase Rpusd4 | 141.400 | 162.867 | 0.188 | 0.298 | 0.598 |
| Cep350 | E9Q309 | Centrosome-associated protein 350 | 177.067 | 150.667 | -0.284 | 0.252 | 0.548 |
| Cenpe | Q6RT24 | Centromere-associated protein E | 270.200 | 253.400 | 0.033 | 0.952 | 0.991 |
| Afg3l2 | Q8JZQ2 | AFG3-like protein 2 | 350.733 | 273.267 | -0.489 | 0.347 | 0.614 |
| Odf2l | Q9D478 | Protein BCAP | 826.467 | 815.200 | 0.027 | 0.886 | 0.960 |
| Psd3 | Q2PFD7 | PH and SEC7 domain-containing protein 3 | 572.600 | 393.733 | -0.521 | 0.306 | 0.596 |
| Ttyh3 | Q6P5F7 | Protein tweety homolog 3 | 297.800 | 322.200 | -0.021 | 0.954 | 0.989 |
| Cep290 | Q6A078 | Centrosomal protein of 290 kDa | 276.867 | 207.667 | -0.496 | 0.154 | 0.444 |
| Map1b | P14873 | Microtubule-associated protein 1B | 156.200 | 129.733 | -0.404 | 0.092 | 0.354 |
| Klk1b1 | P00755 | Kallikrein 1-related peptidase b1 | 171.533 | 174.000 | 0.013 | 0.942 | 0.984 |
| Nf1 | Q04690 | Neurofibromin | 222.467 | 213.667 | -0.117 | 0.702 | 0.841 |
| Ces1e | Q64176 | Carboxylesterase 1E | 424.267 | 380.933 | -0.203 | 0.640 | 0.796 |
| Rbm14 | Q8C2Q3 | RNA-binding protein 14 | 204.733 | 224.800 | -0.033 | 0.914 | 0.970 |
| Syne2 | Q6ZWQ0 | Nesprin-2 | 693.267 | 539.200 | -0.419 | 0.213 | 0.504 |
| Tp63 | O88898 | Tumor protein 63 | 555.400 | 507.200 | -0.195 | 0.553 | 0.750 |
| Ryr3 | A2AGL3 | Ryanodine receptor 3 | 76.200 | 68.200 | -0.081 | 0.741 | 0.868 |
| Dync2h1 | Q45VK7 | Cytoplasmic dynein 2 heavy chain 1 | 164.067 | 166.067 | 0.279 | 0.612 | 0.796 |
| Ubr4 | A2AN08 | E3 ubiquitin-protein ligase UBR4 | 224.533 | 205.000 | -0.089 | 0.797 | 0.903 |
| Fsip2 | A2ARZ3 | Fibrous sheath-interacting protein 2 | 724.467 | 751.000 | -0.027 | 0.921 | 0.974 |
| Dnah2 | P0C6F1 | Dynein heavy chain 2, axonemal | 275.867 | 356.133 | 0.256 | 0.537 | 0.750 |
| Thumpd1 | Q99J36 | THUMP domain-containing protein 1 | 124.533 | 99.800 | -0.299 | 0.368 | 0.630 |
| Tceal3 | Q8R0A5 | Transcription elongation factor A protein-like 3 | 184.800 | 154.667 | -0.356 | 0.064 | 0.356 |
| Ranbp2 | Q9ERU9 | E3 SUMO-protein ligase RanBP2 | 374.600 | 314.533 | -0.278 | 0.050 | 0.310 |
| Prpf6 | Q91YR7 | Pre-mRNA-processing factor 6 | 787.467 | 626.067 | -0.490 | 0.223 | 0.506 |
| Znf318 | Q99PP2 | Zinc finger protein 318 | 612.133 | 622.200 | 0.194 | 0.464 | 0.705 |
| Tex11 | Q14AT2 | Testis-expressed protein 11 | 197.867 | 125.733 | -0.226 | 0.515 | 0.744 |
| Cda | P56389 | Cytidine deaminase | 909.600 | 775.467 | -0.240 | 0.178 | 0.471 |
| Spen | Q62504 | Msx2-interacting protein | 592.133 | 489.000 | -0.269 | 0.348 | 0.610 |
| Abca13 | Q5SSE9 | ATP-binding cassette sub-family A member 13 | 238.867 | 213.867 | -0.310 | 0.448 | 0.702 |
| Auh | Q9JLZ3 | Methylglutaconyl-CoA hydratase, mitochondrial | 754.933 | 649.533 | -0.398 | 0.326 | 0.616 |
| Sdk2 | Q6V4S5 | Protein sidekick-2 | 145.200 | 156.267 | 0.021 | 0.939 | 0.985 |
| Kdm2b | Q6P1G2 | Lysine-specific demethylase 2B | 320.733 | 292.333 | -0.148 | 0.736 | 0.870 |
| Rabl6 | Q5U3K5 | Rab-like protein 6 | 111.533 | 158.000 | 0.749 | 0.179 | 0.469 |
| Smarcc1 | P97496 | SWI/SNF complex subunit SMARCC1 | 103.733 | 104.400 | 0.014 | 0.964 | 0.992 |
| Map4k4 | P97820 | Mitogen-activated protein kinase kinase kinase kinase 4 | 236.600 | 291.067 | 0.287 | 0.270 | 0.560 |
| Utp14b | Q6EJB6 | U3 small nucleolar RNA-associated protein 14 homolog B | 175.200 | 141.333 | -0.276 | 0.543 | 0.747 |
| Letm1 | Q9Z2I0 | Mitochondrial proton/calcium exchanger protein | 667.800 | 613.867 | -0.240 | 0.520 | 0.743 |
| Apcs | P12246 | Serum amyloid P-component | 351.333 | 271.067 | -0.401 | 0.151 | 0.442 |
| Ccdc90b | Q8C3X2 | Coiled-coil domain-containing protein 90B, mitochondrial | 116.467 | 96.133 | -0.320 | 0.116 | 0.379 |
| Itpr2 | Q9Z329 | Inositol 1,4,5-trisphosphate receptor type 2 | 347.800 | 329.133 | -0.075 | 0.778 | 0.897 |
| Spart | Q8R1X6 | Spartin | 469.600 | 474.800 | -0.029 | 0.880 | 0.957 |
| Prkar2b | P31324 | cAMP-dependent protein kinase type II-beta regulatory subunit | 487.467 | 372.933 | -0.424 | 0.274 | 0.559 |
| Ylpm1 | Q9R0I7 | YLP motif-containing protein 1 | 82.933 | 114.667 | 0.179 | 0.634 | 0.798 |
| Ppl | Q9R269 | Periplakin | 155.267 | 138.733 | -0.012 | 0.976 | 1.000 |
| Ice1 | E9Q286 | Little elongation complex subunit 1 | 231.067 | 200.600 | -0.186 | 0.485 | 0.729 |
| Eif5b | Q05D44 | Eukaryotic translation initiation factor 5B | 85.333 | 89.067 | -0.006 | 0.988 | 0.995 |
| Otc | P11725 | Ornithine transcarbamylase, mitochondrial | 136.000 | 127.133 | -0.023 | 0.897 | 0.963 |
| Clstn1 | Q9EPL2 | Calsyntenin-1 | 490.533 | 365.667 | -0.384 | 0.121 | 0.389 |
| Znf518b | B2RRE4 | Zinc finger protein 518B | 299.600 | 266.067 | -0.234 | 0.661 | 0.816 |
| Cdh23 | Q99PF4 | Cadherin-23 | 86.000 | 114.867 | 0.385 | 0.146 | 0.434 |
| Hk1 | P17710 | Hexokinase-1 | 149.067 | 141.200 | -0.082 | 0.809 | 0.909 |
| Ror2 | Q9Z138 | Tyrosine-protein kinase transmembrane receptor ROR2 | 434.733 | 237.800 | -0.735 | 0.060 | 0.352 |
| Ktn1 | Q61595 | Kinectin | 265.467 | 291.200 | 0.282 | 0.518 | 0.745 |
| Apc2 | Q9Z1K7 | Adenomatous polyposis coli protein 2 | 118.933 | 129.600 | -0.021 | 0.957 | 0.989 |
| Dele1 | Q9DCV6 | DAP3-binding cell death enhancer 1 | 451.400 | 551.533 | 0.230 | 0.207 | 0.494 |
| Ros1 | Q78DX7 | Proto-oncogene tyrosine-protein kinase ROS | 159.800 | 122.200 | -0.395 | 0.236 | 0.523 |
| Med12l | Q8BQM9 | Mediator Complex Subunit 12L | 346.733 | 258.200 | -0.491 | 0.108 | 0.378 |
| Rprd2 | Q6NXI6 | Regulation of nuclear pre-mRNA domain-containing protein 2 | 162.867 | 171.400 | -0.038 | 0.923 | 0.971 |
| Eif3h | Q91WK2 | Eukaryotic translation initiation factor 3 subunit H | 164.933 | 188.600 | 0.195 | 0.671 | 0.825 |
| Grik5 | Q61626 | Glutamate receptor ionotropic, kainate 5 | 202.067 | 146.533 | -0.489 | 0.302 | 0.596 |
| Znf830 | Q8R1N0 | Zinc finger protein 830 | 136.000 | 127.133 | -0.023 | 0.897 | 0.959 |
| Rgs6 | Q9Z2H2 | Regulator of G-protein signaling 6 | 185.000 | 160.667 | -0.293 | 0.353 | 0.610 |
| Ulk1 | O70405 | Serine/threonine-protein kinase ULK1 | 415.000 | 440.067 | 0.075 | 0.740 | 0.871 |
| Septin7 | O55131 | Septin-7 | 114.067 | 118.000 | 0.023 | 0.893 | 0.963 |
| Ash1l | Q99MY8 | Histone-lysine N-methyltransferase ASH1L | 1019.333 | 637.933 | -0.612 | 0.172 | 0.465 |
| Nedd1 | P33215 | Protein NEDD1 | 299.867 | 385.800 | 0.356 | 0.387 | 0.646 |
| Kctd19 | Q562E2 | BTB/POZ domain-containing protein KCTD19 | 644.067 | 637.200 | -0.016 | 0.835 | 0.922 |
| Sphkap | Q6NSW3 | A-kinase anchor protein SPHKAP | 181.200 | 201.467 | 0.147 | 0.756 | 0.882 |
| Inpp5f | Q8CDA1 | Phosphatidylinositide phosphatase SAC2 | 563.933 | 422.733 | -0.445 | 0.092 | 0.358 |
| Nsd1 | O88491 | Histone-lysine N-methyltransferase, H3 lysine-36 specific | 109.733 | 152.000 | 0.087 | 0.794 | 0.904 |
| Copg1 | Q9QZE5 | Coatomer subunit gamma-1 | 193.400 | 150.200 | -0.525 | 0.104 | 0.382 |
| Diaph1 | O08808 | Protein diaphanous homolog 1 | 93.667 | 110.067 | 0.143 | 0.674 | 0.824 |
| Sos1 | Q62245 | Son of sevenless homolog 1 | 279.733 | 269.333 | -0.166 | 0.726 | 0.861 |
| Dicer1 | Q8R418 | Endoribonuclease Dicer | 180.200 | 200.400 | 0.057 | 0.821 | 0.910 |
| Atg2b | Q80XK6 | Autophagy-related protein 2 homolog B | 320.800 | 268.267 | -0.328 | 0.076 | 0.345 |
| Ppip5k2 | Q6ZQB6 | Histidine Acid Phosphatase Domain Containing 1 | 245.933 | 182.400 | -0.421 | 0.202 | 0.487 |
| Map2 | P20357 | Microtubule-associated protein 2 | 285.867 | 295.600 | 0.136 | 0.540 | 0.747 |
| Abcg2 | Q7TMS5 | Broad substrate specificity ATP-binding cassette transporter ABCG2 | 425.333 | 464.933 | -0.004 | 0.984 | 0.998 |
| Smc2 | Q8CG48 | Structural maintenance of chromosomes protein 2 | 280.133 | 351.400 | 0.294 | 0.087 | 0.344 |
| Ppp1r12a | Q9DBR7 | Protein phosphatase 1 regulatory subunit 12A | 219.200 | 238.000 | 0.060 | 0.779 | 0.894 |
| Ik | Q9Z1M8 | Protein Red | 102.400 | 80.733 | -0.006 | 0.987 | 0.998 |
| Kiaa0100 | Q5SYL3 | Protein KIAA0100 | 704.133 | 581.933 | -0.405 | 0.249 | 0.547 |
| Ebna1bp2 | Q9D903 | Probable rRNA-processing protein EBP2 | 139.533 | 116.067 | -0.349 | 0.131 | 0.402 |
| Plin2 | P43883 | Perilipin-2 | 217.667 | 178.533 | -0.454 | 0.124 | 0.391 |
| Arhgap25 | Q8BYW1 | Rho GTPase-activating protein 25 | 171.267 | 134.867 | -0.343 | 0.184 | 0.472 |
| Polr3b | P59470 | DNA-directed RNA polymerase III subunit RPC2 | 409.400 | 362.067 | -0.229 | 0.634 | 0.794 |
| #VALUE! | Q3V0E1 | Uncharacterized protein C9orf131 homolog | 70.467 | 84.800 | 0.296 | 0.334 | 0.607 |
| Prex1 | Q69ZK0 | PtdIns(3,4,5)-Dependent Rac Exchanger 1 | 110.267 | 127.333 | 0.218 | 0.557 | 0.748 |
| Itgb3bp | Q9CQ82 | Centromere protein R | 155.200 | 193.333 | 0.233 | 0.228 | 0.511 |
| Zfhx4 | Q9JJN2 | Zinc finger homeobox protein 4 | 108.400 | 112.800 | 0.156 | 0.678 | 0.825 |
| Znf451 | Q8C0P7 | E3 SUMO-protein ligase ZNF451 | 161.667 | 145.733 | -0.216 | 0.426 | 0.683 |
| Sh3gl1 | Q62419 | Endophilin-A2 | 134.067 | 118.600 | -0.228 | 0.405 | 0.662 |
| Ppp1r10 | Q80W00 | Serine/threonine-protein phosphatase 1 regulatory subunit 10 | 188.533 | 167.200 | -0.210 | 0.553 | 0.754 |
| Hspa4 | Q61316 | Heat shock 70 kDa protein 4 | 189.267 | 138.600 | -0.486 | 0.075 | 0.348 |
| Rnf212b | D3Z423 | RING finger protein 212B | 195.533 | 158.867 | -0.393 | 0.270 | 0.555 |
| Pak6 | Q3ULB5 | Serine/threonine-protein kinase PAK 6 | 424.733 | 339.667 | -0.303 | 0.605 | 0.791 |
| Topaz1 | E5FYH1 | Protein TOPAZ1 | 133.533 | 106.533 | -0.345 | 0.202 | 0.490 |
| Rb1cc1 | Q9ESK9 | RB1-inducible coiled-coil protein 1 | 105.867 | 88.800 | -0.323 | 0.116 | 0.388 |
| Kdm4c | Q8VCD7 | Lysine-specific demethylase 4C | 190.467 | 184.200 | -0.006 | 0.979 | 1.000 |
| Chst3 | O88199 | Carbohydrate sulfotransferase 3 | 300.867 | 378.733 | 0.268 | 0.221 | 0.505 |
| Pus10 | Q9D3U0 | tRNA pseudouridine synthase Pus10 | 174.733 | 128.000 | -0.077 | 0.813 | 0.908 |
| Mrpl58 | Q8R035 | Peptidyl-tRNA hydrolase ICT1, mitochondrial | 236.800 | 293.000 | 0.225 | 0.454 | 0.698 |
| Fbxo22 | Q78JE5 | F-box only protein 22 | 113.733 | 93.533 | -0.148 | 0.566 | 0.752 |
| Thap1 | Q8CHW1 | THAP domain-containing protein 1 | 294.867 | 234.933 | -0.210 | 0.628 | 0.793 |
| Adra2b | P30545 | Alpha-2B adrenergic receptor | 282.733 | 228.667 | -0.315 | 0.081 | 0.339 |
| Taf1b | P97358 | TATA Box-Binding Protein-Associated Factor 1B | 299.867 | 210.733 | -0.464 | 0.112 | 0.379 |
| Rab3gap2 | Q8BMG7 | Rab3 GTPase-activating protein non-catalytic subunit | 425.133 | 282.200 | -0.522 | 0.099 | 0.374 |
| Nr1d1 | Q3UV55 | Nuclear receptor subfamily 1 group D member 1 | 109.067 | 119.867 | 0.096 | 0.682 | 0.819 |
| Pla2g6 | P97819 | 85/88 kDa calcium-independent phospholipase A2 | 584.867 | 463.333 | -0.320 | 0.254 | 0.548 |
| Mkks | Q9JI70 | MKKS Centrosomal Shuttling Protein | 263.133 | 222.400 | -0.153 | 0.468 | 0.707 |
| ptchd1 | Q14B62 | Patched domain-containing protein 1 | 166.600 | 124.400 | -0.442 | 0.330 | 0.610 |
| Clec3a | Q9EPW4 | C-type lectin domain family 3 member A | 142.333 | 170.400 | 0.202 | 0.524 | 0.745 |
| Nckap5l | Q6GQX2 | Nck-associated protein 5-like | 398.000 | 341.067 | -0.247 | 0.145 | 0.436 |
| Ehd4 | Q9EQP2 | EH domain-containing protein 4 | 301.200 | 199.000 | -0.678 | 0.083 | 0.348 |
| Znf423 | Q80TS5 | Zinc finger protein 423 | 305.800 | 322.667 | 0.092 | 0.554 | 0.747 |
| Dact2 | Q7TN08 | Dapper homolog 2 | 247.467 | 255.800 | 0.171 | 0.489 | 0.732 |
| Vps33b | P59016 | Vacuolar protein sorting-associated protein 33B | 173.600 | 156.933 | -0.225 | 0.185 | 0.470 |
| Sike1 | Q9CPR7 | Suppressor of IKBKE 1 | 269.267 | 225.067 | -0.182 | 0.510 | 0.740 |
| Ankrd13c | Q3UX43 | Ankyrin repeat domain-containing protein 13C | 413.267 | 345.533 | -0.226 | 0.381 | 0.639 |
| Abca9 | Q8K449 | ATP-binding cassette sub-family A member 9 | 182.800 | 172.000 | -0.104 | 0.451 | 0.702 |
| Lonrf1 | D3YY23 | LON peptidase N-terminal domain and RING finger protein 1 | 436.600 | 319.733 | -0.410 | 0.187 | 0.471 |
| Dleu7 | Q8CHZ8 | Leukemia-associated protein 7 homolog | 195.133 | 151.800 | -0.443 | 0.069 | 0.345 |
| Rsph14 | Q9D3W1 | Radial spoke head 14 homolog | 58.867 | 61.600 | 0.034 | 0.839 | 0.923 |
| Thap2 | Q9D305 | THAP domain-containing protein 2 | 141.867 | 133.467 | -0.043 | 0.897 | 0.956 |
| Rnaset2a | C0HKG5 | Ribonuclease T2-A | 250.267 | 253.600 | 0.077 | 0.878 | 0.958 |
| Frat2 | Q8K025 | GSK-3-binding protein FRAT2 | 129.333 | 149.733 | -0.004 | 0.990 | 0.993 |
| Stac | P97306 | SH3 and cysteine-rich domain-containing protein | 488.200 | 367.333 | -0.288 | 0.526 | 0.744 |
| Ghrhr | P32082 | Growth hormone-releasing hormone receptor | 205.067 | 176.533 | -0.242 | 0.254 | 0.540 |
| Prkcz | Q02956 | Protein kinase C zeta type | 170.867 | 160.867 | -0.180 | 0.342 | 0.609 |
| Flywch2 | Q9CQE9 | FLYWCH family member 2 | 111.333 | 197.133 | 0.719 | 0.123 | 0.394 |
| Sec31b | Q3TZ89 | Protein transport protein Sec31B | 120.067 | 160.200 | 0.321 | 0.299 | 0.595 |
| Alyref2 | Q9JJW6 | Aly/REF export factor 2 | 522.267 | 526.000 | -0.263 | 0.414 | 0.669 |
| slx1 | B4KBJ0 | Structure-specific endonuclease subunit SLX1 homolog | 243.400 | 225.000 | -0.191 | 0.552 | 0.757 |

**Supplementary Table 6**

**Uniprot ligand binding for significant proteins detected in WT enzymatically disassociated fibres with and without piperine administration**

Likely ligand binding for all significant proteins (p < 0.05) obtained with and without piperine administration determined via the DAVID bioinformatic database.

| ***Gene name*** | ***Uniprot ID*** | ***Protein name*** | ***Uniprot ligand binding*** |
| --- | --- | --- | --- |
| **UPREGULATED IN CONTROL** | | | |
| Mctp1 | E9PV86 | multiple C2 domains, transmembrane 1 | KW-0106~Calcium  KW-0479~Metal-binding |
| Alb | P07724 | albumin | KW-0106~Calcium  KW-0186~Copper  KW-0446~Lipid-binding  KW-0479~Metal-binding  KW-0862~Zinc |
| Nudt19 | P11930 | nudix (nucleoside diphosphate linked moiety X)-type motif 19 | KW-0460~Magnesium  KW-0464~Manganese  KW-0479~Metal-binding |
| Sult1e1 | P49891 | sulfotransferase family 1E, member 1 | KW-0446~Lipid-binding  KW-0754~Steroid-binding |
| Pold1 | P52431 | polymerase (DNA directed), delta 1, catalytic subunit | KW-0004~4Fe-4S  KW-0408~Iron  KW-0411~Iron-sulfur  KW-0479~Metal-binding  KW-0862~Zinc |
| Ambp | Q07456 | alpha 1 microglobulin/bikunin precursor | KW-0157~Chromophore |
| Myo3b | Q1EG27 | myosin IIIB | KW-0067~ATP-binding  KW-0547~Nucleotide-binding, |
| Tmprss11e | Q5SGK3 | aldehyde oxidase 2 | KW-0001~2Fe-2S  KW-0274~FAD  KW-0285~Flavoprotein  KW-0408~Iron  KW-0411~Iron-sulfur  KW-0479~Metal-binding  KW-0500~Molybdenum |
| Bpnt2 | Q80V26 | 3'(2'), 5'-bisphosphate nucleotidase 2 | KW-0460~Magnesium  KW-0479~Metal-binding |
| Dtx2 | Q8R3P2 | deltex 2, E3 ubiquitin ligase | KW-0479~Metal-binding  KW-0862~Zinc |
| Ak6 | Q8VCP8 | adenylate kinase 6 | KW-0067~ATP-binding  KW-0547~Nucleotide-binding |
| Ttll1 | Q91V51 | tubulin tyrosine ligase-like 1 | KW-0067~ATP-binding  KW-0460~Magnesium  KW-0479~Metal-binding  KW-0547~Nucleotide-binding |
| Lrp8 | Q924X6 | low density lipoprotein receptor-related protein 8, apolipoprotein e receptor | KW-0106~Calcium  KW-0479~Metal-binding |
| Ampd2 | Q9DBT5 | adenosine monophosphate deaminase 2 | KW-0479~Metal-binding  KW-0862~Zinc |
| Heph | Q9Z0Z4 | hephaestin | KW-0186~Copper  KW-0408~Iron  KW-0479~Metal-binding |
| **UPREGULATED FOLLOWING PIPERINE ADMINISTRATION** | | | |
| Pvalb | P32848 | parvalbumin | KW-0106~Calcium  KW-0479~Metal-binding |
| Hspa8 | P63017 | heat shock protein 8 | KW-0067~ATP-binding  KW-0547~Nucleotide-binding |
| Actc1 | P68033 | actin, alpha, cardiac muscle 1 | KW-0067~ATP-binding  KW-0547~Nucleotide-binding |
| Acta1 | P68134 | actin alpha 1, skeletal muscle | KW-0067~ATP-binding  KW-0547~Nucleotide-binding |
| Prkdc | P97313 | protein kinase, DNA activated, catalytic polypeptide( | KW-0067~ATP-binding  KW-0547~Nucleotide-binding |
| Pld6 | Q5SWZ9 | phospholipase D family, member 6 | KW-0479~Metal-binding  KW-0862~Zinc |
| Myh4 | Q5SX39 | myosin, heavy polypeptide 4, skeletal muscle | KW-0067~ATP-binding  KW-0547~Nucleotide-binding |
| Tecr | Q9CY27 | trans-2,3-enoyl-CoA reductase | KW-0521~NADP |
| Pvalb | P32848 | parvalbumin | KW-0106~Calcium  KW-0479~Metal-binding |
